# Integrating EMR-Linked and *In Vivo* Functional Genetic Data to Identify New Genotype-Phenotype Associations

**DOI:** 10.1371/journal.pone.0100322

**Published:** 2014-06-20

**Authors:** Jonathan D. Mosley, Sara L. Van Driest, Peter E. Weeke, Jessica T. Delaney, Quinn S. Wells, Lisa Bastarache, Dan M. Roden, Josh C. Denny

**Affiliations:** 1 Department of Medicine, Vanderbilt University, Nashville, Tennessee, United States of America; 2 Department of Pediatrics, Vanderbilt University, Nashville, Tennessee, United States of America; 3 Biomedical Informatics, Vanderbilt University, Nashville, Tennessee, United States of America; Children's National Medical Center, Washington, United States of America

## Abstract

The coupling of electronic medical records (EMR) with genetic data has created the potential for implementing reverse genetic approaches in humans, whereby the function of a gene is inferred from the shared pattern of morbidity among homozygotes of a genetic variant. We explored the feasibility of this approach to identify phenotypes associated with low frequency variants using Vanderbilt's EMR-based BioVU resource. We analyzed 1,658 low frequency non-synonymous SNPs (nsSNPs) with a minor allele frequency (MAF)<10% collected on 8,546 subjects. For each nsSNP, we identified diagnoses shared by at least 2 minor allele homozygotes and with an association p<0.05. The diagnoses were reviewed by a clinician to ascertain whether they may share a common mechanistic basis. While a number of biologically compelling clinical patterns of association were observed, the frequency of these associations was identical to that observed using genotype-permuted data sets, indicating that the associations were likely due to chance. To refine our analysis associations, we then restricted the analysis to 711 nsSNPs in genes with phenotypes in the On-line Mendelian Inheritance in Man (OMIM) or knock-out mouse phenotype databases. An initial comparison of the EMR diagnoses to the known *in vivo* functions of the gene identified 25 candidate nsSNPs, 19 of which had significant genotype-phenotype associations when tested using matched controls. Twleve of the 19 nsSNPs associations were confirmed by a detailed record review. Four of 12 nsSNP-phenotype associations were successfully replicated in an independent data set: thrombosis (*F5*,rs6031), seizures/convulsions (*GPR98*,rs13157270), macular degeneration (*CNGB3*,rs3735972), and GI bleeding (*HGFAC*,rs16844401). These analyses demonstrate the feasibility and challenges of using reverse genetics approaches to identify novel gene-phenotype associations in human subjects using low frequency variants. As increasing amounts of rare variant data are generated from modern genotyping and sequence platforms, model organism data may be an important tool to enable discovery.

## Introduction

Electronic medical record (EMR) systems store an increasing amount of clinical, laboratory and biometric data generated by health care systems. These data offer opportunities to explore risk factors for diseases, the inter-relationships among disease entities, and determinants of treatment response in large populations of individuals [1]. EMR data integrated with DNA repositories can also be utilized to identify genetic contributions to human disease risk and treatment response [2]–[7]. The spectrum of disease entities collected in EMRs has also enabled large-scale bioinformatics approaches such as Phenome-Wide Association Study (PheWAS), which searches in a disease-agnostic fashion for associations between common polymorphisms and hundreds of clinical diseases, identified using billing codes [8], [9]. The success of PheWAS approaches for common variants suggests that similar EMR-based approaches may identify associations with low frequency or rare variants [4], [10], [11].

Experimental model systems such as mouse models have been successful in assigning functionality to genes through the use of reverse genetics approaches, which identify phenotypes associated with a known genetic lesion [12], [13]. Structured data derived from mouse studies are increasingly available through large coordinated efforts such as the Knock-out Mouse Project (KOMP) [14] and the Mouse Phenome Database [15]. These data sources provide a rich resource for generating biologically-relevant clinical hypotheses based on observations of model organisms that can now be tested in a real life setting using large EMRs coupled with DNA repositories, such as the Vanderbilt BioVU resource [16].

Rare and low frequency single nucleotide polymorphisms (SNPs) are appealing candidates to explain much of the variation in human traits that cannot be accounted for by common polymorphisms [17]. However, associating rare variants to disease represents a considerable methodological challenge and remains an area of active research [18], [19]. From an epidemiological standpoint, low frequency variants are of particular interest because they can be associated with large effect sizes, enabling genetic approaches to discovery [20]–[22].

The coupling of EMR data with rare variant genetic data has created the potential for implementing reverse genetics approaches in humans, whereby the function of a gene is inferred from the shared pattern of morbidity among homozygotes of a genetic variant [23]. We explored the feasibility of this idea using 1,658 low frequency non-synonymous SNP (nsSNP) variants and clinical phenotypes derived from Vanderbilt's EMR-based BioVU resource [16]. We found that, taken alone, phenotype association data did not yield associations statistically different from chance. To identify biologically-relevant genetic associations, we analyzed 711 nsSNPs in genes with *in vivo* functional genetic data reported in the OMIM (On-line Mendelian Inheritance in Man) or the knock-out mouse phenotype databases, both of which catalog a partial spectrum of disease associated with loss-of-function mutations. This approach yielded 12 candidate genotype-phenotype associations, four of which we replicated in an independent data set. This approach suggests a potential for important biologic association discovery as platforms genotyping hundreds of thousands of rare nsSNPs are deployed across EMRs.

## Materials and Methods

### Ethics Statement

All data for these analyses was extracted from the Vanderbilt DNA Databank, BioVU, which accrues DNA samples extracted from leftover blood remaining from routine clinical testing. This resource has been approved as non-human subjects research by Vanderbilt's local Institutional Review Board and the federal Office of Human Research Protections (OHRP), and has been described in detail previously [16], [24]. Briefly, BioVU is linked to a de-identified Electronic Medical Record (EMR) system in which all personal identifiers have been removed, and subjects may elect to be removed from BioVU at any time. This study was also reviewed by the Vanderbilt Institutional Review Board and determined to be non-human subjects research.

### Study population

A total of 8,546 subjects who had previously been genotyped at Vanderbilt University Medical Center (VUMC) were used in the analysis. The subjects belonged to three cohorts identified from BioVU, a de-identified collection of DNA samples extracted from discarded blood and linked to de-identified EMRs [16]. Two cohorts were assembled as part of the Vanderbilt Genome Electronic medical Records (VGER) project within the electronic Medical Records and genomics (eMERGE) network [2]. The first VGER cohort (VGER-660) was comprised predominantly of EMR-defined white European ancestry subjects (N = 3,174), and the second (VGER-1M) was comprised predominantly of EMR-defined black African American subjects (n = 1,558). These cohorts were selected for genotyping using phenotype selection algorithms that identified individuals with normal cardiac conduction or type 2 diabetes (and their controls) [5], [25]. Subjects in the third cohort were selected from BioVU by an ongoing study (Vanderbilt Electronic Systems for Pharmacogenomic Assessment; VESPA) examining the genomics of drug response [26] (n = 3,940; Table S1). The largest VESPA studies are examining antibiotic responsiveness (n = 2,476 subjects) and transplant patients (n = 921 subjects). Race assignment was determined using STRUCTURE [27]: European ancestry (EA) was defined as subjects with a >90% probability of being in the CEU cluster, and African ancestry (AA) was defined as subjects with a >90% of being in the YRI cluster, using HapMap populations as references.

### SNP selection

Genotype data were acquired on one of three genotyping platforms: the Illumina Human660W-Quadv1_A genotyping platform (VGER-660), the Illumina Human1M-Duo (VGER-1M), or the Illumina Omni1_QUAD (VESPA). Each dataset was separately cleaned using the quality control pipeline developed by the eMERGE Genomics Working Group [28]. This entailed identifying gender mismatches, identifying SNPs failing concordance with HapMap, batch effects, and identification of duplicate and related individuals. After quality control analyses, the data sets were merged. The merged data set contained genotype information on 1,545,817 SNPs present on one or more of the genotyping platforms.

**Figure 1:**
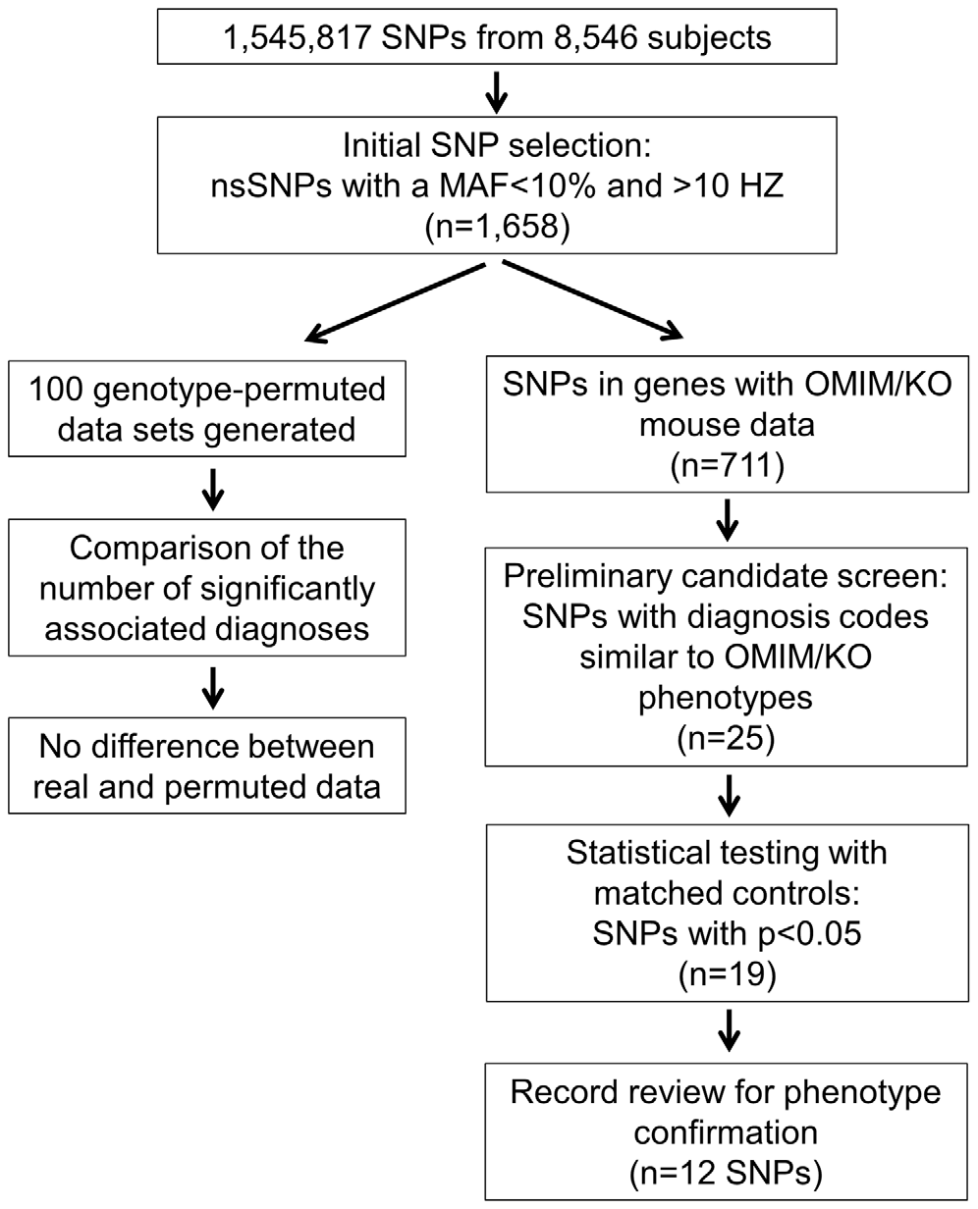
Overview of the nsSNP selection process. There was no difference in number of diagnoses significantly associated with the 1,658 nsSNPs when compared to genotype-permuted data. Hence, a nsSNP selection strategy that compared to diagnoses to those reported in either OMIM or the KO Mouse data was used. A multi-step selection and review process identified 12 candidate nsSNPs.

An overview of the SNP selection process is shown in Figure 1. Non-synonymous SNPs (nsSNPs) that had a MAF less than 10% in both EA and AA populations and had more than 10 minor allele homozygotes were selected for analysis. nsSNPs with less than 10 minor allele homozygotes were excluded to reduce statistical biases associated with very small sample sizes. A total of 1,658 nsSNPs met these initial inclusion criteria. The mean MAF was 5.3%±.3.1% (SD) and 4.7%±3.2 (SD) for EAs and AAs, respectively. The median number of subjects with genotype data available for a given nsSNP was 4,750±2,097 (SD). Of the 1,658 nsSNPs initially identified, 440 were located in genes with disease associations in the OMIM database, 555 were in the KO mouse data set. In total, 711 nsSNPs were located in 591 genes found in either the OMIM or the KO mouse data set and 284 nsSNPs were in both.

### Clinical data extraction

Clinical diagnoses, symptoms and problems for each subject were extracted from the Vanderbilt University Medical Center (VUMC) Synthetic Derivative, a de-identified image of the Vanderbilt EMR [16]. Diagnoses were derived from ICD-9 and physician-maintained problem lists. Problem lists were manually reviewed to correct misspellings and expand abbreviations and diagnoses were then mapped to their corresponding ICD-9 code using text matching. There were 13 instances where a new clinical code was created (e.g. AV nodal re-entry tachycardia) in order to capture the diagnosis with specificity (these codes can be found in Table S2). Cancer diagnoses were not included in these analyses as the molecular phenotypes described in the Mouse Phenotype database could not be easily mapped to a specific cancer type. After extraction and mapping of problem list entries, there were 8,275 unique clinical codes. In this study, we did not use the predefined list of PheWAS phenotypes but created a new one, as doing so allowed the most appropriate mapping of diagnoses experienced in the individuals [8]. *De novo* creation of aggregations based on those phenotypes in patients with rare nsSNPs theoretically enhanced our sensitivity to create potential unforeseen aggregations with rare nsSNPs that may not be found in the *a priori* PheWAS codes. These were aggregated into 1,609 groups of related codes (see Table S2 for ICD-9 groupings).

### Identifying Candidate Associations

In order to identify genotype-phenotype associations, we generated a list of all diagnoses present in two or more of the homozygotes for the minor allele for each nsSNP. Any problem that appeared on more than 5% of these lists across all nsSNPs was excluded, as this was typically caused by rarely used diagnosis codes for which just 1 or 2 cases present among the minor allele homozygotes would give a strong association p-value. For each common diagnosis, a two-sided Fisher's exact test was used to compare the proportions of affected minor allele homozygotes to affected common allele homozygotes. The heterozygotes were not used in the analysis to simplify the analysis and prevent a loss of power associated with model misclassification if the wrong association model was chosen (e.g., additive instead of recessive or dominant). A composite list of all diagnoses with an *a priori* Fisher's exact p-value less than 0.05 was then generated for each nsSNP.

To estimate the number of significant nsSNP-phenotype associations expected by chance, permutation testing was employed. We generated 100 randomized data sets by taking the 1,658 nsSNPs and permuting the link between the genotypes and phenotypes (i.e. the genotype values for a nsSNP were randomly redistributed across all patients while keeping their phenotypes intact). We then compared the number nsSNPs having diagnoses with a Bonferroni-corrected Fisher's exact p-value<0.05 using the actual genotype data to the numbers of significant diagnoses associated with each of 100 randomized data sets. We also compared the average number of diagnoses associated with an nsSNP with a p<0.05.

Based on the permutation analyses, we found that real and randomized genetic data could not be distinguished on the basis of statistical outliers. Hence, we restricted all subsequent analyses to the 711 nsSNPs in the OMIM or the KO mouse data sets. These 711 nsSNPs first underwent a human review comparing the phenotypes from the KO mouse and OMIM databases to the composite diagnosis list from the minor allele homozygotes. nsSNPs that were associated with diseases arising from a pathophysiological mechanism and organ system distribution that was comparable to the known function of the gene were selected for further review. nsSNPs were also included if the disease mechanism in the homozygotes appeared to be opposite of that described, as this could occur if an nsSNP was associated with a gain-of-function mutation. For 686 of the 711 nsSNPs, the candidate associations were deemed inconsistent with the KO mouse and OMIM databases. In all, 25 nsSNPs were selected for further evaluation.

### Association testing using matched controls and EMR validation

To more rigorously test each of the 25 nsSNPs identified above, we developed a clinical phenotype definition using composites of diagnosis codes that best approximated the phenotype descriptions in the OMIM and KO mouse databases (Table S3). For example, the *PTAFR* gene is associated with infection susceptibility including streptococcal infections [29], [30]. Hence, phenotypes comprised of ICD-9 codes for streptococcal-associated diseases including respiratory infections, streptococcal infections, sepsis, sinusitis and meningitis were defined. A significant association with at least one of these phenotypes was required in order for the nsSNP to be considered to be associated with the phenotype. In instances where numerous possible clinical presentations were possible based the phenotype description of the KO mouse, the phenotype was defined to incorporate the diagnoses observed during the initial nsSNP review. For example, *CLEC1B* was associated with abnormal blood vessel morphology in mice. The initial case review identified elevated rates of intracranial hemorrhage among the minor allele homozygotes for an nsSNP in this gene. Hence, this phenotype was specifically evaluated.

Univariate exact logistic regression comparing minor allele homozygotes to common allele homozygotes was used to test associations. The common allele homozygotes were a random sample individually matched to the minor allele homozygotes on age strata (0-4 years, 5-19 years, 20–44 years, 45–60 years and 60+ years) gender, race and data set. Binomial power calculations assuming P(disease in cases) = 30%, P(disease in controls) = 10%, number of cases = 25, alpha = 0.05 and beta = 0.80 showed that 800 controls were needed per nsSNP. Depending upon the availability of matched controls, between 800 and 1,800 matched controls were selected per nsSNP. All nsSNPs that failed to show a statistical association (defined as a p-value less than 0.05) with at least one phenotype were not considered for further review. Of the 25 nsSNPs, 19 had significant associations.

After statistical testing, the electronic records of the minor allele homozygotes for the 19 nsSNPs were reviewed by a clinician to confirm that their clinical records supported their diagnoses inferred from the ICD-9 codes and problem lists. This review was used to ascertain whether any conditions comprising one of the phenotype definitions may have been previously ruled out or may have a known etiology that would preclude an underlying genetic explanation. For instance, an ICD-9 code for joint pain in a patient for which a clinical record review indicated that the patient had an ankle fracture would not be considered a possible manifestation of gout. If clinical record review indicated that the ICD-9 codes did not support a diagnosis related to the function of the nsSNP, the nsSNP was excluded from further analysis, resulting in exclusion of 7 of the 19 SNPs.

### Replication analyses

Of the 12 candidate nsSNPs, 10 were available in an EMR-derived replication cohort that underwent genotyping using the Illumina Infinium Exome BeadChip. The replication set contained 19,599 EAs and 1,993 AAs over the ages of 30 years old who were genotyped as part of broad-based genotyping initiative at Vanderbilt. Quality control procedures for the Exome chip data have been previously described [31]. In brief, quality control was performed by VANGARD (Vanderbilt Technologies for Advanced Genomics Analysis and Research Design) and samples were analyzed in conjunction with over 32,000 other BeadChip samples. After clustering, samples were then evaluated for heterozygous consistency rate between duplicated samples and HAPMAP samples, gender mismatches, Mendelian errors, duplicate identification and exclusion of subjects more closely related than half-siblings. Data were filtered for a sample and genotype call rate>99% and deviation for Hardy Weinberg equilibrium (p>0.001). Phenotype data was based strictly on ICD-9 codes with cases defined as subjects with 1 or more codes and controls defined as those subjects with no related codes. Only those phenotypes with an association p-value<0.05 in the original analyses and with >50 cases in the replication set were evaluated. EAs and AAs were analyzed separately using an additive multivariable genetic model adjusting for age, gender and 3 principal components. A replication p-value<0.05 was considered statistically significant.

### Data analysis and external data sources

All quality control analyses of nsSNP genotyping data were performed using PLINK v1.07 [32]. Principal components were fit using EIGENSTRAT [33]. All post quality-control statistical analyses were performed using SAS v9.3 (SAS Institute, Cary, NC). Gene-disease associations were downloaded from OMIM (http://omim.org/). Phenotype information for knock-out (KO) mouse models was downloaded from the mouse genome informatics resource (http://www.informatics.jax.org). These data sources were current as of 6/24/2012.

## Results

### Permutation analyses

8,546 subjects who had previously undergone SNP genotyping were used in this study (Table 1). Approximately 70% of the study population was EAs. The mean age of their most recent clinical encounter was 52 years and an average of 7 years of clinical data was available for each subject. Two approaches to identifying candidate genotype-phenotype associations were used, as outlined in Figure 1. For the first approach, a preliminary review of phenotypes that were associated with the minor allele homozygotes for the 1,658 low MAF nsSNPs identified a number of compelling patterns of disease associations. For instance, the associations between the nsSNP (rs33947968) in the *Myo3A* gene encompassed a clinical disease spectrum that would suggest that this nsSNP contributes to cardiopulmonary disease (Table S4). However, similarly compelling phenotypic patterns were seen in reviews of associations derived from genotype-randomized data, suggesting that these associations were likely due to chance. Consistent with this notion, the number of the 1,658 nsSNPs with clinical associations with a Bonferroni-adjusted p<0.05 was similar between the real (n = 188 nsSNPs) and 100 genotype-randomized data sets (median n = 194, inter-quartile range = 184-204), as was the average number of diagnoses associated with a nsSNP with an unadjusted p<0.05 (n = 19.2 for real data vs. a median of 20.0 [IQR 19.8–20.1] for permuted sets). In addition, a role for *Myo3A* in cardiopulmonary disease is not consistent with its known biology, as expression of this gene is restricted to the ear and known mutations cause deafness [34]. Based on these results, we concluded that a completely agnostic approach to candidate nsSNP identification would result in a very high likelihood of biologically-implausible, false positive associations.

**Table 1:** Population characteristics.

Total Subjects (n)	8645
	
No. males (%)	4079 (47.2)
No. females (%)	4566 (52.8)
No. European Ancestry (%)	6002 (69.4)
No. African American (%)	1734 (20.1)
No. other races (%)	909 (10.5)
Mean (std) age of last available diagnosis (years)	52 (18)
Mean (std) duration of EMR follow-up (years)	7 (5)

### SNP-phenotype associations using KO mouse and OMIM data

In order to identify biologically-plausible gene-phenotype associations, we restricted subsequent analyses to 711 of the 1,658 nsSNPs located in genes with functions described in the OMIM or KO mouse data sets. Of these 711 nsSNPs, the minor allele homozygotes for 25 had diagnosis codes (with an association p<0.05) consistent with the known function of the gene containing the SNP. Six of these nsSNP-phenotype clusters were excluded because the genotypes were not significantly associated (p>0.05) with disease in analyses using matched controls. The medical records for each minor allele homozygote for the remaining 19 nsSNPs were reviewed to confirm that their clinical data supported their coded data. Seven of the 19 nsSNPs were excluded after this review because the clinical records suggested a disease etiology that was not consistent with the known physiology of the gene. For example, while there was a statistically significant increase in chest pain among homozygotes for an nsSNP in *DNAH5*, a gene associated with respiratory ciliary disorders and bronchiectasis, the chest pain was generally attributed to external/traumatic causes rather than intrinsic lung disease. (See Table S5 and Table S6 for details of the 13 nsSNPs excluded in these steps).

Of the twelve nsSNPs that advanced through all steps of the selection process, the mean MAF was 6.3% and 5.0% in EAs and AA, respectively, and the mean number of homozygotes for each nsSNP was 36 (Table 2). Two nsSNPs (*ERCC4* and *PLCG2*) were predicted to be damaging by PolyPhen-2 [35] analysis and one encoded a nonsense mutation (*TAAR1*). The phenotypes for 1 and 5 of the 12 nsSNPs were described only in the OMIM or KO mouse databases, respectively, and the other 6 were described in both databases (Table S3). Results of association testing with matched controls are shown in Table 3 and the problem lists for these SNPs are shown in Table S7.

**Table 2:** Characteristics of the selected nsSNPs.

SNP	Gene	Chr	Position	OMIM/KO Mouse phenotype(s)	MAF white/black	SNP function	PolyPhen prediction
rs17255978	*ADAM22*	7	87754915	Peripheral neuropathy	0.04/0.10	missense	benign
rs33986943	*AOC3*	17	41004637	Abnormal leukocyte adhesion; decreased lymphocytes in Peyer patches; decreased reduced serum IgA	0.10/0.02	missense	benign
rs16027	*CACNA1A*	19	13397560	Migraine, familial hemiplegic	0.09/0.03	missense	unknown
rs3735972	*CNGB3*	8	87588198	Macular degeneration/Achromatopsia	0.09/0.08	missense	unknown
rs1800067	*ERCC4*	16	14029033	Xeroderma Pigmentosum, XFE progeroid syndrome	0.08/0.01	missense	probably damaging
rs6031	*F5*	1	169511903	Factor V deficiency	0.00/0.08	missense	unknown
rs2291628	*FBN2*	5	127609633	Syn/polydactyly, osteoporosis, abnormal bone remodeling	0.08/0.08	missense	benign
rs13157270	*GPR98*	5	90012379	Febrile seizures, familial	0.09/0.02	missense	benign
rs16844401	*HGFAC*	4	3449652	Impaired intestinal mucosal healing	0.07/0.03	missense	benign
rs17537869	*PLCG2*	16	81922813	Familial cold auto-inflammatory syndrome	0.07/0.01	missense	probably damaging
rs5939	*PTAFR*	1	28476520	Decreased infection susceptibility, including streptococcus	0.00/0.08	missense	unknown
rs8192619	*TAAR1*	6	132966348	Increased NE/dopamine, abnormal prepulse inhibition	0.05/0.06	stop-gained	unknown

OMIM/KO mouse phenotypes are associated at the gene level, not the specific nsSNP. Minor allele frequencies (MAF) are based on the frequencies observed in this study population. Chromosome and position are from Human Annotation Release 104.

**Table 3:** Association statistics for the 12 candidate nsSNPs.

SNP/Gene	Phenotypes	Total minor allele HZ	Affected minor allele HZ	Total common allele HZ	Affected common allele HZ	OR	95% CI	p-value
rs17255978/*ADAM22*	Peripheral neuropathy	28	9	1402	143	4.2	(1.9–9.4)	0.0006
	Demyelination disease	28	0	1402	7	n/a		
	Seizures	28	1	1402	55	0.9	(0.1–6.8)	0.92
rs33986943/*AOC3*	Gram negative sepsis	35	7	1023	56	4.3	(1.8–10.3)	0.001
	All sepsis	35	16	1023	307	2.0	(1.0–3.9)	0.051
	Gram positive sepsis	35	11	1023	190	2.0	(1.0–4.2)	0.06
	Decrease serum IgA	35	0	1023	4	n/a		
rs16027/*CACNA1A*	Migraine	54	10	1512	114	2.8	(1.4–5.7)	0.004
	Convulsions	54	10	1512	128	2.5	(1.2–5.0)	0.01
	Seizures	54	4	1512	69	1.7	(0.6–4.8)	0.33
rs3735972/*CNGB3*	Cataract	74	18	1554	162	2.6	(1.5–4.6)	0.0007
	Cataract (age>50)	43	15	860	124	3.2	(1.7–6.1)	0.0005
	Macular degeneration	74	4	1554	19	4.4	(1.5–13.3)	0.008
	Colorblindness	74	0	1554	0	n/a		
	Retinopathy (not hypertension or diabetes)	74	7	1554	76	1.9	(0.9–4.3)	0.11
rs1800067/*ERCC4*	Seborrheic keratosis	42	8	1512	124	2.6	(1.2–5.8)	0.016
rs6031/*F5*	Pregnancy loss	13	3	715	23	9.0	(2.3–35.0)	0.001
	On anti-coagulant	15	4	825	74	3.7	(1.1–11.9)	0.028
	Stroke	15	4	825	81	3.3	(1.0–10.7)	0.04
	Venous thrombosis	15	3	825	52	3.7	(1.0–13.6)	0.047
	Budd-Chiari syndrome	15	0	825	1	n/a		
rs2291628/*FBN2*	Avascular necrosis	62	7	1488	29	6.4	(2.7–15.3)	<.0001
	Osteomyelitis	62	8	1488	76	2.8	(1.3–6.0)	0.01
	Bone fracture	62	20	1488	306	1.8	(1.1–3.2)	0.029
	Pathologic fracture	62	5	1488	43	2.9	(1.1–7.7)	0.027
	Osteoporosis	62	7	1488	206	0.8	(0.4–1.8)	0.56
	Joint disease	62	27	1488	663	1.0	(0.6–1.6)	0.87
	Polydactyly	62	0	1488	5	n/a		
rs13157270/*GPR98*	Epilepsy	48	7	1488	77	3.1	(1.4–7.2)	0.007
	Febrile seizure	48	0	1488	4			
	Convulsions	48	7	1488	140	1.6	(0.7–3.7)	0.23
								
rs16844401/*HGFAC*	GI bleed	32	6	1503	68	4.9	(1.9–12.2)	0.0007
	GI infections (bacterial)	32	1	1503	109	0.4	(0.1–3.1)	0.38
rs17537869/*PLCG2*	Extrinsic asthma	11	2	1279	18	15.6	(3.1–77.2)	0.0008
	Humoral immunity/Decreased IgA,IgM	11	1	1279	0	9.7	(1.2–81.7)	0.03
	Allergic reactions	11	5	1279	251	3.4	(1.0–11.3)	0.04
	Allergic rhinitis	11	2	1279	182	1.3	(0.3–6.3)	0.70
	Cold induced urticaria	11	0	1279	1	n/a		
rs5939/*PTAFR*	Bacterial meningitis	17	3	964	7	29.3	(6.9–125.1)	<.0001
	Acute upper respiratory infection	17	11	964	199	7.0	(2.6–19.3)	0.0001
	Chronic sinusitis	17	6	964	103	4.6	(1.7–12.6)	0.003
	Sinusitis	17	8	964	188	3.7	(1.4–9.6)	0.008
	Acute sinusitis	17	6	964	137	3.3	(1.2–9.1)	0.02
	All sepsis	17	3	964	84	2.2	(0.6–8.0)	0.21
	Gram positive sepsis	17	2	964	53	2.3	(0.5–10.3)	0.27
	Strep infections	17	1	964	41	1.4	(0.2–10.9)	0.74
	Gram negative sepsis	17	0	964	12			
rs8192619/*TAAR1*	Anxiety	17	7	725	132	4.6	(1.7–12.5)	0.002
	Depression	17	9	725	173	3.6	(1.4–9.4)	0.009
	Schizophrenia	17	0	725	12	n/a		

For each nsSNP, clinical phenotypes were constructed using diagnosis codes that closely approximated the phenotype descriptions in the OMIM and KO mouse databases. Shown are the subject counts and results of exact logistic regression analyses comparing minor allele homozygotes to matched common allele homozygotes. The common allele homozygotes were matched for age, race, gender and data set.

### Replication analyses

The significant associations for 10 of the 12 nsSNPs were evaluated using an additive genetic model in an independent data set. Replicated associations were observed for 4 of the 10 genes (Table 4): *CNGB3* (macular degeneration in EAs, OR = 1.2 [1.0–1.4], p = 0.03), *F5* (stroke in AAs, OR = 1.4 [1.0–1.9], p = 0.04), *GPR98* (convulsions in AAs, OR = 1.9 [1.1–3.3], p = 0.02) and *HGFAC* (GI bleeding in EAs, OR = 1.2 [1.0–1.4], p = 0.02). The association of GI bleeding with *HGFAC* [36] in humans has not been described.

**Table 4:** Replication analyses.

		European Americans					African Americans			
SNP/Gene	Phenotypes	Cases/Controls	OR	95% CI	p-value		Cases/Controls	OR	95% CI	p-value
rs17255978/	Peripheral neuropathy	2284/17315	1.0	(0.9–1.2)	0.83		264/1729	1.2	(0.9–1.6)	0.33
*ADAM22*										
rs33986943/	Gram negative sepsis	—	—	—	—		—	—	—	—
*AOC3*	All sepsis	12038/7561	1.0	(0.9–1.1)	0.62		1456/537	1.0	(0.6–1.6)	0.89
rs3735972/	Cataract	2879/16720	1.1	(1.0–1.2)	**0.04**		418/1575	0.9	(0.6–1.2)	0.34
*CNGB3*	Macular degeneration	809/18790	1.2	(1.0–1.4)	**0.03**		56/1937	1.1	(0.5–2.2)	0.84
rs1800067/	Seborrheic keratosis	2952/16647	1.0	(0.9–1.2)	0.5		—	—	—	—
*ERCC4*										
rs6031/	Pregnancy loss	—	—	—	—		—	—	—	—
*F5*	On anti-coagulant	—	—	—	—		—	—	—	—
	Stroke	—	—	—	—		255/1738	1.4	(1.0–1.9)	**0.04**
	Venous thrombosis	—	—	—	—		181/1812	0.8	(0.5–1.2)	0.32
rs2291628/	Avascular necrosis	147/19452	1.0	(0.6–1.5)	0.82		—	—	—	—
*FBN2*	Osteomyelitis	309/19290	1.0	(0.8–1.4)	0.75		60/1933	1.2	(0.6–2.3)	0.61
	Bone fracture	2706/16893	1.0	(0.9–1.1)	0.63		294/1699	0.8	(0.6–1.2)	0.29
	Pathologic fracture	525/19074	1.1	(0.8–1.3)	0.64		—	—	—	—
rs13157270/	Epilepsy	659/18940	1.0	(0.8–1.2)	0.91		83/1910	2.0	(1.0–4.2)	0.06
*GPR98*	Convulsions	1215/18384	1.0	(0.8–1.1)	0.53		164/1829	1.9	(1.1–3.3)	**0.02**
rs16844401/	GI bleed	1630/17969	1.2	(1.0–1.4)	**0.02**		251/1742	1.0	(0.5–1.9)	0.97
*HGFAC*										
rs17537869/	Extrinsic asthma	360/19239	1.2	(0.9–1.6)	0.12		73/1920	0.0	—	0.99
*PLCG2*	Humoral immunity/Decreased IgA,IgM	75/19524	1.1	(0.6–2.0)	0.81		—	—	—	—
	Allergic reactions	3720/15879	0.9	(0.8–1.0)	0.11		355/1638	1.2	(0.5–2.8)	0.68
rs5939/	Bacterial meningitis	—	—	—	—		—	—	—	—
*PTAFR*	Acute upper respiratory infection	—	—	—	—		466/1527	1.2	(0.9–1.6)	0.15
	Chronic sinusitis	—	—	—	—		—	—	—	—
	Sinusitis	—	—	—	—		—	—	—	—
	Acute sinusitis	—	—	—	—		—	—	—	—

Replication analyses for nsSNP-phenotype associations using an additive logistic regression model adjusting for age, gender and principal components. A (—) indicates that less than 50 cases (i.e., individuals with the given phenotype) were available for analyses.

## Discussion

In the present study, we evaluated the feasibility of identifying gene-phenotype associations using low MAF nsSNPs in conjunction with data extracted from the VUMC BioVU resource, an integrated collection of genotype and EMR data. We found that an agnostic approach based strictly on statistical outliers identified a number of nsSNPs with clinically interesting patterns of disease associations, but permutation analyses suggested that these associations were likely due to chance. To circumvent this problem, we used *in vivo* functional genomic data to identify clinically-relevant candidate gene-phenotype associations. Our approach incorporated a clinical/biological review process that identified biologically plausible candidate phenotypes associated with 12 nsSNPs. Of the 10 candidates nsSNPs evaluated in replication analyses, 4 nsSNPs had significant associations: *CNGB3* (macular degeneration in EAs), *F5* (stroke in AAs), *GPR98* (convulsions in AAs) and *HGFAC* (GI bleeding in EAs).

We restricted our analyses to minor allele homozygotes, as these subjects would be expected to manifest the deleterious effects of a nsSNP variant if the mode of genetic action is either additive or recessive [37]. We tested the hypothesis that a review of clinical codes shared among individuals homozygous for a nsSNP by an expert clinician would identify clinical disease patterns that would suggest a common predisposing genetic lesion. When the clinical review was conducted without *a priori* knowledge of the function of the gene, we observed that there were a number of false positive leads, which were due to the fact that a number of clinical codes often co-occur within a patient and, thus, can create a constellation of associations that would suggest that the homozygous carriers had a functional genetic lesion. For instance, patients with a cardiac valvular disorder may also have a number of specific and non-specific related cardiac codes such as “Cardiac complications”, “Heart failure” and “Cardiac dysrhythmias”. Hence, these codes may cluster, giving the impression that it is associated with a heavy burden of cardiac disease. To mitigate these false positive associations, the clinical review was conducted with knowledge about the *in vivo* function of the gene, as reported in the OMIM or KO mouse data sources. While one strength of this approach was the identification of candidate nsSNPs with strong biological plausibility, using the data described in the KO mouse and OMIM resources presented challenges as many cataloged mutations cause complete loss-of-function associated with extreme, multi-organ phenotypes that are not easily translated into plausible clinical manifestations. Furthermore, in KO mice, many of the mutations were associated with embryonic lethality, or the phenotypic characterization was restricted to early embryonic anomalies [38]. Many phenotypes were also characterized at the molecular or cellular level, which posed similar translational challenges. The EMR data was also restricted to binary disease data, which prevented us from analyzing previously-reported quantitative phenotypes (e.g. LDL levels) known to be affected by some of these genes. While we observed some instances where the homozygotes had a set of coded symptoms that might be expected based on the function of the gene, a further review of the clinical records demonstrated that these symptoms were attributable to causes unrelated to the function of the gene. Overall, these challenges severely limited the utility of this general approach. Indeed, only four replicable associations were identified among 711 nsSNPs evaluated, resulting in only a 0.5% success rate.

The clinical review was also used in an effort to detect genetic pleiotropy. In particular, we were interested in identifying nsSNP variants that perturb broad underlying physiological mechanisms. Such variants would be expected to distribute their effects across a broad clinical spectrum, resulting in multiple weak statistical associations with a number of mechanistically related phenotypes. Hence, our lists of diagnoses evaluated included those that occurred at rates modestly higher than would be expected by chance (i.e. those with p<0.05) in order to increase our sensitivity for detecting pleiotropy. An example of a pleiotropic nsSNP that we identified was in the *F5* gene which encodes a clotting factor known to be associated with thrombosis [39], [40] and was associated with modestly elevated rates of spontaneous abortions, DVTs and strokes. Interestingly, the nsSNP in *F5* that we observed (rs6031) is not the well-characterized *F5* Leiden mutation found among EA subjects. This nsSNP (rs6031) was predominantly found in AAs, none of which carried the *F5* Leiden mutation.

We selected nsSNPs with MAFs below 10% in both EA and AA subjects. We hypothesized that nsSNPs maintained at low frequencies across both ancestries were more likely to be located within regions under negative evolutionary selection pressure and could be associated with relatively strong genotype/phenotype associations. Our data, however, are not consistent with this hypothesis, as the replicable associations that we observed, such as convulsions and an nsSNP in *GPR98*, were typically seen within a single racial group. Our hypothesis would have suggested that the associations would be persistent across races. Hence, it is more likely that the SNPs had low frequencies across races due to factors other than selection pressure. As an alternative approach to SNP selection, we could have selected SNPs which were predicted to be damaging using predictive software [41], [42], which may have given a higher proportion of significant and replicable of SNP associations.

Of the four genes that we identified that had replicable phenotypic associations, three, including *F5* described above, have been previously reported. Variants in *CNGB3* have been associated with achromatoplasia and juvenile macular degeneration [43]–[45]. *GPR98* has been associated with febrile seizures in humans and knock-out mice develop audiogenic seizures [46]–[48]. *HGFAC* (hepatocyte growth factor activator) encodes a proteolytic enzyme that cleaves and activates hepatocyte growth factor [49]. Mice deficient in this gene demonstrate a decreased capacity to repair injured intestinal epithelium^33^. We observed that an nsSNP variant in this gene was associated with a clinical code for GI bleeding, suggesting that this variant may be impairing endothelial repair mechanisms.

A benefit of using EMR-derived data for this type of genetic analysis is that the study population may either carry a high risk genetic background or have experienced environmental challenges that allow a phenotype to be expressed. For instance, the *HGFAC* knock-out mouse did not have an observable GI endothelial phenotype until challenged with a caustic agent [36]. Similarly, patients may seek healthcare at a tertiary care center such as VUMC because they had the requisite exposures to unmask the phenotype. Hence, an EMR-based study population may be enriched in extreme phenotypes.

While EMR data is a rich resource for hypothesis generation and testing, there are challenges to its use in this type of analysis. As compared to targeted epidemiological studies or clinical trials, phenotypes entered into the EMR are often not concisely defined and the degree and extent of clinical ascertainment are variably affected by the reason a patient is seeking clinical care. For instance, a patient whose only records available are those from a particular clinical specialty may have limited information pertaining to diseases outside of that specialty. The direction of this bias would tend to underestimate prevalence rates. This bias is compounded by the fact that not all of the data captured in an EMR is amenable to extraction using coded data, and others may require more advanced methods, such as natural language processing [50], which often require modifications to solve particular problems. For instance, a record review of the *F5* mutation homozygotes revealed that 5 of the 13 (38%) women had a history of spontaneous abortions. Only 3 of 13 (23%) were identified using ICD-9 codes and problem lists. It is also difficult to gauge the clinical severity of a problem strictly from easily-extractable coded data. This limitation tends to lead to non-differential misclassification and attenuates statistical associations. The data sets that we analyzed were not expressly curated for the phenotypes that were evaluated. Hence, the differential disease compositions of the data sets could account for our low replication rates. For instance, there is human and mouse data supporting a role for *PTAFR* gene variants and susceptibility to invasive streptococcal infections [29], [30]. While a nsSNP in this gene was associated with infections consistent with streptococcus in our initial data evaluation, these associations were not replicated. This could be due to a different pattern of infections between the data sets. Alternatively, the initial analysis was based on a comparison of homozygotes, and thereby did not assume a specific mode of genetic inheritance. While this association was not replicated using an additive model, when we used a recessive genetic model, we found that the *PTAFR* variant was associated with acute sinusitis infection and upper respiratory infections (data not shown), suggesting that it may be acting through a recessive mode of action.

A final limitation of this study was the relatively small sample size of the study population, which limited the power to detect associations, especially when evaluating low frequency variants. This limitation was likely an important reason as to why a purely statistical approach to identifying genotype-phenotype associations did not perform better than chance. Hence, a large sample size would likely have allowed us to identify a reduced set of genotype-phenotype associations using only statistical criteria. This has been the true with pheWAS approach, in general, which has shown robust phenotype replication and discovery when studies are adequately powered [9], [11].

In summary, we explored an intensive, clinically-oriented approach to identify biologically-plausible gene-phenotype associations using an EMR linked to genetic data. As EMR data resources mature and genotyping data continues to become increasingly available, approaches such as ours may facilitate the identification of the specific genetic underpinnings of numerous clinical conditions. Our analyses also demonstrate the large potential for identifying compelling, but likely spurious associations that arise when working with high-dimensional, correlated phenotypic data sets. Hence, future approaches that integrate biological data into the discovery process will be critical to identify valid and clinically meaningful gene-disease associations.

## Supporting Information

Table S1 **Subcohorts in the VESPA study.**
(DOCX)

Table S2 **ICD-9 groupings.**
(DOCX)

Table S3 **Phenotype definitions.**
(DOCX)

Table S4 **Diagnoses associated with SNP rs33947968 in the** ***Myo3A*** **gene.**
(DOCX)

Table S5 **Association testing results for SNPs excluded during the review process.**
(DOCX)

Table S6 **SNPs excluded based on record review.**
(DOCX)

Table S7 **Shared diagnosis lists for the 12 selected SNPs.**
(DOCX)
