# Supplementary material for: Integrating EMR-Linked and In Vivo Functional Genetic Data to Identify New Genotype-Phenotype Associations
Source: PLoS One. 2014 Jun 20;9(6):e100322. doi: 10.1371/journal.pone.0100322 (PMC4065041; doi:10.1371/journal.pone.0100322)
Supplement: Table S1 — Subcohorts in the VESPA study. (DOCX) [file pone.0100322.s001.docx]

**Supplemental table 1. Subcohorts in the VESPA study**. Shown are groupings and subject counts for the largest substudies comprising the VESPA cohort. The “other” category is comprised of subjects participating in studies of cardiac early repolarization, carotid artery stenting, heparin-induced thrombocytopenia and statin-induced myopathy. The groupings shown here were used for selection of matched controls.

| **Vespa subcohort** | **Number of subjects** |
| --- | --- |
| Heart transplant | 129 |
| Kidney transplant | 792 |
| C. Difficile cases and controls | 1681 |
| Vancomycin response | 795 |
| Other | 543 |
