# Supplementary material for: Integrating EMR-Linked and In Vivo Functional Genetic Data to Identify New Genotype-Phenotype Associations
Source: PLoS One. 2014 Jun 20;9(6):e100322. doi: 10.1371/journal.pone.0100322 (PMC4065041; doi:10.1371/journal.pone.0100322)
Supplement: Table S2 — ICD-9 groupings. (DOCX) [file pone.0100322.s002.docx]

**Supplemental table 2. ICD-9 groupings.** Each row contains a problem heading and the ICD-9 codes mapped to that heading. These ICD-9 groupings were used to generate the problem lists used during the initial clinical data review.

| **Problem** | **ICD-9 codes** |
| --- | --- |
| Diseases of the urinary system-Kidney | 580.8 581.8 582.8 583.8 593.8 |
| Acute poststreptococcal glomerulonephritis | 580.0 |
| Acute glomerulonephritis with lesion of rapidly progressive glomerulonephritis | 580.4 582.4 |
| Acute glomerulonephritis in diseases classified elsewhere | 580.81 |
| Interstitial nephritis | 580.89 583.89 |
| Acute glomerulonephritis with unspecified pathological lesion in kidney | 580.9 |
| Nephrotic syndrome | 581. 581.9 |
| Proliferative glomerulonephritis | 581.0 582.0 583.0 |
| Membranous glomerulonephritis | 581.1 583.1 |
| Nephritis; nephrosis; renal sclerosis | 581.2 583.2 |
| Nephrotic syndrome with lesion of minimal change glomerulonephritis | 581.3 |
| Nephrotic syndrome in diseases classified elsewhere | 581.81 |
| Chronic glomerulonephritis in diseases classified elsewhere | 582.81 |
| Sclerosing glomerulonephritis | 582.1 |
| Alport Syndrome | alport.1 |
| Rapidly progressive glomerulonephritis | 583.4 |
| Glomerulonephritis/Insterstitial nephritis | 580. 582. 582.2 582.89 582.9 |
| Nephropathy | 583.6 583.7 583.81 583.9 |
| Renal sclerosis NOS | 587. |
| Tubular necrosis NOS | 584.5 |
| Necrotizing renal papillitis | 584.7 |
| Acute renal failure | 584.6 584.8 584.9 |
| Uremic coma | 586. |
| Kidney replaced by transplant | V42.0 |
| Chronic renal failure-Stage I-III | 585.1 585.2 585.3 585.9 |
| Chronic renal failure | 585.4 585.5 585.6 |
| Renal dialysis | 792.5 V45.11 V45.12 V56.0 V56.1 V56.2 V56.31 V56.8 |
| Other specified types of cystitis | 595.8 |
| Infections of kidney | 590. 590.0 590.00 590.01 590.1 590.10 590.11 590.2 590.3 590.80 590.81 590.9 |
| Cystitis | 595. 595.0 595.1 595.2 595.3 595.4 595.81 595.82 595.89 595.9 |
| Urethritis | 597.0 597.8 597.80 597.81 598. 598.0 598.01 |
| Urinary tract infection, site not specified | 599.0 |
| Staghorn calculus | 592.0 |
| Calculus of ureter | 592.1 |
| Other and unspecified urinary calculus | 592.9 594. 594.0 594.1 594.2 594.8 788.0 V13.01 |
| Hydroureteronephrosis | 591. |
| Nephrogenic diabetes insipidus | 588.1 |
| Renal tubular disorder | 588.89 |
| Small kidney of unknown cause | 589. |
| Other and unspecified diseases of kidney | 588. 588.0 588.8 588.81 589.0 589.1 593.0 593.1 593.2 |
| Other and unspecified diseases of ureters | 593.3 593.4 593.5 593.6 593.7 593.70 593.71 593.72 593.73 593.81 593.82 593.89 593.9 |
| Bladder neck obstruction | 596.0 |
| Diverticulum of bladder | 596.3 |
| Other specified causes of urethral stricture | 598.8 |
| Neurogenic bladder NOS | 596.54 |
| Other and unspecified diseases of bladder and urethra | 596.1 596.2 596.4 596.5 596.51 596.52 596.53 596.55 596.59 596.6 596.7 596.89 596.9 598.1 598.2 599.1 599.2 599.3 599.4 599.5 599.81 599.83 599.84 |
| Intrinsic (urethral) sphincter deficiency [ISD] | 599.82 |
| Hematuria | 599.7 599.71 599.72 |
| Retention of urine | 788.2 788.21 788.29 |
| Methemoglobinuria | 791.2 |
| Myoglobinuria | 791.3 |
| Other and unspecified genitourinary symptoms | 599.6 599.69 599.89 599.9 788.1 788.3 788.31 788.32 788.33 788.34 788.35 788.36 788.37 788.38 788.39 788.41 788.42 788.43 788.5 788.6 788.61 788.62 788.63 788.64 788.65 788.69 788.7 788.8 788.9 788.91 788.99 791.0 791.1 791.4 791.7 791.9 793.5 794.4 V13.00 V13.02 V13.09 V41.7 V43.5 V44.5 V44.51 V44.52 V44.59 V44.6 V47.4 V47.5 V53.6 V55.5 V55.6 |
| Personl hx nephrotic syn | V13.03 |
| Other specified disorders of male genital organs | 607.8 608.8 |
| Hyperplasia of prostate | 600.0 600.00 600.01 600.10 600.11 600.20 600.21 600.3 600.90 600.91 |
| Other specified inflammatory diseases of prostate | 601. 601.8 |
| Other inflammatory disorders of male genital organs | 608.4 |
| Prostatits | 601.0 601.1 601.2 601.3 601.4 |
| Hydrocele | 603. 603.0 603.1 603.8 603.9 |
| Orchitis and epididymitis | 604. 604.0 604.9 604.90 604.91 604.99 607.1 607.2 608.0 |
| Dysplasia of prostate | 602.3 |
| Other male genital disorders-prostate | 602.0 602.1 602.2 602.8 602.9 605. |
| Infertility, male | 606. |
| Infertility | infer.1 |
| Oligospermia | 606.0 606.1 |
| Peyronie's disease | 607.85 |
| Leukoplakia of penis | 607.0 |
| Other male genital disorders | 606.8 607. 607.3 607.81 607.82 607.83 607.84 607.89 608.1 608.2 608.21 608.22 608.23 608.24 608.3 608.81 608.82 608.83 608.84 608.85 608.86 608.87 608.89 608.9 792.2 |
| Galactorrhea not associated with childbirth | 611.6 |
| Fibroadenosis of breast | 610.2 |
| Diffuse cystic mastopathy | 610.1 |
| Fibrosclerosis of breast | 610.3 |
| Nonmalignant breast conditions | 610. 610.0 610.4 610.8 611.0 611.1 611.2 611.3 611.4 611.5 611.71 611.72 611.79 611.81 611.82 611.83 611.89 611.9 612.0 612.1 793.80 793.81 793.82 793.89 |
| Pelvic peritoneal adhesions, female (postoperative) (postinfection) | 614.6 |
| Cervicitis and endocervicitis | 616. 616.0 |
| Unspecified inflammatory disease of female pelvic organs and tissues | 614.9 |
| Inflammatory diseases of uterus, except cervix | 615.0 615.1 |
| Other inflammatory diseases of female pelvic organs | 614.0 614.1 614.2 614.3 614.4 614.5 614.7 614.8 615.9 616.1 616.10 616.11 616.2 616.3 616.4 616.5 616.51 616.81 616.89 616.9 625.71 |
| Endometriosis | 617. 617.0 617.1 617.2 617.3 617.4 617.5 617.6 617.8 |
| Prolapse of female genital organs | 618.00 618.01 618.02 618.03 618.04 618.05 618.09 618.1 618.2 618.3 618.4 618.5 618.6 618.7 618.8 618.81 618.82 618.83 618.84 618.9 |
| Primary dysmenorrhea | 625.3 |
| Scanty or infrequent menstruation | 626.1 |
| Menstrual disorders | 626. 626.0 626.2 626.3 626.4 626.5 626.6 626.8 626.9 |
| Ovarian cyst | 620. 620.0 620.1 620.2 |
| Premature menopause | 256.31 |
| Menopausal disorders | 256.39 627. 627.0 627.1 627.2 627.3 627.4 627.8 V07.4 |
| Infertility, female, associated with anovulation | 628.0 |
| Infertility, female, of pituitary-hypothalamic origin | 628.1 |
| Female infertility | 628.2 628.3 628.4 628.8 |
| Infertility, female, of unspecified origin | 628.9 |
| Pap smear abnormalities | 795.00 795.01 795.02 795.09 V13.22 |
| History-pre-term labor | V13.21 |
| Female genital pain and other symptoms | 625.0 625.1 625.2 625.4 625.5 625.6 625.7 625.79 625.8 625.9 pelvpn.1 |
| Female genital disorders-fistula | 619.0 619.1 619.2 619.8 619.9 |
| Female genital disorders-ovarian | 620.3 620.4 620.5 620.8 620.9 |
| Other and unspecified female genital disorders | 620.6 621.0 621.1 621.2 621.3 621.31 621.32 621.33 621.35 621.4 621.5 621.6 621.7 621.8 621.9 622. 622.0 622.1 622.11 622.12 622.3 622.4 622.5 622.6 622.7 622.8 623. 623.0 623.1 623.2 623.3 623.4 623.5 623.6 623.7 623.8 624. 624.01 624.02 624.09 624.1 624.2 624.3 624.4 624.6 624.8 624.9 626.7 629.0 629.1 629.20 629.21 629.22 629.23 629.29 629.8 629.81 629.9 795.07 795.08 V13.2 V55.7 |
| Teratogenesis | 629.89 |
| Female genital disorders-hematoma | 620.7 624.5 |
| Pap smear cervix w LGSIL | 795.03 |
| Pap smear cervix w HGSIL | 795.04 |
| Leukoplakia of cervix (uteri) | 622.2 |
| Contraceptive and procreative management | V25.03 |
| Contraceptive and procreative management | V15.7 V25. V25.01 V25.02 V25.04 V25.09 V25.2 V25.3 V25.40 V25.41 V25.42 V25.43 V25.49 V25.5 V25.8 V26. V26.0 V26.1 V26.21 V26.29 V26.34 V26.41 V26.42 V26.49 V26.51 V26.52 V26.82 V26.89 V45.51 V45.52 V45.59 |
| Procreation management--genetic | V26.31 V26.32 V26.35 V26.39 |
| Genetic counseling | V26.33 |
| Assist repro fertility | V26.81 |
| Spontaneous abortion | 634. 634.0 634.01 634.02 634.10 634.11 634.12 634.21 634.22 634.30 634.31 634.32 634.4 634.41 634.42 634.50 634.51 634.52 634.60 634.62 634.7 634.71 634.72 634.8 634.81 634.82 634.9 634.90 634.91 634.92 |
| Induced abortion | 635.0 635.01 635.02 635.1 635.11 635.12 635.2 635.21 635.22 635.3 635.32 635.41 635.5 635.51 635.52 635.62 635.7 635.71 635.72 635.8 635.81 635.82 635.9 635.91 635.92 636.0 636.01 636.1 636.11 636.21 636.3 636.31 636.4 636.51 636.6 636.7 636.72 636.9 636.91 637. 637.0 637.00 637.01 637.1 637.11 637.12 637.2 637.21 637.3 637.41 637.42 637.5 637.51 637.70 637.71 637.72 637.8 637.81 637.82 637.9 637.91 637.92 638.0 638.1 638.2 638.3 638.7 638.8 638.9 |
| Postabortion complications | 639. 639.0 639.1 639.2 639.3 639.4 639.5 639.6 639.8 |
| Ectopic pregnancy | 633.0 633.00 633.01 633.1 633.10 633.11 633.2 633.20 633.21 633.80 633.81 633.9 633.90 633.91 |
| Placenta previa | 641.00 641.01 641.03 641.1 641.11 |
| Abruptio placenta | 641.2 641.21 641.23 |
| Other hemorrhage during pregnancy; childbirth and the puerperium | 640.0 640.00 640.01 640.03 640.80 640.81 640.83 640.90 640.91 640.93 641.13 641.3 641.30 641.31 641.8 641.80 641.81 641.9 641.90 641.91 |
| Toxemia NOS | 642.4 |
| Preeclampsia and eclampsia-mild | 642.40 642.41 642.42 642.43 |
| Mild or unspecified pre-eclampsia, postpartum | 642.44 |
| Preeclampsia and eclampsia-severe | 642.50 642.51 642.52 642.53 |
| Severe pre-eclampsia, postpartum | 642.54 |
| Eclampsia | 642.60 642.61 642.62 642.63 |
| Eclampsia, postpartum | 642.64 |
| Preeclampsia and eclampsia | 642.70 642.71 642.72 642.73 642.74 |
| Other hypertension in pregnancy-postpartum | 642.04 642.24 642.34 |
| Other hypertension in pregnancy | 642.00 642.01 642.02 642.03 642.10 642.11 642.12 642.13 642.14 642.20 642.21 642.22 642.23 |
| Other hypertension in pregnancy | 642.3 642.30 642.31 642.32 642.33 642.90 642.91 642.92 642.93 642.94 |
| Threatened premature labor | 644.00 644.03 |
| Early onset of delivery | 644.2 644.20 644.21 |
| Other early or threatened labor | 644.10 644.13 |
| Prolonged pregnancy | 645.10 645.11 645.13 645.20 645.21 645.23 |
| Diabetes or abnormal glucose tolerance complicating pregnancy-Postpart | 648.04 648.84 |
| Diabetes or abnormal glucose tolerance complicating pregnancy; childbi | 648.0 648.01 648.02 648.03 648.80 648.81 648.82 648.83 |
| Infections of genitourinary tract during pregnancy | 646.6 646.61 646.62 646.64 |
| Anemia during pregnancy | 648.2 648.21 648.22 648.23 |
| Anemia, postpartum | 648.24 |
| Missed abortion | 632. |
| Hyperemesis gravidarum | 643. 643.00 643.01 643.03 643.10 643.11 643.13 |
| Infectious and parasitic complications in mother affecting pregnancy | 647.01 647.03 647.04 647.10 647.11 647.13 647.14 647.21 647.23 647.30 647.31 647.32 647.33 647.34 647.43 647.50 647.51 647.53 647.54 647.60 647.61 647.62 647.63 647.64 647.80 647.81 647.82 647.83 647.84 647.90 647.91 647.92 647.93 647.94 |
| Hydatidiform mole | 630. |
| Complications of pregnancy-Vomiting | 643.20 643.21 643.23 643.80 643.81 643.83 643.90 643.91 643.93 |
| Papyraceous fetus | 646.00 646.01 646.03 |
| Complications of pregnancy--Adema/wt gain | 646.10 646.11 646.12 646.13 646.14 |
| Complications of pregnancy-Renal disease | 646.20 646.21 646.22 646.24 |
| Habitual aborter | 646.30 646.31 646.33 |
| Peripheral neuritis in pregnancy | 646.4 646.40 646.41 646.42 646.44 |
| Asymptomatic bacteriuria in pregnancy | 646.5 646.51 646.52 |
| Liver disorders in pregnancy | 646.70 646.71 |
| Other and unspecified complications of pregnancy | 646.8 646.81 646.82 646.84 646.90 646.91 646.93 |
| Complications of pregnancy--thryoid | 648.10 648.11 648.12 648.13 |
| Thyroid dysfunction, postpartum | 648.14 |
| Complications of pregnancy-Congenital cardiovascular disorders | 648.50 648.51 648.52 648.53 |
| Congenital cardiovascular disorders of mother, postpartum | 648.54 |
| Other cardiovascular diseases of mother | 648.60 648.61 648.62 648.63 |
| Other cardiovascular diseases of mother, postpartum | 648.64 |
| Complications of pregnancy-bone/joint/other | 648.70 648.71 648.72 648.73 648.74 648.90 648.91 648.92 |
| Other current conditions classifiable elsewhere of mother, antepartum | 648.93 |
| Other current conditions classifiable elsewhere of mother, postpartum | 648.94 |
| Complications of pregnancy-Coagulation def | 649.30 649.31 649.33 649.34 |
| Other and unspecified complications of pregnancy | 649.00 649.01 649.02 649.03 649.04 649.10 649.11 649.12 649.13 649.14 649.20 649.21 649.23 649.24 649.40 649.41 649.42 649.43 649.44 649.50 649.51 649.53 649.60 649.61 649.63 649.64 |
| Breech presentation | 652.20 652.21 652.23 |
| Other malposition; malpresentation | 652.00 652.01 652.03 652.10 652.11 652.13 652.30 652.31 652.33 652.41 652.43 652.51 652.53 652.60 652.61 652.63 652.70 652.71 652.73 652.80 652.81 652.83 652.90 652.91 652.93 660.00 660.01 660.03 |
| Fetopelvic disproportion | 653.40 653.41 653.43 |
| Other disproportion or obstruction | 653.00 653.01 653.03 653.10 653.11 653.13 653.20 653.21 653.23 653.31 653.33 |
| Other disproportion or obstruction--Fetal origin | 653.50 653.51 653.53 653.70 653.71 653.73 |
| Other disproportion or obstruction | 653.80 653.81 653.83 653.90 653.91 653.93 660.10 660.11 660.13 660.21 660.23 660.30 660.31 660.33 660.40 660.41 660.43 660.51 660.60 660.61 660.63 660.71 660.73 660.81 660.83 660.90 660.91 660.93 678.10 678.11 678.13 |
| Hydrocephalic fetus | 653.60 653.61 653.63 |
| Previous cesarean section | 654.20 654.21 654.23 |
| Fetal distress | 656.30 656.31 656.33 |
| Uterine inertia | 661.00 661.01 661.03 661.10 661.11 661.13 661.20 661.21 661.23 |
| Precipitate labor | 661.3 661.31 661.33 |
| Other abnormal forces of labor | 661.4 661.40 661.41 661.43 661.9 661.90 661.91 661.93 662. 662.00 662.01 662.03 662.10 662.11 662.13 662.20 662.21 662.23 662.31 |
| Premature rupture of membranes | 658.10 658.11 658.13 |
| Infection of amniotic cavity | 658.4 658.41 658.43 |
| Polyhydramnios | 657.0 657.00 657.01 657.03 |
| Oligohydramnios | 658.0 658.00 658.01 658.03 |
| Other problems of amniotic cavity | 658.20 658.21 658.23 658.31 658.80 658.81 658.83 658.90 658.91 658.93 779.84 792.3 |
| Other umbilical cord complications | 663.0 663.01 663.03 663.10 663.11 663.13 663.20 663.21 663.23 663.30 663.31 663.33 663.41 663.5 663.51 663.53 663.60 663.61 663.63 663.81 663.83 663.90 663.91 663.93 |
| Perineal laceration | 664.00 664.01 664.04 664.1 664.10 664.14 664.2 664.20 664.24 664.3 664.30 664.34 664.4 664.40 664.44 664.50 664.51 664.54 664.61 664.81 664.84 664.90 664.91 664.94 |
| Forceps delivery | 669.50 669.51 |
| Other complications of birth; puerperium affecting management of mothe | V23.41 V23.49 |
| Postpartum hemorrhage | 666. 666.0 666.00 666.02 666.1 666.10 666.12 666.2 666.22 |
| Postpartum hemorrhage | 666.3 666.30 666.32 |
| Puerperal infection | 670.00 670.02 670.04 670.10 670.12 670.14 670.2 670.22 670.32 670.34 670.82 670.84 |
| Complications of the puerperium-Venous | 671. 671.00 671.01 671.02 671.03 671.04 671.10 671.11 671.13 |
| Complications of the puerperium-thrombophlebitis | 671.2 671.21 671.22 671.23 671.24 |
| Complications of the puerperium-Deep thrombophlebitis | 671.3 671.31 |
| Phlegmasia alba dolens | 671.4 |
| Other antepartum phlebitis and thrombosis | 671.50 671.51 671.52 671.53 |
| Other postpartum phlebitis and thrombosis | 671.40 671.42 671.54 |
| Complications of the puerperium | 671.80 671.81 671.82 671.83 671.84 671.91 671.92 671.93 671.94 672.00 672.02 672.04 673.00 673.01 673.02 673.03 673.04 673.1 673.11 673.12 673.13 673.14 |
| Obstetrical blood-clot embolism | 673.2 673.21 673.22 673.23 673.24 |
| Obstetrical pyemic and septic embolism | 673.3 673.33 673.34 |
| Other obstetrical pulmonary embolism | 673.80 673.81 673.83 673.84 |
| Complications of the puerperium | 674.00 674.01 674.02 674.03 674.04 674.1 674.10 674.12 674.20 674.22 674.24 674.30 674.32 674.34 674.40 674.42 674.44 674.8 674.80 674.82 674.84 674.92 |
| Cardiomyopathy-pregnancy | 674.51 674.52 674.54 |
| Complications of the puerperium-Infectious breast | 675.00 675.01 675.02 675.03 675.04 675.10 675.11 675.12 675.13 675.14 675.20 675.21 675.22 675.23 675.24 675.80 675.81 675.83 675.90 |
| Complications of the puerperium-Breast | 675.93 676.00 676.03 676.10 676.11 676.13 676.14 676.20 676.21 676.22 676.23 676.24 676.30 676.31 676.32 676.33 676.34 |
| Complications of the puerperium-Lactation DO | 676.4 676.42 676.5 676.51 676.52 676.6 676.61 676.62 676.63 676.64 676.8 676.80 676.81 676.82 676.83 676.9 676.91 676.93 676.94 |
| Cervical incompetence | 649.70 649.71 649.73 654.50 654.51 654.53 654.54 |
| Rhesus isoimmunization | 656.10 656.11 656.13 |
| Intrauterine death | 656.40 656.41 656.43 |
| Failed induction | 659.00 659.01 659.03 659.10 659.11 659.13 |
| Other obstetrical trauma | 665.00 665.01 665.03 665.10 665.11 665.22 665.3 665.30 665.34 665.40 665.41 665.44 665.50 665.51 665.54 665.6 665.60 665.64 665.7 665.81 665.82 665.83 665.84 665.90 665.91 665.93 |
| Other obstetrical trauma | 665.70 665.72 665.74 |
| Multiple gestation | 651.03 651.13 651.23 651.30 651.31 651.33 651.41 651.43 651.50 651.51 651.53 651.60 651.61 651.63 651.83 651.93 |
| Congenital abnormalities of uterus | 654.00 654.01 654.03 654.04 |
| Complications of birth; puerperium affecting man-Uterus | 654.10 654.11 654.12 654.13 654.14 654.30 654.31 654.33 654.40 654.41 654.42 654.43 654.44 |
| Complications of birth; puerperium affecting man-Cervix | 654.60 654.61 654.62 654.63 |
| Complications of birth; puerperium affecting man-Vagina | 654.70 654.71 654.73 654.74 |
| Complications of birth; puerperium affecting man-Vulva | 654.81 654.82 654.83 654.84 |
| Other and unspecified complications of birth; puerperium affecting man | 654.90 654.91 654.92 654.93 655.00 655.01 655.03 |
| Chromosomal abnormality in fetus | 655.10 655.11 655.13 |
| Hereditary disease in family possibly affecting fetus | 655.20 655.21 655.23 |
| Suspected damage to fetus from viral disease | 655.30 655.31 655.33 |
| Other and unspecified complications of birth; puerperium affecting man | 655.40 655.41 655.43 655.60 655.63 655.70 655.71 655.73 655.80 655.81 655.83 655.90 655.91 655.93 656.00 656.01 656.03 656.20 656.21 656.23 656.50 656.51 656.53 656.60 656.61 656.63 656.70 656.71 656.73 656.80 656.81 656.83 656.90 656.91 656.93 659.20 659.21 659.23 659.30 659.31 659.33 659.41 659.43 659.50 659.51 659.53 659.60 659.61 659.63 659.70 659.71 659.73 659.80 659.81 659.83 659.90 659.91 659.93 667.00 667.02 667.04 667.10 667.12 667.14 668.00 668.01 668.02 668.03 668.04 668.10 668.11 668.13 668.14 668.20 668.21 668.80 668.81 668.82 668.83 668.90 668.91 668.92 668.94 669.00 669.01 669.02 669.03 669.04 669.11 669.12 669.13 669.14 669.20 669.21 669.22 669.23 669.24 669.30 669.32 669.34 669.40 669.41 669.42 669.43 669.44 669.60 669.61 669.70 669.71 669.80 669.81 669.82 669.83 669.84 669.90 669.94 |
| Peripart cardiomy | 674.50 674.53 |
| Late effect of complication of pregnancy, childbirth, and the puerperi | 677. |
| Fetal hematologic-unspec | 678.00 678.01 678.03 |
| Mat comp in utero-p/p | 679.04 |
| Other and unspecified complications of birth; puerperium affecting man | V23. V23.0 V23.1 V23.2 V23.3 V23.5 V23.7 V23.81 V23.82 V23.83 V23.84 V23.86 V23.89 |
| Normal delivery | 650. |
| Multiple gestation | 651.00 651.01 651.10 651.11 651.20 651.21 651.80 651.81 651.9 |
| Outcome of delivery (V codes) | V22.0 V22.1 V22.2 V24.0 V24.1 V24.2 V27.0 V27.1 V27.2 V27.3 V27.4 V27.5 V27.6 V27.7 V27.9 V72.42 |
| Cellulitis and abscess of fingers and toes | 681.0 |
| Cellulitis and abscess of fingers and toes | 681.01 681.02 681.1 681.11 681.9 |
| Other cellulitis and abscess | 682.0 682.1 682.2 682.3 682.4 682.5 682.6 682.7 682.8 682.9 |
| Pyoderma gangrenosum | 686.0 686.01 686.09 |
| Other skin and subcutaneous infections | 021. 021.0 022. 022.0 031.1 032.85 035. 039. 039.0 |
| Carbuncle and furuncle | 680.0 680.1 680.2 680.3 680.4 680.5 680.6 680.7 680.8 680.9 |
| Impetigo | 684. |
| Other skin and subcutaneous infections | 685. 685.0 685.1 686.1 686.8 686.9 |
| Other inflammatory condition of skin-Seborrhea | 690.1 690.11 690.12 690.18 |
| Other erythematosquamous dermatosis | 690.8 |
| Sunburn | 692.76 692.77 |
| Dermatitis herpetiformis | 694.0 694.2 694.3 |
| Subcorneal pustular dermatosis | 694.1 |
| Pemphigus, vulgaris | 694.4 |
| Pemphigoid | 694.5 694.6 694.60 |
| Ocular pemphigus | 694.61 |
| Other specified bullous dermatoses | 694.8 |
| Unspecified bullous dermatoses | 694.9 |
| Toxic erythema/TEN/SJS | 695.0 695.1 695.11 695.13 695.14 695.15 695.19 |
| Erythema nodosum | 695.2 |
| Rosacea | 695.3 |
| Lupus erythematosus | 695.4 |
| Lupus anticoagulant | lupanti.1 |
| Cardiolipin antibody | cadlip.1 |
| ANA antibody | ana.1 |
| Exfol d/t eryth | 695.50 695.51 695.52 695.53 695.54 695.55 695.56 695.57 695.58 695.59 |
| Ritter's disease | 695.81 |
| Other inflammatory condition of skin | 695.89 696.0 696.1 696.2 696.3 696.4 696.5 696.8 697. 697.0 697.1 697.8 698.0 698.1 698.2 698.3 698.4 698.8 698.9 |
| Unspecified erythematous condition | 695.8 695.9 |
| Decubitus ulcer | 707.0 707.01 707.02 707.03 707.04 707.05 707.06 707.07 707.09 707.20 707.21 707.22 707.23 707.24 707.25 |
| Chronic ulcer of leg or foot | 707.10 707.11 707.12 707.13 707.14 707.15 707.19 |
| Other chronic skin ulcer | 707.8 707.9 |
| Other skin disorders | 692.75 700. |
| Other skin disorders | 701.0 701.1 701.2 701.3 701.4 701.5 701.8 701.9 |
| Other skin disorders-Keratosis | 702.0 702.1 702.11 702.19 702.8 |
| Other skin disorders-Nail and hair | 703. 703.0 703.8 704.0 704.00 704.01 704.02 704.09 704.1 704.2 704.3 704.8 704.9 |
| Disorders of sweat glands | 705. 705.8 |
| Hypo/Hyper/Dyshidrosis | 705.0 705.1 705.21 705.22 705.81 |
| Other skin disorders | 705.82 705.83 706. 706.0 706.1 706.2 706.3 706.8 |
| Other skin disorders-Dyschromia | 709.0 709.01 709.09 |
| Other skin disorders | 709.1 709.2 709.3 709.4 709.8 709.9 780.8 782.1 782.2 V13.3 |
| Skin replaced by transplant | V42.3 |
| Infective arthritis and osteomyelitis (except that caused by TB or STD | 003.23 |
| Infective arthritis and osteomyelitis (except that caused by TB or STD | 003.24 026.1 036.82 056.71 711.0 711.00 711.01 711.02 711.03 711.04 711.05 711.06 711.07 711.08 711.09 |
| Reiter's arthropathy | 711.1 711.11 711.12 711.13 711.14 711.15 711.16 711.17 711.18 711.19 |
| Arthropathy in Behcet's syndrome | 711.2 711.21 711.22 711.25 711.26 711.29 |
| Arthropathy associated with other bacterial diseases | 711.3 711.31 711.4 711.41 711.42 711.44 711.45 711.46 711.47 711.48 711.49 |
| Arthropathy associated with other viral diseases | 711.5 711.50 711.51 711.55 711.56 711.57 711.58 711.59 |
| Arthropathy associated with mycoses | 711.6 711.61 711.62 711.63 711.66 711.67 711.69 |
| Arthropathy involving shoulder region associated with helminthiasis | 711.71 711.72 711.76 711.79 711.8 711.81 711.82 711.84 711.85 711.86 711.87 711.88 711.89 |
| Unspecified infective arthritis | 711.9 711.91 711.92 711.93 711.94 711.95 711.96 711.97 711.98 711.99 |
| Osteomyelitis | 730.0 730.01 730.02 730.03 730.04 730.05 730.06 730.07 730.08 730.09 730.1 730.11 730.12 730.13 730.14 730.15 730.16 730.17 730.18 730.19 730.2 730.21 730.22 730.23 730.24 730.25 730.26 730.27 730.28 730.29 |
| Periostitis, without mention of osteomyelitis, involving unspecified s | 730.30 730.31 730.32 730.33 730.34 730.35 730.36 730.37 730.38 730.39 730.7 730.71 730.72 730.74 730.76 730.77 730.78 730.79 730.8 730.81 730.82 730.84 730.85 730.86 730.87 730.88 730.89 730.9 730.91 730.92 730.93 730.94 730.95 730.96 730.97 730.98 730.99 |
| Other specified inflammatory polyarthropathies | 714.8 |
| Felty's syndrome | 714.1 |
| Rheumatoid arthritis and related disease | 714.0 714.2 714.4 714.81 |
| Systemic onset juvenile chronic arthritis | 714.30 714.31 714.32 714.33 |
| Unspecified inflammatory polyarthropathy | 714.9 |
| Ankylosing spondylitis | 720.0 |
| Fibrosis | fibros.1 |
| Osteoarthritis; localized | 715.0 715.00 715.04 715.1 715.11 715.12 715.13 715.14 715.15 715.16 715.17 715.18 715.20 715.21 715.22 715.23 715.24 715.25 715.26 715.27 715.28 715.30 715.31 715.32 715.33 715.34 715.35 715.36 715.37 715.38 715.80 715.89 715.9 715.90 715.91 715.92 715.93 715.94 715.95 715.96 715.97 715.98 V13.4 |
| Arthropathy associated with other endocrine and metabolic disorders | 713.0 |
| Enteropathic arthritis | 713.1 |
| Arthropathy associated with hematological disorders | 713.2 |
| Arthropathy associated with dermatological disorders | 713.3 |
| Arthropathy associated with respiratory disorders | 713.4 |
| Arthropathy associated with neurological disorders | 713.5 |
| Arthropathy associated with hypersensitivity reaction | 713.6 |
| Other non-traumatic joint disorders | 713.7 713.8 |
| Kaschin-Beck disease | 716.0 716.01 716.02 716.03 716.04 716.05 716.06 716.07 716.09 |
| Allergic arthritis | 716.2 716.22 716.23 716.24 716.28 716.29 |
| Climacteric arthritis | 716.3 716.31 716.32 716.33 716.34 716.36 716.39 |
| Transient arthropathy | 716.4 716.41 716.42 716.43 716.44 716.45 716.46 716.47 716.48 716.49 716.5 |
| Polyarthropathy | 716.50 716.51 716.52 716.53 716.54 716.55 716.56 716.57 716.58 716.59 |
| Unspecified monoarthritis | 716.6 716.61 716.62 716.63 716.64 716.65 716.66 716.67 716.68 |
| Other specified arthropathy, site unspecified | 716.80 716.81 716.82 716.83 716.84 716.85 716.86 716.87 716.88 716.89 |
| Arthropathy, unspecified | 716.9 716.91 716.92 716.93 716.94 716.95 716.96 716.97 716.98 716.99 |
| Loose body in joint | 718.1 718.11 718.12 718.13 718.14 718.15 718.17 718.18 718.19 |
| Pathological dislocation | 718.2 718.21 718.22 718.24 718.25 718.26 718.27 718.28 718.29 |
| Ankylosis of joint | 718.5 718.51 718.52 718.53 718.54 718.55 718.56 718.57 718.58 718.59 |
| Other non-traumatic joint disorders | 718.65 718.70 718.71 718.72 718.73 718.74 718.75 718.76 718.77 |
| Other non-traumatic joint disorders | 718.80 718.81 718.82 718.83 718.84 718.85 718.86 718.87 718.88 718.89 718.9 718.91 718.92 718.93 718.94 718.95 718.97 718.98 718.99 |
| Swelling of joint with or without pain | 719.0 |
| Effusion of joint | 719.01 719.02 719.03 719.04 719.05 719.06 719.07 719.08 719.09 |
| Hemarthrosis | 719.1 719.11 719.12 719.13 719.14 719.15 719.16 719.17 719.18 719.19 |
| Villonodular synovitis | 719.2 719.20 719.21 719.22 719.23 719.24 719.25 719.26 719.27 719.28 719.29 |
| Palindromic rheumatism | 719.3 719.31 719.32 719.33 719.34 719.35 719.36 719.37 719.38 719.39 |
| Join pain/stiffness | 719.4 719.41 719.42 719.43 719.44 719.45 719.46 719.47 719.48 719.49 719.50 719.51 719.52 719.53 719.54 719.55 719.56 719.57 719.58 719.59 719.60 719.61 719.62 719.63 719.64 719.65 719.66 719.67 719.68 719.69 |
| Difficulty in walking | 719.7 |
| Joint laxity | 719.80 |
| Other non-traumatic joint disorders-specified | 719.81 719.82 719.83 719.84 719.85 719.86 719.87 719.88 719.89 |
| Other non-traumatic joint disorders-Unspecified | 719.91 719.92 719.93 719.94 719.95 719.96 719.97 719.98 719.99 |
| Other inflammatory spondylopathies | 720.8 |
| Spondylosis and allied disorders | 721.0 721.1 721.2 721.3 721.41 721.42 721.5 721.6 721.7 721.8 721.9 721.90 721.91 |
| Intervertebral disc disorders | 722.0 722.10 722.11 722.2 722.3 722.31 722.32 722.39 722.4 722.51 722.52 722.6 722.70 722.71 722.72 722.73 722.8 722.81 722.82 722.83 722.90 722.91 722.92 722.93 |
| Brachial neuritis or radiculitis NOS | 723.4 |
| Spinal stenosis; lumbar region | 723.0 724.00 724.01 724.02 724.09 |
| Lumbago | 724.2 |
| Sciatica | 724.3 |
| Thoracic or lumbosacral neuritis or radiculitis, unspecified | 724.4 |
| Backache; unspecified | 724.1 724.5 |
| Spinal enthesopathy | 720.1 |
| Sacroiliitis, not elsewhere classified | 720.2 |
| Inflammatory spondylopathies in diseases classified elsewhere | 720.81 |
| Unspecified inflammatory spondylopathy | 720.9 |
| Other back pain and disorders | 723.1 723.2 723.3 723.5 723.6 723.7 723.8 723.9 724. |
| Other back pain and disorders | 724.6 724.7 724.71 724.79 724.8 |
| Osteoporosis | 733. 733.0 733.00 733.01 733.02 733.03 733.09 |
| Pathological fracture | 733.1 733.11 733.12 733.13 733.14 733.15 733.16 733.19 |
| Stress fracture | 733.93 733.94 733.95 733.96 733.97 733.98 V13.52 |
| Acquired foot deformities | 727.1 734. 735.0 735.1 735.2 735.3 735.4 735.5 735.8 735.9 736.7 736.70 736.71 736.72 736.73 736.75 736.76 736.79 |
| Boutonniere/Swan-neck deformity | 736.21 736.22 |
| Contracture of joint | 718.4 718.41 718.42 718.43 718.44 718.45 718.46 718.47 718.48 718.49 |
| Other acquired deformities | 736.00 736.01 736.02 736.03 736.05 736.06 736.07 736.09 736.1 736.2 736.20 736.3 736.31 736.32 736.39 736.41 736.42 736.5 736.6 736.81 736.89 736.9 737.0 737.1 737.10 737.11 737.12 737.19 737.20 737.21 737.22 737.29 737.33 |
| Scoliosis | 737.34 737.39 737.43 |
| Other acquired deformities | 737.41 737.42 737.8 737.9 738.0 738.10 738.11 738.12 738.19 738.2 738.3 738.4 738.5 738.6 738.7 738.8 738.9 |
| Systemic lupus erythematosus encephalitis | 710.0 |
| Systemic sclerosis | 710.1 |
| Sjogren's disease | 710.2 |
| Dermatomyositis | 710.3 |
| Polymyositis | 710.4 |
| Other specified diffuse diseases of connective tissue | 710.8 |
| Unspecified diffuse connective tissue disease | 710.9 |
| Sleep related leg cramps | 327.52 |
| Psoas muscle abscess | 567.31 |
| Eosinophilia myalgia syndrome | 710.5 |
| Polymyalgia rheumatica | 725. |
| Other connective tissue disease | 726.64 726.71 726.72 726.90 |
| Other connective tissue disease | 726.0 726.10 726.11 726.12 726.19 726.2 726.3 726.31 726.32 726.33 726.39 726.4 726.5 726.6 726.61 726.62 726.63 726.65 726.69 726.7 726.73 726.79 726.8 726.91 727. 727.0 727.00 727.01 |
| Giant cell tumor of tendon sheath | 727.02 |
| Other connective tissue disease | 727.03 727.04 727.05 727.06 727.09 727.2 727.3 727.40 727.41 727.42 727.43 727.49 727.5 727.51 727.59 727.6 727.61 |
| Tendon rupture | 727.62 727.63 727.64 727.65 727.66 727.67 727.68 727.69 |
| Hypermobility syndrome | 728.5 |
| Other connective tissue disease | 727.81 727.82 727.83 727.9 728.0 728.10 728.11 728.12 728.13 728.19 728.2 728.3 728.4 728.6 728.7 728.71 728.79 728.81 728.82 728.83 728.84 728.85 728.86 728.87 |
| Rhabdomyolysis | 728.88 |
| Weber-Christian disease | 729.30 |
| Rheumatism, unspecified and fibrositis | 729.0 |
| Other disorder of muscle, ligament, and fascia | 728.89 728.9 |
| Other disorders of soft tissues | 729. |
| Other symptoms involving nervous and musculoskeletal systems | 781.9 |
| Other connective tissue disease | 729.1 729.2 729.3 729.31 729.39 729.4 729.5 729.6 729.71 729.72 729.73 729.79 729.81 729.82 729.90 729.91 729.92 729.99 781.91 781.92 781.99 793.7 V13.5 V43.60 V43.61 V43.62 V43.63 V43.64 V43.65 V43.66 V43.69 V43.7 V45.4 V48.1 V48.2 V48.3 V49.0 V49.1 V49.2 V49.5 V53.7 |
| Other connective tissue disease-amputation | V49.60 V49.61 V49.62 V49.63 V49.64 V49.65 V49.66 V49.67 V49.70 V49.71 V49.72 V49.73 V49.74 V49.75 V49.76 V49.77 |
| Juvenile osteochondrosis | 732.0 732.1 732.3 732.4 732.5 732.6 |
| Hypertrophic pulmonary osteoarthropathy | 731.2 |
| Osteochondritis dissecans | 732.7 |
| Unspecified osteochondropathy | 732.9 |
| Other bone disease and musculoskeletal deformities | 729.8 731.0 731.1 731.3 731.8 732.2 732.8 733.2 733.22 733.29 733.3 |
| Avascular necrosis/osteonecrosis | 733.4 733.41 733.42 733.43 733.44 733.45 733.49 ostnec.1 |
| Osteitis condensans | 733.5 |
| Tietze's disease | 733.6 |
| Algoneurodystrophy | 733.7 |
| Osteopenia | 733.90 |
| Arrest of bone development or growth | 733.91 |
| Chondromalacia | 733.92 |
| Relapsing polychondritis | 733.99 |
| Bone replaced by transplant | V42.4 |
| Infantile idiopathic scoliosis | 737.31 737.32 |
| Other bone disease and musculoskeletal deformities | 733.81 733.82 737.30 739.0 739.1 739.2 739.3 739.4 739.5 739.6 739.7 739.8 739.9 |
| Disfigurements | V48.6 V48.7 V49.4 |
| Cardiac and circulatory congenital anomalies | 746.8 |
| Other specified congenital anomalies of circulatory system | 747.8 |
| Transposition of great vessels | 745.10 745.12 745.19 |
| Pulmonary artery stenosis | 747.31 |
| Pulmonary AV malformatn | 747.32 |
| Cerebral arteriovenous malformation | 747.81 |
| Taussig-Bing syndrome or defect | 745.11 |
| Common ventricle | 745.3 |
| Congenital insufficiency of aortic valve | 746.4 |
| Hypoplastic left heart syndrome | 746.7 |
| Congenital anomaly of gastrointestinal vessel | 747.61 |
| Persistent fetal circulation | 747.83 |
| Common truncus | 745.0 |
| Cor biloculare | 745.7 |
| Other bulbus cordis anomalies and anomalies of cardiac septal closure | 745.8 |
| Unspecified defect of septal closure | 745.9 |
| Congenital tricuspid disease | 746.1 746.2 |
| Congenital mitral disease | 746.5 746.6 |
| Tetralogy of Fallot | 745.2 |
| Subaortic stenosis, congenital | 746.81 |
| Cor triatriatum | 746.82 |
| Infundibular pulmonic stenosis, congenital | 746.83 |
| Uhl's disease | 746.84 |
| Coronary artery anomaly, congenital | 746.85 |
| Congenital heart block | 746.86 |
| Malposition of heart and cardiac apex | 746.87 |
| Pericardial anomaly | 746.89 |
| Unspecified congenital anomaly of heart | 746.9 |
| Other congenital anomalies of aorta | 747.11 747.2 747.20 747.21 747.22 747.29 |
| Other circulatory congenital anomalies-great veins | 747.4 747.49 |
| Anomalous pulmonary venous connection | 747.41 747.42 |
| Other circulatory congenital anomalies-artery/venous | 747.5 747.6 747.60 |
| Congenital anomaly of Renal vessel | 747.62 |
| Congenital anomalies of peripheral vascular system | 747.63 747.64 747.69 747.82 |
| Unspecified congenital anomaly of circulatory system | 747.9 |
| Ventricular septal defect | 745.4 |
| Patent foramen ovale | 745.5 |
| Endocardial cushion defects | 745.6 745.61 745.69 |
| Pulmonary valve atresia and stenosis | 746.0 746.01 746.02 746.09 |
| Congenital stenosis of aortic valve | 746.3 |
| Patent ductus arteriosus | 747.0 |
| Coarctation of aorta | 747.10 |
| Tracheoesophageal fistula, esophageal atresia and stenosis, congenital | 750.3 |
| Congenital hypertrophy of pylorus | 750.5 |
| Imperforate anus | 751.2 |
| Hirschsprung's disease and other congenital functional disorders of co | 751.3 |
| Meckel's diverticulum | 751.0 |
| Atresia of ileum | 751.1 |
| Congenital malrotation of intestine | 751.4 |
| Congenital anomalies of tongue | 750.0 750.1 750.10 750.11 750.12 750.13 750.15 750.16 |
| Congenital anomalies of salivary gland | 750.21 750.22 750.23 |
| Other specified congenital anomalies of mouth | 750.25 750.26 |
| Other specified congenital anomalies of pharynx | 750.27 750.29 |
| Other specified congenital anomalies of esophagus | 750.4 |
| Congenital hiatus hernia | 750.6 |
| Other upper gastrointestinal congenital anomalies | 750.7 750.8 750.9 |
| Other congenital anomalies of intestine | 751.5 |
| Other lower gastrointestinal congenital anomalies | 751.60 751.61 751.62 751.69 |
| Polycystic disease of pancreas | 751.7 |
| Other specified congenital anomalies of digestive system | 751.8 |
| Unspecified congenital anomaly of digestive system | 751.9 |
| Undescended testis | 752.51 |
| Retractile testis | 752.52 |
| Hypospadias and epispadias | 752.61 752.62 V13.61 |
| Obstructive genitourinary defect | 753.2 753.21 753.22 753.23 753.29 |
| Polycystic kidney, congenital | 753.12 753.13 |
| Polycystic kidney, autosomal recessive | 753.14 |
| Other cystic kidney disease | 753.1 753.11 |
| Renal dysplasia | 753.15 |
| Other cystic kidney disease--Medullary | 753.16 753.17 |
| Other specified cystic kidney disease | 753.19 |
| Embryonic cyst of fallopian tubes and broad ligaments | 752.11 |
| Other congenital anomalies of genitalia-female | 752. 752.0 752.1 752.19 752.2 752.34 752.36 752.4 752.41 752.42 752.49 752.63 |
| Other congenital anomalies of genitalia-Male | 752.64 752.65 752.69 752.7 752.8 752.81 752.89 |
| Unilateral agenesis of kidney | 753.0 |
| Other specified congenital anomalies of kidney | 753.3 |
| Other congenital anomalies of urinary system | 753. 753.4 753.5 753.6 753.7 753.8 753.9 |
| Other specified congenital anomalies of spinal cord | 742.5 |
| Spina bifida | 741. 741.0 741.01 741.02 741.03 741.9 741.90 741.91 741.92 741.93 742.3 |
| Other nervous system congenital anomalies | 740.0 740.1 740.2 742.0 742.1 742.2 742.4 742.51 742.53 742.59 742.8 742.9 |
| Other congenital anomalies | 744.8 754.8 756.8 757.3 |
| Cleft palate without cleft lip | 749. 749.0 749.01 749.02 749.03 749.04 |
| Congenital hip dislocation | 754.3 754.30 754.31 754.32 754.33 754.35 |
| Polydactyly (extra digits) and syndactyly (webbing between digits) | 755.0 755.01 755.02 755.10 755.11 755.12 755.13 755.14 |
| Congenital absence of lower limb | 755.3 755.31 755.32 755.33 755.34 755.35 755.36 755.37 755.38 755.39 |
| Congenital hip deformity | 755.61 755.62 755.63 |
| Other congenital anomalies of limbs | 754.42 755.2 755.21 755.22 755.23 755.24 755.25 755.26 755.27 755.28 755.29 755.4 755.5 755.50 755.51 755.52 755.53 755.54 755.55 755.56 755.57 755.58 755.6 755.60 755.64 755.65 755.66 755.67 755.8 755.9 |
| Masklike facies | 756.0 |
| Spondylolysis, congenital, lumbosacral region | 756.11 |
| Spondylolisthesis | 756.12 |
| Other spinal congenital anomalies | 756.1 756.13 756.14 756.15 756.16 756.17 756.19 |
| Osteogenesis imperfecta | 756.51 |
| Cleft lip with or without cleft palate | 749.1 749.11 749.12 749.13 749.14 749.21 749.22 749.23 749.24 749.25 |
| Eventration of diaphragm | 756.6 |
| Anomalies of abdominal wall, congenital | 756.7 |
| Prune belly syndrome | 756.71 |
| Omphalocele | 756.72 |
| Gastroschisis | 756.73 |
| Other congenital anomalies of abdominal wall | 756.79 |
| Xeroderma pigmentosum | 757.33 |
| Hereditary lymphedema | 757.0 |
| Vascular hamartomas | 757.32 |
| Other congenital anomalies of integument | 757.1 757.2 757.31 757.39 757.4 757.5 757.8 757.9 |
| Specified congenital anomalies of breast | 757.6 |
| Pectus excavatum | 754.81 |
| Trisomy 22 | 758.0 |
| Ehlers-Danlos syndrome | 756.83 |
| All other congenital anomalies-Musculoskeletal | 754.0 754.1 754.2 754.40 754.41 754.43 754.44 754.82 756.2 756.3 756.4 756.5 756.53 756.54 756.55 756.56 756.82 |
| All other congenital anomalies | 756.89 758.1 758.2 758.31 758.32 758.33 758.39 758.4 758.5 758.6 758.7 758.8 758.81 758.9 759.1 759.2 759.4 759.5 759.6 759.7 759.8 759.81 759.83 759.89 759.9 795.2 V13.69 |
| Other congenital osteodystrophies | 756.59 |
| Absence of muscle and tendon, congenital | 756.81 |
| Osteopetrosis | 756.52 |
| Other and unspecified congenital anomalies of musculoskeletal system | 756.9 |
| Situs inversus | 759.3 |
| Marfanoid physique | 759.82 |
| Congenital absent spleen | 759.0 |
| Congenital eye disorders | 743.0 743.03 743.1 743.12 743.2 743.21 743.22 743.30 743.31 743.32 743.33 743.34 743.35 743.36 743.37 743.39 743.41 743.42 743.43 743.44 743.45 743.46 743.47 743.48 743.49 743.51 743.52 743.53 743.54 743.55 743.56 743.57 743.58 743.59 743.61 743.62 743.63 743.64 743.65 743.66 743.69 743.8 743.9 |
| Microtia | 744.23 |
| Branchial cleft cyst or fistula | 744.41 744.42 |
| Other face and neck congenital anomalies | 744.0 744.01 744.02 744.03 744.04 744.05 744.09 744.1 744.21 744.22 744.24 744.29 744.3 744.43 744.46 744.47 |
| Other face and neck congenital anomalies | 744.49 744.5 744.81 744.82 744.83 744.84 744.89 744.9 |
| Congenital anomalies of larynx trachea and bronchus | 748.2 748.3 |
| Other respiratory congenital anomalies | 748. 748.0 748.1 748.4 748.5 748.6 748.60 748.61 748.8 |
| Foot deformities | 754.5 754.51 754.52 754.53 754.59 754.60 754.61 754.62 754.69 754.7 754.70 754.71 754.79 |
| Perinatal disorders of digestive system | 777. |
| Liveborn | 765.20 765.29 V30.00 V30.01 V30.1 V30.2 V31.00 V31.01 V31.1 V31.2 V32.00 V32.01 V32.1 V32.2 V33.00 V33.01 V33.1 V33.2 V34.00 V34.01 V34.1 V34.2 V35.00 V35.01 V35.2 V36.00 V36.01 V37.00 V37.01 V37.2 V39.00 V39.1 |
| Low birth weight infant | 764.00 764.01 764.02 764.03 764.04 764.05 764.06 764.07 764.08 764.09 764.1 764.11 764.14 764.15 764.16 764.17 764.18 764.19 |
| Fetal malnutrition | 764.2 764.21 764.23 764.24 764.25 764.27 764.28 764.29 764.90 |
| Fetal growth retardation | 764.9 764.91 764.92 764.93 764.94 764.95 764.96 764.97 764.98 764.99 |
| Short gestation; low birth weight; and fetal growth retardation | 765.00 765.01 765.02 765.03 765.04 765.05 765.06 765.07 765.08 765.09 765.1 765.10 765.11 765.12 765.13 765.14 765.15 765.16 765.17 765.18 765.19 765.21 765.22 765.23 765.24 765.25 765.26 765.27 765.28 |
| Low birth wt | V21.30 V21.31 V21.32 V21.33 V21.34 V21.35 |
| Intrauterine hypoxia and birth asphyxia | 768.0 768.1 768.2 768.3 768.4 768.5 768.6 768.70 768.71 768.72 768.73 768.9 770.88 |
| Respiratory distress syndrome in newborn | 769. |
| Hemolytic jaundice and perinatal jaundice | 773. 773.0 773.1 773.3 773.4 773.5 |
| Perinatal jaundice from hereditary hemolytic anemias | 774.0 |
| Neonatal jaundice due to delayed conjugation in diseases classified el | 774.31 |
| Hemolytic jaundice and perinatal jaundice | 774.1 774.2 774.30 774.39 774.4 774.5 774.6 774.7 |
| Birth trauma | 767. 767.0 767.11 767.19 767.2 767.3 767.4 767.5 767.6 767.7 767.8 |
| Other perinatal conditions | 780.91 780.92 789.7 |
| Neonatal bradycardia | 779.81 |
| Neonatal tachycardia | 779.82 |
| Thrombophlebitis of the newborn | 779.89 |
| Other perinatal conditions | 770.0 770.2 770.3 770.4 770.5 770.6 770.7 770.81 770.82 770.83 770.84 770.87 770.89 770.9 |
| Infections specific to the perinatal period | 771.0 771.1 771.2 |
| Other perinatal conditions | 040.41 771.3 771.4 771.5 771.6 771.7 771.8 771.82 771.83 |
| Endocrine and metabolic disturbances of fetus and newborn | 775.4 775.5 |
| Neonatal hypoglycemia | 775.6 |
| Endocrine and metabolic disturbances of fetus and newborn | 775.0 775.1 775.2 775.3 |
| Endocrine and metabolic disturbances of fetus and newborn | 775.7 775.81 775.89 |
| Unspecified endocrine and metabolic disturbances specific to the fetus | 775.9 |
| Other and unspecified perinatal conditions | 760.0 760.1 760.2 760.3 760.4 760.5 760.61 760.63 760.70 760.74 760.76 760.77 760.78 760.79 760.8 760.9 761. 761.8 |
| Incompetent cervix affecting fetus or newborn | 761.0 |
| Premature rupture of membranes affecting fetus or newborn | 761.1 |
| Oligohydramnios affecting fetus or newborn | 761.2 |
| Polyhydramnios affecting fetus or newborn | 761.3 |
| Ectopic pregnancy affecting fetus or newborn | 761.4 |
| Multiple pregnancy affecting fetus or newborn | 761.5 |
| Maternal death affecting fetus or newborn | 761.6 |
| Other and unspecified perinatal conditions | 761.7 763.0 763.1 |
| Other and unspecified perinatal conditions-Placental | 762.0 762.1 762.2 |
| Other and unspecified perinatal conditions-Umbilical cord | 762.4 762.5 762.6 |
| Placental transfusion syndromes affecting fetus or newborn | 762.3 |
| Forceps/vaccum delivery | 763.2 763.3 |
| Chorioamnionitis affecting fetus or newborn | 762.7 |
| Other and unspecified perinatal conditions-Chorion | 762.8 762.9 |
| Cesarean delivery affecting fetus or newborn | 763.4 |
| Other and unspecified perinatal conditions | 763.5 763.6 763.7 763.8 763.81 763.82 763.83 |
| Meconium dur del aff NB | 763.84 |
| Other and unspecified perinatal conditions-Post-term | 766.0 766.21 766.22 |
| Unspecified complication of labor and delivery affecting fetus or newb | 763.9 |
| Other heavy-for-dates infants | 766.1 |
| Meconium asp | 770.11 770.12 |
| Other and unspecified perinatal conditions | 770.10 770.13 770.14 770.15 770.16 770.17 770.18 770.85 770.86 |
| Fetal blood loss affecting newborn | 772.0 |
| Intraventricular hemorrhage of fetus or newborn | 772.1 772.10 772.11 772.12 772.13 772.14 779.7 |
| Subarachnoid hemorrhage of newborn | 772.2 |
| Other and unspecified perinatal conditions-Hemorrhage | 772.3 772.4 772.5 772.6 772.8 772.9 |
| Hemorrhagic disease of newborn | 776.0 |
| Other and unspecified perinatal conditions-HEME/DIC/Coagulation | 776.1 776.2 776.3 |
| Polycythemia neonatorum | 776.4 |
| Congenital anemia | 776.5 |
| Anemia of prematurity | 776.6 |
| Transient neonatal neutropenia | 776.7 |
| Other specified transient hematological disorders of fetus or newborn | 776.8 |
| Neonatal coagulation disorder | 776.9 |
| Meconium obstruction in fetus or newborn | 777.1 |
| Intestinal obstruction due to inspissated milk in newborn | 777.2 |
| Hematemesis and melena due to swallowed maternal blood of newborn | 777.3 |
| Transitory ileus of newborn | 777.4 |
| Other and unspecified perinatal conditions-Enterocolitis | 777.50 777.51 777.52 777.53 |
| Perinatal intestinal perforation | 777.6 |
| Paralytic ileus of the newborn | 777.8 |
| Hydrops fetalis not due to isoimmunization | 778.0 |
| Sclerema neonatorum | 778.1 |
| Other and unspecified perinatal conditions-Temperature | 778.2 778.3 778.4 778.9 |
| Other and unspecified edema of newborn | 778.5 |
| Congenital hydrocele | 778.6 |
| Other and unspecified perinatal conditions | 778.7 778.8 |
| Other and unspecified perinatal conditions-Convulsions/encephalopathy | 779.0 779.2 |
| Bilious vomiting in newborn | 779.32 |
| Other and unspecified cerebral irritability in newborn | 779.1 |
| Feeding problems in newborn | 779.31 |
| Vomiting in newborn | 779.33 |
| Failure to thrive in newborn | 779.34 |
| Gray syndrome from chloramphenicol administration in newborn | 779.4 |
| Termination of pregnancy (fetus) | 779.6 |
| Other specified conditions originating in the perinatal period | 779.8 |
| Delay separate umbl cord | 779.83 |
| NB cardiac arrest | 779.85 |
| Unspecified condition originating in the perinatal period | 779.9 |
| Personal history of perinatal problems | V13.7 |
| Routine or ritual circumcision | V50.2 |
| Injury and poisoning | 717.8 |
| Injury and poisoning | 839.6 839.7 862.2 862.3 900.8 901.8 902.8 926.1 |
| Traumatic arthropathy | 716.1 716.11 716.12 716.13 716.14 716.15 716.16 716.17 716.18 716.19 |
| Joint disorders and dislocations; trauma-related | 717. 717.0 717.1 717.2 717.3 717.4 717.41 717.42 717.43 717.49 717.5 717.6 717.7 717.81 717.82 717.83 717.84 717.85 |
| Articular cartilage disorder | 718.0 718.01 718.02 718.03 718.04 718.05 718.07 718.08 718.09 |
| Recurrent dislocation of joint | 718.3 718.31 718.32 718.33 718.34 718.35 718.36 718.37 718.38 718.39 |
| Dislocation of jaw | 830. 830.0 830.1 |
| Joint disorders and dislocations; trauma-related | 831.00 831.01 831.02 831.03 831.04 831.09 831.10 831.11 831.12 831.14 831.19 832.00 832.01 832.02 832.03 832.04 832.09 832.1 832.11 832.12 832.13 832.14 832.19 832.2 833.0 833.01 833.02 833.03 833.04 833.05 833.09 833.10 833.11 833.12 833.13 833.14 833.15 833.19 834.00 834.01 834.02 834.10 834.11 834.12 835. 835.00 835.01 835.02 835.03 835.10 835.11 835.13 836.0 836.1 836.2 836.3 836.4 836.5 836.50 836.51 836.52 836.53 836.54 836.6 836.60 836.61 836.62 836.63 836.64 837.0 837.1 838.00 838.01 838.02 838.03 838.04 838.05 838.06 838.09 838.10 838.11 838.12 838.13 838.14 838.15 838.16 838.19 839.0 839.01 839.02 839.03 839.04 839.05 839.06 839.07 839.08 839.1 839.11 839.12 839.13 839.14 839.18 839.20 839.21 839.30 839.31 839.4 839.40 839.41 839.42 839.52 839.61 839.69 839.71 839.8 839.9 905.6 |
| Coronary atherosclerosis of artery bypass graft | 414.02 414.03 414.04 414.05 414.07 |
| Atherosclerosis of bypass graft | 440.30 440.31 440.32 |
| Malfunction of device; implant; and graft | 569.61 569.69 596.82 996.0 996.01 996.02 996.03 996.04 996.09 996.1 996.2 996.3 996.31 996.32 996.39 996.40 996.41 996.42 996.43 996.44 996.45 996.46 996.47 996.49 996.51 996.52 996.53 996.54 996.55 996.56 996.57 996.59 |
| Infection and inflammation--internal prosthetic device; implant; and g | 996.60 996.61 996.62 996.63 996.64 996.65 996.66 996.67 996.68 996.69 999.31 |
| Other complications of internal prosthetic device; implant; and graft | 996.70 996.71 996.72 996.73 996.74 996.75 996.76 996.77 996.78 996.79 |
| Graft-versus-host disease | 279.5 279.51 279.52 279.53 |
| Complications of other transplanted organ | 996.80 996.89 |
| Renal transplant rejection | 996.81 |
| Complications of transplanted liver | 996.82 |
| Complications of transplanted heart | 996.83 |
| Complications of transplanted lung | 996.84 |
| Complications of bone marrow transplant | 996.85 |
| Complications of transplanted pancreas | 996.86 |
| Comp intestine transplnt | 996.87 |
| Complications of transplants and reattached limbs | 996.90 996.91 996.92 996.93 996.94 996.95 996.96 996.99 |
| Complications of surgical procedures or medical care | 998.31 998.32 V15.8 V15.80 V15.83 |
| Cardiac complications | 429.4 458.21 458.29 997.1 |
| Respiratory complications | 518.7 997.31 997.39 |
| Gastrointestinal complications | 564.2 564.3 564.4 569.6 569.71 569.79 579.3 997.41 997.49 |
| Urinary complications | 596.81 997.5 |
| Hemorrhage or hematoma complicating a procedure | 998.11 998.12 998.13 |
| Postoperative infection | 519.01 530.86 536.41 997.62 998.51 998.59 |
| Tumor lysis syndrome | 277.88 |
| Anemia d/t antineo chemo | 285.3 |
| Malignant hyperthermia | 995.86 |
| Hypertension | 997.91 |
| Other complications of surgical and medical procedures | 277.83 349.0 349.1 349.31 415.11 512.1 519.00 519.02 519.09 530.87 536.40 536.42 536.49 569.62 780.62 780.63 909.3 995.4 997.00 997.01 997.02 997.09 997.2 997.60 997.61 997.69 997.71 997.72 997.79 997.99 998.2 998.30 998.33 998.4 998.6 998.7 998.81 998.82 998.83 998.89 998.9 999.0 999.1 999.2 999.39 999.5 999.81 999.82 999.88 999.9 |
| Other transfusion reaction | 999.89 |
| Hemolytic transfusion reaction | 999.83 |
| Anaphylaxis | 999.4 |
| Poisoning by psychotropic agents | 969. |
| Poison-antidepresnt NOS | 969.00 |
| Pois monoamine oxidase | 969.01 |
| Pois serotinin reuptake | 969.03 |
| Pois tetracyclc andepres | 969.04 |
| Pois tricyclc antidepres | 969.05 |
| Pois antidepressants NEC | 969.09 |
| Poisoning by psychotropic agents | 969.1 969.2 969.3 969.4 969.5 969.6 969.71 969.72 969.73 969.79 969.8 |
| Mucositis d/t drugs NEC | 528.01 528.02 |
| Poisoning by penicillins | 960.0 |
| Poisoning by antifungal antibiotics | 960.1 |
| Poisoning by chloramphenicol group | 960.2 |
| Poisoning by erythromycin and other macrolides | 960.3 |
| Poisoning by tetracycline group | 960.4 |
| Poisoning of cephalosporin group | 960.5 |
| Poisoning by anticoagulants | 964.2 964.5 |
| Poisoning-vitamin k | 964.3 |
| Poisoning by natural blood and blood products | 964.7 |
| Poisoning by cardiac rhythm regulators | 972.0 |
| Poisoning by other antihypertensive agents | 972.6 |
| Poisoning by other medications and drugs | 909.0 909.5 960. 960.6 960.7 960.8 961.0 961.1 961.2 961.4 961.5 961.6 961.7 961.8 961.9 962.0 962.1 962.2 962.3 962.4 962.5 962.6 962.7 962.8 962.9 963. 963.0 963.1 963.2 963.3 963.4 963.5 963.8 964.0 964.1 964.4 964.6 964.8 964.9 965.1 965.4 965.61 965.69 965.7 965.8 965.9 966.0 966.1 966.2 966.3 966.4 967. 967.0 967.1 967.2 967.3 967.5 967.6 967.8 968.0 968.1 968.2 968.3 968.4 968.5 968.6 968.7 968.9 970. 970.0 970.1 971.0 971.1 971.2 971.3 971.9 972.2 972.3 972.4 972.5 972.7 972.9 973.0 973.1 973.2 973.3 973.4 973.5 973.6 973.8 973.9 974.0 974.1 974.2 974.6 974.7 975.0 975.1 975.2 975.3 975.4 975.5 975.6 975.7 975.8 976.0 976.1 976.2 976.3 976.4 976.5 976.6 976.7 976.8 976.9 977.0 977.1 977.2 977.3 977.4 977.8 977.9 978.0 978.1 978.2 978.4 978.6 978.8 978.9 979. 979.0 979.1 979.3 979.4 979.6 979.7 995.20 995.21 995.22 995.23 995.27 995.29 |
| Poisoning by other diuretics | 974.3 974.4 |
| Poisoning by electrolytic, caloric, and water-balance agents | 974.5 |
| Poisoning by cardiotonic glycosides and drugs of similar action | 972.1 |
| Poisoning by nonmedicinal substances | 909.1 980.1 980.2 980.3 980.8 981. 982.0 982.1 982.2 982.3 982.4 982.8 983.0 983.1 983.2 983.9 984.0 984.1 984.8 984.9 985.0 985.1 985.2 985.3 985.4 985.5 985.6 985.8 985.9 986. 987.0 987.1 987.2 987.3 987.4 987.5 987.6 987.7 987.8 987.9 988. 988.1 988.2 988.8 989. 989.0 989.1 989.2 989.3 989.4 989.5 989.6 989.7 989.81 989.82 989.83 989.84 989.89 989.9 |
| Toxic effect of fish and shellfish eaten as food | 988.0 |
| Other injuries and conditions due to external causes | 796. 796.0 799.01 799.02 907.1 907.3 907.4 907.5 907.9 908.5 908.6 908.9 909.2 909.4 909.9 930. 930.0 930.1 930.2 930.8 931. 932. 933.0 933.1 934.0 934.1 934.8 934.9 935.0 935.1 935.2 936. 937. 938. 939. 939.0 939.1 939.2 939.3 950. 950.0 950.1 950.2 950.3 951.0 951.1 951.2 951.3 951.4 951.5 951.6 951.7 951.8 951.9 953. 953.0 953.1 953.2 953.3 953.4 953.5 953.8 954.0 954.1 954.8 954.9 955. 955.0 955.1 955.2 955.3 955.4 955.5 955.6 955.7 955.8 956.0 956.1 956.2 956.3 956.4 956.5 956.8 956.9 957.0 957.1 957.8 957.9 958.0 958.1 958.2 958.3 958.4 958.5 958.6 958.7 958.8 958.90 958.91 958.92 958.93 958.99 959.01 959.09 959.11 959.12 959.13 959.14 959.19 959.2 959.3 959.4 959.5 959.6 959.7 959.8 959.9 990. 991.0 991.1 991.2 991.3 991.4 991.5 991.6 991.8 991.9 992.0 992.1 992.2 992.3 992.4 992.5 992.6 992.7 992.8 992.9 993.0 993.1 993.2 993.3 993.4 993.8 993.9 994.0 994.1 994.2 994.3 994.4 994.5 994.6 994.7 994.8 994.9 995.1 995.5 995.51 995.52 995.53 995.54 995.55 995.59 995.8 995.80 995.81 995.82 995.83 995.84 995.85 V15.51 V15.59 V15.6 V15.88 V71.3 V71.4 V71.5 V71.6 |
| Sepsis/SIRS | 995.9 995.91 995.92 |
| SIRS-noninf | 995.93 995.94 |
| Fractures | 808.4 808.5 |
| Fracture of neck of femur (hip) | 820. 820.00 820.01 820.02 820.03 820.09 820.10 820.11 820.12 820.13 820.19 820.20 820.21 820.22 820.30 820.31 820.32 820.8 820.9 905.3 hipfx.1 V54.13 V54.23 |
| Skull and face fractures | 800.00 800.01 800.02 800.03 800.05 800.06 800.09 800.50 800.51 800.52 800.53 800.55 800.56 800.59 801.00 801.01 801.02 801.03 801.04 801.05 801.06 801.09 801.50 801.51 801.52 801.53 801.55 801.56 801.59 802.0 802.1 802.20 802.21 802.22 802.23 802.24 802.25 802.26 802.27 802.28 802.29 802.3 802.31 802.32 802.33 802.34 802.35 802.36 802.37 802.38 802.39 802.4 802.5 802.6 802.7 802.8 802.9 803.00 803.01 803.02 803.03 803.04 803.05 803.06 803.09 803.50 803.51 803.52 803.56 803.59 804.00 804.01 804.02 804.03 804.04 804.05 804.06 804.09 804.50 804.51 804.52 804.53 804.59 905.0 |
| Fracture of upper limb | 810.0 810.01 810.02 810.03 810.1 810.11 810.12 810.13 811.0 811.01 811.02 811.03 811.09 811.1 811.11 811.12 811.13 811.19 812.00 812.01 812.02 812.03 812.09 812.10 812.11 812.12 812.13 812.19 812.20 812.21 812.30 812.31 812.4 812.41 812.42 812.43 812.44 812.49 812.5 812.51 812.52 812.53 812.54 812.59 813.0 813.00 813.01 813.02 813.03 813.04 813.05 813.06 813.07 813.1 813.10 813.11 813.12 813.13 813.14 813.15 813.16 813.17 813.2 813.20 813.21 813.22 813.3 813.30 813.31 813.32 813.4 813.40 813.41 813.42 813.43 813.45 813.46 813.47 813.5 813.50 813.51 813.52 813.53 813.8 813.80 813.81 813.82 813.9 813.90 813.91 813.92 814. 814.0 814.01 814.02 814.03 814.04 814.05 814.06 814.07 814.08 814.09 814.1 814.11 814.12 814.13 814.14 814.15 814.16 814.17 814.18 814.19 815.0 815.01 815.02 815.03 815.04 815.09 815.1 815.11 815.12 815.13 815.14 815.19 816.0 816.01 816.02 816.03 816.1 816.11 816.12 816.13 817.0 817.1 818.0 818.1 819.0 819.1 905.2 V54.10 V54.11 V54.12 V54.20 V54.21 V54.22 |
| Fracture of lower limb | 823.0 823.00 823.01 823.1 823.10 823.11 823.2 823.20 823.21 823.3 823.30 823.31 823.40 823.41 823.42 823.8 823.80 823.81 823.9 823.90 823.91 V54.14 V54.15 V54.16 V54.24 V54.25 V54.26 |
| Fracture of ankle | 824. 824.0 824.1 824.2 824.3 824.4 824.5 824.6 824.7 824.8 824.9 |
| Other fracture of lower limb | 821.00 821.01 821.10 821.11 821.20 821.21 821.22 821.23 821.29 821.30 821.31 821.32 821.33 821.39 822.0 822.1 825.0 825.1 825.2 825.20 825.21 825.22 825.23 825.24 825.25 825.3 825.30 825.31 825.32 825.33 825.34 825.35 826.0 826.1 827.0 827.1 905.4 V54.19 V54.29 |
| Fracture of vertebral column without mention of spinal cord injury | 805.00 805.01 805.02 805.03 805.04 805.05 805.06 805.07 805.08 805.10 805.11 805.12 805.13 805.14 805.15 805.16 805.17 805.18 805.2 805.3 805.4 805.5 805.6 805.7 805.8 805.9 V54.17 V54.27 |
| Fracture of ribs; closed | 807.0 807.00 807.01 807.02 807.03 807.04 807.05 807.06 807.07 807.08 807.09 |
| Fracture of pelvis | 808. 808.0 808.1 808.2 808.3 808.41 808.42 808.43 808.51 808.52 808.53 808.8 808.9 |
| Other and unspecified fracture | 807.1 807.11 807.12 807.13 807.14 807.16 807.17 807.18 807.19 807.2 807.3 807.4 807.5 807.6 809.0 809.1 828.0 828.1 829. 829.0 829.1 905.1 905.5 V54. V54.01 V54.02 V54.09 V66.4 V67.4 |
| Spinal cord injury | 349.39 806.00 806.01 806.02 806.03 806.04 806.05 806.06 806.07 806.08 806.09 806.10 806.11 806.12 806.13 806.14 806.15 806.16 806.17 806.18 806.19 806.20 806.21 806.22 806.23 806.24 806.25 806.26 806.27 806.28 806.29 806.30 806.31 806.32 806.33 806.34 806.35 806.36 806.37 806.38 806.39 806.4 806.5 806.6 806.61 806.62 806.69 806.7 806.71 806.72 806.79 806.8 806.9 907.2 952.00 952.01 952.02 952.03 952.04 952.05 952.06 952.07 952.08 952.09 952.10 952.11 952.12 952.13 952.14 952.15 952.16 952.17 952.18 952.19 952.2 952.3 952.4 952.8 952.9 |
| TBI/Concussion | 850. 850.0 850.11 850.12 850.2 850.3 850.4 850.5 V15.52 |
| Other intracranial injury | 800.10 800.11 800.12 800.13 800.14 800.15 800.16 800.19 800.20 800.21 800.22 800.23 800.24 800.25 800.26 800.29 800.30 800.31 800.32 800.33 800.34 800.35 800.36 800.39 800.40 800.41 800.42 800.43 800.45 800.46 800.49 800.60 800.61 800.62 800.63 800.64 800.65 800.66 800.69 800.70 800.71 800.72 800.73 800.74 800.75 800.76 800.79 800.80 800.81 800.82 800.83 800.84 800.85 800.86 800.90 800.91 800.92 800.95 800.96 800.99 801.10 801.11 801.12 801.13 801.14 801.15 801.16 801.19 801.20 801.21 801.22 801.23 801.24 801.25 801.26 801.29 801.30 801.31 801.32 801.33 801.34 801.35 801.36 801.39 801.40 801.41 801.42 801.43 801.44 801.45 801.46 801.49 801.60 801.61 801.62 801.63 801.64 801.65 801.66 801.69 801.70 801.71 801.72 801.73 801.74 801.75 801.76 801.79 801.80 801.81 801.85 801.86 801.89 801.90 801.91 801.92 801.94 801.95 801.96 801.99 803.10 803.11 803.12 803.14 803.15 803.16 803.19 803.20 803.21 803.22 803.23 803.24 803.25 803.26 803.29 803.30 803.31 803.32 803.33 803.35 803.36 803.39 803.40 803.41 803.42 803.43 803.45 803.46 803.49 803.60 803.61 803.62 803.65 803.66 803.70 803.71 803.72 803.73 803.74 803.75 803.76 803.79 803.80 803.81 803.82 803.83 803.85 803.86 803.89 803.90 803.91 803.92 803.93 803.95 803.96 804.10 804.11 804.13 804.14 804.15 804.16 804.19 804.20 804.21 804.22 804.23 804.25 804.26 804.29 804.30 804.31 804.32 804.33 804.35 804.36 804.39 804.40 804.41 804.43 804.46 804.49 804.60 804.61 804.66 804.70 804.71 804.72 804.73 804.75 804.76 804.80 804.81 804.85 804.90 851.00 851.01 851.02 851.03 851.04 851.05 851.06 851.09 851.10 851.11 851.12 851.13 851.14 851.15 851.16 851.20 851.21 851.22 851.23 851.24 851.25 851.26 851.30 851.31 851.32 851.33 851.34 851.35 851.36 851.39 851.40 851.41 851.42 851.43 851.44 851.45 851.46 851.49 851.50 851.51 851.52 851.53 851.55 851.56 851.59 851.60 851.61 851.65 851.66 851.70 851.71 851.75 851.76 851.80 851.81 851.82 851.83 851.84 851.85 851.86 851.89 851.90 851.91 851.92 851.93 851.94 851.95 851.96 851.99 852.00 852.01 852.02 852.03 852.04 852.05 852.06 852.09 852.10 852.11 852.12 852.13 852.15 852.16 852.19 852.20 852.21 852.22 852.23 852.24 852.25 852.26 852.29 852.30 852.31 852.32 852.35 852.36 852.39 852.40 852.41 852.42 852.43 852.44 852.46 852.49 852.50 852.51 852.52 852.55 852.56 853.00 853.01 853.02 853.03 853.04 853.05 853.06 853.09 853.10 853.11 853.12 853.14 853.15 853.16 853.19 854.00 854.01 854.02 854.03 854.04 854.05 854.06 854.09 854.10 854.11 854.13 854.14 854.15 854.16 854.19 907.0 |
| Crushing injury or internal injury | 860.0 860.1 860.2 860.3 860.4 860.5 861.0 861.01 861.02 861.03 861.1 861.11 861.12 861.13 861.2 861.21 861.22 861.3 861.31 861.32 862.0 862.1 862.21 862.22 862.29 862.31 862.32 862.8 862.9 863.0 863.1 863.20 863.21 863.29 863.30 863.31 863.39 863.40 863.41 863.42 863.43 863.44 863.45 863.46 863.49 863.50 863.51 863.52 863.53 863.54 863.55 863.56 863.59 863.80 863.81 863.82 863.83 863.84 863.85 863.89 863.9 863.90 863.91 863.92 863.93 863.94 863.95 864. 864.0 864.01 864.02 864.03 864.04 864.05 864.09 864.1 864.11 864.12 864.13 864.14 864.15 864.19 865.0 865.01 865.02 865.03 865.04 865.09 865.1 865.11 865.12 865.13 865.14 865.19 866.0 866.00 866.01 866.02 866.03 866.1 866.11 866.12 866.13 867.0 867.1 867.2 867.3 867.4 867.5 867.6 867.7 867.8 867.9 868.00 868.01 868.02 868.03 868.04 868.09 868.10 868.11 868.12 868.13 868.14 868.19 869.0 869.1 900. 900.0 900.01 900.02 900.03 900.1 900.81 900.82 901. 901.0 901.1 901.2 901.3 901.4 901.41 901.42 901.81 901.82 901.83 902. 902.0 902.1 902.11 902.19 902.2 902.21 902.22 902.23 902.24 902.25 902.26 902.27 902.29 902.31 902.32 902.33 902.34 902.39 902.4 902.41 902.42 902.49 902.5 902.51 902.52 902.53 902.54 902.55 902.56 902.59 902.81 902.82 902.87 903. 903.0 903.01 903.02 903.1 903.2 903.3 903.4 903.5 903.8 904.0 904.1 904.2 904.3 904.4 904.41 904.42 904.5 904.51 904.52 904.53 904.54 904.6 904.7 904.8 904.9 906.4 908.0 908.1 908.2 908.3 908.4 925.1 925.2 926. 926.0 926.11 926.12 926.8 927. 927.00 927.01 927.02 927.03 927.09 927.10 927.11 927.20 927.21 927.3 927.8 928. 928.00 928.01 928.10 928.11 928.20 928.21 928.3 928.8 929.0 929.9 |
| Open wounds of head; neck; and trunk | 870.0 870.1 870.2 870.3 870.4 870.8 870.9 871. 871.0 871.1 871.2 871.3 871.4 871.5 871.6 871.7 872. 872.00 872.01 872.02 872.10 872.11 872.12 872.61 872.62 872.63 872.64 872.69 872.71 872.72 872.74 872.79 872.9 873.0 873.1 873.2 873.21 873.22 873.23 873.29 873.3 873.31 873.32 873.33 873.39 873.40 873.41 873.42 873.43 873.44 873.49 873.5 873.51 873.52 873.53 873.54 873.59 873.60 873.61 873.62 873.63 873.64 873.65 873.69 873.70 873.71 873.72 873.73 873.74 873.75 873.79 873.8 873.9 874.00 874.01 874.02 874.1 874.11 874.12 874.2 874.3 874.4 874.5 874.8 874.9 875.0 875.1 876.0 876.1 877.0 877.1 878.0 878.1 878.2 878.3 878.4 878.5 878.6 878.7 878.8 878.9 879.0 879.1 879.2 879.3 879.4 879.5 879.6 879.7 879.8 906.0 |
| Open wounds of extremities | 880.00 880.01 880.02 880.03 880.09 880.10 880.11 880.12 880.13 880.19 880.20 880.21 880.22 880.23 880.29 881.00 881.01 881.02 881.10 881.11 881.12 881.20 881.21 881.22 882.0 882.1 882.2 883.0 883.1 883.2 884.0 884.1 884.2 885.0 885.1 886.0 886.1 887.0 887.1 887.2 887.3 887.4 887.5 887.6 887.7 890.0 890.1 890.2 891.0 891.1 891.2 892.0 892.1 892.2 893.0 893.1 893.2 894.0 894.1 894.2 895.0 895.1 896.0 896.1 896.2 896.3 897. 897.0 897.1 897.2 897.3 897.5 897.6 897.7 905.8 905.9 906.1 |
| Sprains and strains | 840.0 840.1 840.2 840.3 840.4 840.5 840.6 840.7 840.8 840.9 841.0 841.1 841.2 841.3 841.8 841.9 842.0 842.01 842.02 842.09 842.1 842.11 842.12 842.13 842.19 843.0 843.1 843.8 843.9 844.0 844.1 844.2 844.3 844.8 844.9 845.0 845.01 845.02 845.03 845.09 845.10 845.11 845.12 845.13 845.19 846.0 846.1 846.2 846.8 846.9 847.0 847.1 847.2 847.3 847.4 847.9 848.0 848.1 848.2 848.3 848.4 848.41 848.42 848.49 848.5 848.8 848.9 905.7 |
| Superficial injury; contusion | 906.2 906.3 910.0 910.1 910.2 910.3 910.4 910.5 910.6 910.7 910.8 910.9 911.0 911.1 911.2 911.3 911.4 911.5 911.6 911.7 911.8 911.9 912.0 912.1 912.2 912.3 912.4 912.5 912.6 912.7 912.8 912.9 913.0 913.1 913.2 913.3 913.4 913.5 913.6 913.7 913.8 913.9 914.0 914.1 914.2 914.3 914.4 914.5 914.6 914.7 914.8 914.9 915.0 915.1 915.2 915.3 915.4 915.5 915.6 915.7 915.8 915.9 916.0 916.1 916.2 916.3 916.4 916.5 916.6 916.7 916.8 916.9 917.0 917.1 917.2 917.3 917.4 917.5 917.6 917.7 917.8 917.9 918.0 918.1 918.2 918.9 919.0 919.1 919.2 919.3 919.4 919.5 919.6 919.7 919.8 919.9 920. 921.0 921.1 921.2 921.3 921.9 922. 922.0 922.1 922.2 922.3 922.32 922.33 922.4 922.8 923. 923.00 923.01 923.02 923.03 923.09 923.10 923.11 923.20 923.21 923.3 923.8 924.00 924.01 924.10 924.11 924.20 924.21 924.3 924.4 924.5 924.8 924.9 |
| Burns | 906.5 906.6 906.7 906.8 906.9 940.0 940.1 940.2 940.3 940.4 940.5 940.9 941.00 941.01 941.02 941.03 941.04 941.05 941.06 941.07 941.08 941.09 941.10 941.11 941.12 941.13 941.14 941.15 941.16 941.17 941.18 941.19 941.20 941.21 941.22 941.23 941.24 941.25 941.26 941.27 941.28 941.29 941.30 941.31 941.32 941.33 941.34 941.35 941.36 941.37 941.38 941.39 941.40 941.41 941.42 941.45 941.46 941.47 941.48 941.49 941.51 941.54 941.55 941.56 941.58 941.59 942.00 942.01 942.02 942.03 942.04 942.05 942.09 942.10 942.11 942.12 942.13 942.14 942.15 942.19 942.20 942.21 942.22 942.23 942.24 942.25 942.29 942.30 942.31 942.32 942.33 942.34 942.35 942.39 942.40 942.41 942.42 942.43 942.44 942.45 942.49 942.51 942.52 942.53 942.54 942.55 942.59 943.00 943.01 943.02 943.03 943.04 943.05 943.06 943.09 943.10 943.11 943.12 943.13 943.14 943.15 943.16 943.19 943.20 943.21 943.22 943.23 943.24 943.25 943.26 943.29 943.30 943.31 943.32 943.33 943.34 943.35 943.36 943.39 943.40 943.41 943.42 943.43 943.44 943.45 943.46 943.49 943.50 943.51 943.52 943.53 943.54 943.55 943.56 943.59 944.00 944.01 944.02 944.03 944.04 944.05 944.06 944.07 944.08 944.10 944.11 944.12 944.13 944.14 944.15 944.16 944.17 944.18 944.20 944.21 944.22 944.23 944.24 944.25 944.26 944.27 944.28 944.30 944.31 944.32 944.33 944.34 944.35 944.36 944.37 944.38 944.40 944.41 944.42 944.43 944.44 944.45 944.46 944.47 944.48 944.50 944.51 944.52 944.53 944.54 944.56 944.57 944.58 945.00 945.01 945.02 945.03 945.04 945.05 945.06 945.09 945.10 945.11 945.12 945.13 945.14 945.15 945.16 945.19 945.20 945.21 945.22 945.23 945.24 945.25 945.26 945.29 945.30 945.31 945.32 945.33 945.34 945.35 945.36 945.39 945.40 945.41 945.42 945.43 945.44 945.45 945.46 945.49 945.50 945.51 945.52 945.53 945.54 945.55 945.56 945.59 946.0 946.1 946.2 946.3 946.4 946.5 947. 947.0 947.1 947.2 947.3 947.4 947.8 948.00 948.10 948.11 948.20 948.21 948.22 948.30 948.31 948.32 948.33 948.40 948.41 948.42 948.43 948.44 948.50 948.51 948.52 948.53 948.54 948.55 948.60 948.61 948.62 948.63 948.64 948.65 948.66 948.70 948.71 948.72 948.75 948.76 948.77 948.80 948.83 948.87 948.88 948.90 948.91 948.93 948.94 948.97 948.98 948.99 949.0 949.1 949.2 949.3 949.4 949.5 |
| Vasovagal attack | 780.2 |
| Fever of unknown origin | 780.60 780.61 |
| Lymphadenitis | 289.1 289.2 289.3 683. 785.6 |
| Gangrene | 440.24 785.4 |
| Shock | 785.50 785.59 |
| Cardiogenic shock | 785.51 |
| Septic shock | 785.52 |
| Nausea and vomiting | 787.0 787.02 787.03 787.04 |
| Abdominal pain | 789. 789.0 789.00 789.01 789.02 789.03 789.04 789.05 789.06 789.07 789.09 789.6 789.61 789.62 789.63 789.64 789.65 789.66 789.67 789.69 |
| Malaise and fatigue | 780.71 780.79 |
| Other atopic dermatitis and related conditions | 691.8 |
| Fixed drug eruption | 693.0 |
| Idiopathic urticaria | 708.1 |
| Schnitzler syndrome | 708.8 |
| Allergic reactions-Skin | 477.1 518.6 558.3 691.0 692.0 692.1 692.2 692.3 692.4 692.5 692.6 692.70 692.71 692.72 692.73 692.74 692.79 692.8 692.81 692.82 692.83 692.84 692.89 692.9 693. 693.1 693.8 708. 708.0 708.2 708.3 708.4 708.5 708.9 995.0 995.3 |
| Anaphylaxis | 995.60 995.62 995.63 995.64 995.66 995.67 995.69 |
| Allergic reactions-other | 995.7 V07.1 V14.3 V14.4 V14.5 V14.7 V14.8 V14.9 V15.02 V15.05 V15.06 V15.07 V15.09 V72.7 |
| Personal history of allergy to analgesic agent | V14.6 |
| Personal history of allergy to other antibiotic agent | V14.0 V14.1 |
| Personal history of allergy to sulfonamides | V14.2 |
| Hx-eggs allergy | 995.68 V15.03 |
| Hx-radiogrphc dye allrgy | V15.08 |
| Hx-peanut allergy | 995.61 V15.01 |
| Hx-seafood allergy | 995.65 V15.04 |
| Factors influencing health care | V57.8 V62.8 V68.8 V76.4 |
| Rehabilitation care; fitting of prostheses; and adjustment of devices | V52.0 V52.1 V52.4 V52.8 V52.9 V53.8 V57.0 V57.1 V57.21 V57.3 V57.4 V57.81 V57.9 V58.82 |
| Administrative/social admission | V20.0 V20.1 V20.2 V20.31 V20.32 V60.0 V60.1 V60.2 V60.3 V60.4 V60.5 V60.6 V60.81 V60.9 V61.01 V61.03 V61.06 V61.07 V61.09 V61.10 V61.11 V61.12 V61.20 V61.21 V61.22 V61.23 V61.29 V61.3 V61.41 V61.42 V61.49 V61.6 V61.7 V61.8 V61.9 V62.0 V62.1 V62.2 V62.3 V62.4 V62.5 V62.6 V62.81 V62.82 V62.83 V62.9 V63.0 V63.1 V63.2 V63.8 V63.9 V65.0 V65.11 V65.19 V65.2 V65.3 V65.40 V65.41 V65.43 V65.44 V65.45 V65.49 V65.5 V65.8 V65.9 V68.1 V68.2 V68.81 V68.9 |
| Medical examination/evaluation | V29.0 V29.1 V29.2 V29.3 V29.9 V68.01 V68.09 V70. V70.0 V70.3 V70.4 V70.5 V70.6 V70.7 V70.8 V71.9 V72.31 V72.32 V72.5 V72.60 V72.61 V72.62 V72.63 V72.69 V72.8 V72.81 V72.82 V72.83 V72.84 V72.86 V72.9 |
| Long-term use antibiotic | V58.62 |
| Encounter for long-term (current) use of anticoagulants | V58.61 |
| Lng use antiplte/thrmbtc | V58.63 V58.66 |
| Long-term anti-inflamtry | V58.64 |
| Long-term use steroids | V58.65 |
| Tachyphylaxis | V58.89 |
| Other aftercare | V51.0 V51.8 V53.90 V54.81 V54.9 V55.8 V58.30 V58.31 V58.32 V58.41 V58.42 V58.43 V58.44 V58.49 V58.67 V58.69 V58.71 V58.72 V58.73 V58.74 V58.75 V58.76 V58.77 V58.78 V58.81 V58.83 V58.9 V66.0 V66.5 V66.6 V66.7 V66.9 V67. V67.00 V67.01 V67.09 V67.51 V67.59 V67.6 |
| BRCA1 Mutation | brca.1 |
| Other screening for suspected conditions (not mental disorders or infe | 795.18 796.77 V28.0 V28.1 V28.2 V28.3 V28.4 V28.5 V28.8 V28.81 V28.82 V28.89 V28.9 V71.81 V71.89 V72.40 V72.41 V76.0 V76.10 V76.11 V76.12 V76.19 V76.2 V76.3 V76.41 V76.42 V76.43 V76.44 V76.45 V76.46 V76.47 V76.50 V76.51 V76.52 V76.81 V76.89 V76.9 V77.0 V77.1 V77.2 V77.3 V77.4 V77.5 V77.7 V77.8 V77.9 V77.91 V78.0 V80.01 V80.09 V80.1 V80.2 V80.3 V81.0 V81.1 V81.2 V81.4 V81.5 V81.6 V82.0 V82.3 V82.4 V82.5 V82.6 V82.79 V82.8 V82.81 V82.9 |
| Screen-gentc dis carrier | V82.71 |
| Screening for rheumatoid arthritis | V82.1 |
| Screening for other rheumatic disorders | V82.2 |
| Screening for cystic fibrosis | V77.6 |
| Screening for other and unspecified deficiency anemia | V78.1 |
| Screening for other hemoglobinopathies | V78.2 V78.3 |
| Screening for other disorders of blood and blood-forming organs | V78.8 V78.9 |
| Bone marrow replaced by transplant | V42.81 |
| Peripheral stem cells replaced by transplant | V42.82 |
| Pancreas replaced by transplant | V42.83 |
| Trnspl status-intestines | V42.84 |
| Organ replaced by transplant | V42.89 V42.9 |
| Fam hx MEN syndrome | V18.11 |
| Family history of other blood disorders | V18.3 |
| Family history of anemia | V18.2 |
| Fam hx-polycystic kidney | V18.61 |
| Fam hx genet dis carrier | V18.9 |
| Family history of congenital anomalies | V19.5 |
| Poisoning | E980.0 E980.1 E980.2 E980.3 E980.4 E980.5 |
| Electrolytic, caloric, and water-balance agents causing adverse effect | E944.5 |
| Cardiac rhythm regulators causing adverse effects in therapeutic use | E942.0 |
| Other vasodilators causing adverse effects in therapeutic use | E942.4 E942.5 |
| Other antihypertensive agents causing adverse effects in therapeutic u | E942.6 |
| Smooth muscle relaxants causing adverse effects in therapeutic use | E945.1 |
| Cardiotonic glycosides and drugs of similar action causing adverse eff | E942.1 |
| Salicylates causing adverse effects in therapeutic use | E935.3 |
| Other diuretics causing adverse effects in therapeutic use | E944.3 E944.4 |
| Idio hypersom-no lng slp | 327.11 327.12 |
| Hypersomnia | 327.13 327.14 327.19 |
| Insomnia | 302.0 327.00 327.01 327.09 327.10 |
| Sleep apnea | 327.20 327.23 |
| Prim central sleep apnea | 327.21 |
| Idiopath sleep hypovent | 327.24 |
| Recurrnt sleep paralysis | 327.43 |
| Hypothrm-wo low env tmp | 780.65 |
| Flushing | 782.62 |
| Elevated sedimentation rate | 790.1 790.95 |
| Other abnormal blood chemistry | 790.6 790.9 |
| Abnormal or prolonged prothrombin time | 790.92 |
| Reticulocytosis | 790.99 |
| Mitochondrial antibodies positive | 796.9 |
| Sudden infant death syndrome | 798.0 |
| Sulfonamides causing adverse effects in therapeutic use | E931.0 |
| Antibiotics causing adverse effects in therapeutic use | E930. |
| Cephalosporin group causing adverse effects in therapeutic use | E930.5 |
| Penicillins causing adverse effects in therapeutic use | E930.0 |
| Erythromycin and other macrolides causing adverse effects in therapeut | E930.3 |
| Fibrinolysis-affecting drugs causing adverse effects in therapeutic us | E934.4 |
| Adv eff vitamin k | E934.3 |
| Anticoagulants causing adverse effects in therapeutic use | E934.2 E934.5 |
| Natural blood and blood products causing adverse effects in therapeuti | E934.7 |
| Other agents affecting blood constituents causing adverse effects in t | E934.8 |
| Residual codes; unclassified; all E codes | 327.22 327.25 327.26 327.27 327.29 327.40 327.41 327.42 327.49 327.51 327.59 327.8 780.02 780.1 780.50 780.51 780.52 780.53 780.54 780.55 780.56 780.57 780.58 780.59 780.64 780.93 780.94 780.95 780.96 780.97 780.99 781.5 781.6 782.3 782.61 782.8 782.9 783.0 783.6 784.2 790.91 790.93 790.94 793.2 793.99 794.9 795.4 795.81 795.82 795.89 796.3 796.4 796.5 796.6 798.1 798.2 798.9 799.21 799.22 799.23 799.24 799.25 799.29 799.3 799.8 799.81 799.82 799.89 799.9 E000.0 E000.1 E000.8 E000.9 E001.0 E001.1 E002.0 E002.1 E002.2 E002.5 E002.6 E002.7 E002.9 E003.0 E003.1 E003.2 E003.9 E004.0 E004.2 E004.9 E005.0 E005.2 E005.3 E005.4 E005.9 E006.0 E006.1 E006.2 E006.3 E006.4 E006.6 E006.9 E007.0 E007.1 E007.2 E007.3 E007.4 E007.5 E007.6 E007.7 E007.8 E007.9 E008.0 E008.1 E008.2 E008.4 E008.9 E009.0 E009.2 E010.0 E010.1 E010.2 E012.2 E012.9 E013.0 E013.2 E013.4 E013.8 E013.9 E014.1 E014.9 E015.0 E015.2 E015.9 E016.0 E016.1 E016.2 E016.9 E019.0 E019.9 E029.2 E029.9 E030. E800.2 E800.8 E800.9 E801.2 E801.8 E802.0 E804.0 E804.1 E804.8 E804.9 E805.0 E805.2 E805.8 E806.0 E806.2 E806.9 E807.1 E807.2 E807.8 E807.9 E810. E810.0 E810.1 E810.2 E810.8 E811.0 E811.1 E811.2 E811.6 E811.9 E812.0 E812.1 E812.2 E812.3 E812.4 E812.5 E812.6 E812.7 E812.8 E812.9 E813.0 E813.1 E813.2 E813.3 E813.5 E813.6 E813.7 E813.8 E813.9 E814.0 E814.1 E814.2 E814.3 E814.4 E814.6 E814.7 E814.8 E814.9 E815.0 E815.1 E815.2 E815.3 E815.4 E815.5 E815.6 E815.7 E815.8 E815.9 E816.0 E816.1 E816.2 E816.3 E816.4 E816.6 E816.7 E816.8 E816.9 E817.0 E817.1 E817.2 E817.3 E817.7 E817.8 E817.9 E818.0 E818.1 E818.2 E818.3 E818.6 E818.7 E818.8 E818.9 E819.0 E819.1 E819.2 E819.3 E819.4 E819.5 E819.6 E819.7 E819.8 E819.9 E820.0 E820.1 E820.3 E820.9 E821.0 E821.1 E821.2 E821.3 E821.4 E821.5 E821.6 E821.7 E821.8 E821.9 E822.0 E822.1 E822.2 E822.5 E822.6 E822.7 E822.8 E822.9 E823.0 E823.1 E823.2 E823.3 E823.4 E823.6 E823.7 E823.8 E823.9 E824.0 E824.1 E824.2 E824.5 E824.6 E824.7 E824.8 E824.9 E825.0 E825.1 E825.2 E825.3 E825.5 E825.6 E825.7 E825.8 E825.9 E826.0 E826.1 E826.2 E826.4 E826.8 E826.9 E827.0 E827.2 E827.3 E827.8 E827.9 E828.0 E828.2 E828.8 E828.9 E829.0 E829.4 E829.8 E829.9 E830.1 E830.2 E831.0 E831.1 E831.2 E831.3 E831.4 E831.5 E831.8 E831.9 E832.0 E832.1 E832.2 E832.3 E832.4 E832.6 E832.8 E832.9 E833.0 E834.0 E834.1 E834.2 E834.3 E834.4 E834.8 E834.9 E835.0 E835.1 E835.2 E835.3 E835.4 E835.8 E835.9 E836.1 E836.2 E836.5 E836.8 E837.1 E837.3 E837.8 E837.9 E838. E838.0 E838.1 E838.2 E838.3 E838.4 E838.5 E838.6 E838.8 E840.1 E840.3 E840.5 E841.0 E841.1 E841.3 E841.5 E841.9 E842.6 E842.9 E843.1 E843.5 E843.7 E843.9 E844.0 E844.1 E844.2 E844.4 E844.5 E844.6 E844.7 E844.9 E845.0 E846. E847. E848. E849.0 E849.1 E849.2 E849.3 E849.4 E849.5 E849.6 E849.7 E849.8 E849.9 E850.0 E850.1 E850.2 E850.3 E850.4 E850.5 E850.6 E850.7 E850.8 E850.9 E851. E852.0 E852.2 E852.8 E852.9 E853.0 E853.1 E853.2 E853.8 E853.9 E854. E854.0 E854.1 E854.2 E854.3 E855.0 E855.1 E855.2 E855.3 E855.4 E855.5 E855.6 E855.9 E856. E857. E858.0 E858.1 E858.2 E858.3 E858.4 E858.5 E858.6 E858.7 E858.8 E858.9 E860.0 E860.1 E860.2 E860.3 E860.8 E860.9 E861.0 E861.1 E861.2 E861.3 E861.4 E861.6 E861.9 E862.0 E862.1 E862.2 E862.4 E862.9 E863.0 E863.1 E863.4 E863.5 E863.7 E864.0 E864.1 E864.2 E864.3 E864.4 E865.2 E865.3 E865.4 E865.5 E865.8 E865.9 E866.0 E866.1 E866.2 E866.3 E866.4 E866.6 E866.7 E866.8 E866.9 E867. E868.0 E868.1 E868.2 E868.3 E868.8 E868.9 E869.0 E869.1 E869.2 E869.3 E869.4 E869.8 E869.9 E870.0 E870.1 E870.2 E870.3 E870.4 E870.5 E870.6 E870.7 E870.8 E870.9 E871.0 E871.1 E871.3 E871.4 E871.5 E871.6 E871.7 E871.8 E871.9 E872. E872.1 E872.2 E872.9 E873.1 E873.3 E873.5 E873.6 E873.8 E874.0 E874.1 E874.2 E874.3 E874.4 E874.5 E874.8 E876.1 E876.2 E876.3 E876.4 E876.7 E876.8 E876.9 E878.0 E878.1 E878.2 E878.3 E878.4 E878.5 E878.6 E878.8 E878.9 E879.0 E879.1 E879.2 E879.3 E879.4 E879.5 E879.6 E879.7 E879.8 E879.9 E880.0 E880.1 E880.9 E881.0 E881.1 E882. E883.0 E883.1 E883.2 E883.9 E884.0 E884.1 E884.2 E884.3 E884.4 E884.5 E884.6 E884.9 E885.0 E885.1 E885.2 E885.3 E885.4 E885.9 E886.0 E886.9 E888.0 E888.1 E888.8 E888.9 E890.0 E890.1 E890.2 E890.3 E890.8 E890.9 E891.0 E891.1 E891.2 E891.3 E891.8 E891.9 E892. E893.0 E893.1 E893.2 E893.8 E893.9 E894. E895. E896. E897. E898.0 E898.1 E899. E900.0 E900.1 E900.9 E901.0 E901.1 E901.8 E902.0 E902.1 E902.2 E902.8 E902.9 E903. E904.0 E904.1 E904.2 E904.3 E904.9 E905.0 E905.1 E905.2 E905.3 E905.4 E905.5 E905.6 E905.7 E905.8 E905.9 E906.0 E906.1 E906.2 E906.3 E906.4 E906.5 E906.8 E906.9 E907. E908. E908.0 E908.1 E908.2 E908.4 E908.8 E909.1 E909.2 E909.3 E910.0 E910.1 E910.2 E910.3 E910.4 E910.8 E910.9 E911. E912. E913.0 E913.3 E913.8 E913.9 E914. E915. E916. E917.0 E917.1 E917.2 E917.3 E917.4 E917.5 E917.6 E917.7 E917.8 E917.9 E918. E919. E919.0 E919.1 E919.2 E919.3 E919.4 E919.5 E919.6 E919.7 E919.8 E920.0 E920.1 E920.2 E920.3 E920.4 E920.5 E920.8 E920.9 E921. E921.0 E921.1 E921.8 E922.0 E922.1 E922.2 E922.3 E922.4 E922.5 E922.8 E922.9 E923.0 E923.1 E923.2 E923.8 E923.9 E924.0 E924.1 E924.2 E924.8 E924.9 E925. E925.0 E925.1 E925.2 E925.8 E926. E926.0 E926.1 E926.2 E926.3 E926.5 E926.8 E927.0 E927.1 E927.2 E927.3 E927.8 E927.9 E928.0 E928.2 E928.3 E928.4 E928.5 E928.6 E928.7 E928.8 E928.9 E929.0 E929.1 E929.2 E929.3 E929.4 E929.5 E929.8 E929.9 E930.1 E930.4 E930.6 E930.7 E930.8 E931.3 E931.4 E931.5 E931.6 E931.7 E931.8 E931.9 E932.0 E932.1 E932.2 E932.3 E932.4 E932.5 E932.7 E932.8 E932.9 E933.0 E933.1 E933.2 E933.3 E933.4 E933.5 E933.6 E933.7 E933.8 E934.0 E934.1 E934.6 E935.0 E935.1 E935.2 E935.4 E935.6 E935.7 E935.8 E935.9 E936.0 E936.1 E936.2 E936.3 E936.4 E937.0 E937.1 E937.8 E937.9 E938.0 E938.2 E938.3 E938.4 E938.5 E938.6 E938.7 E938.9 E939.0 E939.1 E939.2 E939.3 E939.4 E939.5 E939.6 E939.7 E939.8 E939.9 E940. E940.1 E940.8 E941.0 E941.1 E941.2 E941.3 E941.9 E942.2 E942.3 E942.9 E943. E943.0 E943.1 E943.2 E943.3 E943.4 E943.5 E943.8 E944.1 E944.2 E944.7 E945.0 E945.2 E945.3 E945.4 E945.5 E945.7 E945.8 E946.0 E946.1 E946.2 E946.3 E946.4 E946.5 E946.6 E946.8 E946.9 E947. E947.0 E947.1 E947.3 E947.4 E947.9 E948.4 E948.5 E948.6 E948.8 E949.0 E949.4 E949.5 E949.6 E949.9 E960.0 E960.1 E961. E962.0 E962.1 E962.2 E963. E964. E965.0 E965.1 E965.2 E965.3 E965.4 E965.5 E965.6 E965.8 E965.9 E966. E967.0 E967.1 E967.2 E967.3 E967.4 E967.5 E967.6 E967.7 E967.8 E967.9 E968.0 E968.1 E968.2 E968.3 E968.4 E968.5 E968.6 E968.7 E968.8 E968.9 E969. E970. E972. E973. E975. E976. E977. E979.2 E979.8 E980.6 E980.7 E980.8 E980.9 E981.1 E981.8 E982.0 E982.1 E982.8 E982.9 E983.0 E983.8 E984. E985.0 E985.1 E985.2 E985.4 E985.5 E985.6 E985.7 E986. E987.0 E987.1 E987.2 E987.9 E988. E988.0 E988.1 E988.2 E988.3 E988.4 E988.5 E988.7 E988.9 E989. E990.9 E992.0 E992.9 E995.4 E997.2 E999.0 E999.1 V07.0 V07.2 V07.3 V07.31 V07.51 V07.52 V07.59 V07.8 V07.9 V13.1 V13.9 V15.21 V15.29 V15.3 V15.81 V15.84 V15.85 V15.86 V15.87 V15.9 V16. V16.0 V16.1 V16.2 V16.3 V16.4 V16.41 V16.42 V16.43 V16.49 V16.51 V16.52 V16.59 V16.6 V16.7 V16.8 V17.0 V17.1 V17.2 V17.3 V17.41 V17.49 V17.5 V17.6 V17.7 V17.81 V17.89 V18.0 V18.19 V18.4 V18.51 V18.59 V18.69 V18.7 V18.8 V19.0 V19.2 V19.3 V19.4 V19.6 V19.7 V19.8 V21.0 V21.1 V21.2 V21.8 V21.9 V41.8 V41.9 V43.8 V43.81 V43.82 V43.83 V44.7 V44.8 V45.71 V45.72 V45.73 V45.74 V45.75 V45.76 V45.77 V45.78 V45.79 V45.83 V45.84 V45.86 V45.87 V45.88 V45.89 V46.0 V46.3 V46.8 V46.9 V47.0 V47.1 V47.2 V47.9 V48.0 V48.8 V48.9 V49.81 V49.82 V49.83 V49.84 V49.89 V49.9 V50.0 V50.1 V50.3 V50.41 V50.42 V50.49 V50.8 V50.9 V59.01 V59.02 V59.09 V59.1 V59.2 V59.3 V59.4 V59.5 V59.6 V59.71 V59.72 V59.8 V59.9 V61.5 V64.00 V64.01 V64.03 V64.05 V64.06 V64.09 V64.1 V64.2 V64.3 V64.41 V64.42 V64.43 V69.0 V69.1 V69.2 V69.3 V69.4 V69.5 V69.8 V69.9 V83.89 V84.01 V84.02 V84.03 V84.04 V84.09 V84.81 V84.89 V85.1 V85.52 V86.0 V86.1 V87.09 V87.11 V87.2 V87.31 V87.32 V87.39 V87.41 V87.42 V87.43 V87.45 V87.46 V87.49 V88.01 V88.02 V88.03 V89.01 V89.02 V89.03 V89.09 |
| Hemophilia A carrier | V83.01 V83.02 |
| Cystic fibrosis gene car | V83.81 |
| Infectious and parasitic diseases | 032.8 |
| Infectious and parasitic diseases | 036.8 093.8 094.8 098.8 100.8 |
| Tuberculosis | 010.00 010.01 010.02 010.03 010.04 010.05 010.06 010.80 010.82 010.85 010.86 010.90 010.91 010.92 010.93 |
| TB-Lung | 011.0 011.02 011.03 011.04 011.05 011.06 011.1 011.13 011.15 011.16 011.2 011.21 011.22 011.23 011.24 011.25 011.26 011.3 011.4 011.45 011.46 011.5 011.55 011.56 011.60 011.61 011.62 011.63 011.64 011.66 011.7 011.72 011.73 011.80 011.81 011.82 011.83 011.84 011.85 011.86 011.9 011.91 011.92 011.93 011.94 011.95 011.96 012.0 012.01 012.02 012.03 012.04 012.05 012.06 012.1 012.12 012.13 |
| TB-larynx | 012.3 012.30 012.33 012.36 012.8 012.82 012.83 |
| Tuberculous-CNS | 013.0 013.02 013.03 013.05 013.06 013.1 013.11 013.12 013.16 013.2 013.23 013.26 013.30 013.4 013.6 013.80 013.83 013.9 013.93 |
| TB-GI | 014.00 014.01 014.02 014.03 014.04 014.05 014.80 014.83 014.84 014.85 014.86 |
| TB-Bone | 015.0 015.01 015.02 015.03 015.04 015.06 015.10 015.20 015.24 015.25 015.5 015.54 015.70 015.73 |
| Tuberculosis-Joint | 015.80 015.82 015.84 015.85 015.9 015.90 015.91 015.96 |
| Tuberculosis-Kidney | 016.0 016.04 |
| Tuberculosis-Other organs | 016.1 016.14 016.30 016.35 016.40 016.5 016.50 016.60 016.90 017.0 017.01 017.02 017.04 017.06 017.1 017.2 017.22 017.25 017.26 017.3 017.32 017.6 017.9 017.90 017.91 017.93 018. 018.0 018.01 018.03 018.8 018.81 018.82 018.83 018.91 018.92 018.93 018.96 137.0 137.1 137.2 137.3 137.4 V12.01 |
| Strep/staph septicemia | 038. 038.0 038.1 038.11 038.12 038.19 |
| Other gram negative septicemia | 038.4 038.40 038.41 038.43 038.44 |
| Other specified septicemia | 003.1 020.2 022.3 036.2 038.2 038.3 038.42 054.5 |
| Unspecified septicemia | 038.8 038.9 771.81 790.7 |
| Septic arterial embolism | 449. |
| Syphilis | 090. 090.0 090.1 090.2 090.3 090.4 090.49 090.5 090.6 090.7 091.0 091.1 091.2 091.3 091.4 091.5 091.51 091.52 091.62 091.7 091.81 091.82 091.9 092. 092.0 093. 093.0 093.1 093.21 093.22 093.24 093.81 094. 094.0 094.1 094.2 094.3 094.82 094.84 094.85 094.86 095.0 095.1 095.2 095.3 095.4 095.5 095.6 095.7 095.8 095.9 096. 097.0 097.1 097.9 |
| Sexually transmitted infections-Gonococcus | 098. 098.0 098.1 098.12 098.13 098.15 098.16 098.17 098.19 098.2 098.35 098.37 098.40 098.49 098.50 098.53 098.59 098.6 098.7 098.83 098.84 098.86 098.89 |
| Sexually transmitted infections (not HIV or hepatitis) | 099.0 099.1 099.2 |
| Reiter's disease | 099.3 |
| Unspecified other nongonococcal urethritis [NGU] | 099.40 |
| Sexually transmitted infections (not HIV or hepatitis) | 099.41 099.49 099.50 099.51 099.53 099.54 099.55 099.56 099.59 099.8 099.9 795.05 795.15 795.19 796.79 |
| Other bacterial infections | 020. 020.0 020.8 021.8 023. 023.0 023.1 023.2 023.8 024. 025. 026.0 026.9 027.0 027.1 027.2 027.8 027.9 030. 030.0 030.1 030.2 030.3 031.2 031.8 031.9 033. 033.0 033.1 033.8 |
| Scarlet fever | 034.1 |
| Waterhouse-Friderichsen syndrome, meningococcal | 036.3 |
| Toxic shock syndrome | 040.82 |
| Strep infections | 041.0 041.01 041.02 041.03 041.04 041.05 041.09 |
| Staph infections | 040.89 041.1 041.11 041.12 041.19 |
| Pneumococcus infection in conditions classified elsewhere and of unspe | 041.2 |
| Other bacterial infections | 036.81 037. 039.2 039.3 039.4 039.8 039.9 040.0 040.1 040.2 040.3 040.42 040.81 041.3 041.5 041.6 041.7 041.8 041.81 041.82 041.83 041.84 041.85 041.86 041.9 390. 392.9 795.31 795.39 |
| Other restant bacterial infections | V09.0 V09.1 V09.2 V09.3 V09.4 V09.50 V09.6 V09.70 V09.80 V09.81 V09.90 V09.91 |
| Infection | infect.1 |
| Candidiasis of the mouth (thrush) | 112. 112.0 |
| Other mycoses | 110. 110.0 110.1 110.2 110.3 110.4 110.5 110.6 110.8 110.9 111.0 111.1 111.2 111.3 111.8 111.9 112.1 112.2 112.3 112.5 112.82 112.84 112.85 112.89 114.1 114.3 115.00 115.09 115.1 115.10 115.19 115.90 115.99 116.0 116.1 116.2 117.0 117.1 117.2 117.3 117.4 117.5 117.6 117.7 117.8 117.9 118. |
| HIV infection | 042. 079.53 279.10 279.19 795.71 V08. |
| Hepatitis | 070. 070.0 070.1 070.20 070.21 070.22 070.23 070.3 070.30 070.31 070.32 070.33 070.4 070.41 070.42 070.43 070.44 070.5 070.51 070.52 070.53 070.54 070.6 070.7 070.70 070.71 070.9 571.4 571.41 571.42 571.49 573.1 573.2 573.3 |
| Other viral infections | 054.7 055.7 056.7 |
| Other viral infections | 053.1 053.10 053.11 053.12 053.13 053.14 053.19 053.7 053.71 053.8 053.9 |
| Herpes simplex infection | 054. 054.0 054.1 054.11 054.12 054.13 054.19 054.2 054.6 054.71 054.73 054.74 054.8 054.9 058.1 058.11 058.81 058.82 058.89 |
| Other and unspecified viral infection | 050. 050.1 051.01 051.1 051.2 052.7 052.8 052.9 055.9 056. 056.9 057.0 057.8 057.9 059.10 059.8 060. 060.1 061. 065. 065.0 065.4 066.0 066.1 066.3 066.4 066.41 071. 072. 072.0 072.3 072.8 072.9 074.0 074.1 074.3 074.8 075. 078.0 078.1 078.10 078.11 078.12 078.19 078.2 078.3 078.4 078.5 078.6 078.7 078.81 078.82 078.88 078.89 079.0 079.1 079.2 079.3 079.4 079.5 079.51 079.52 079.59 079.6 079.81 079.82 079.83 079.88 079.89 079.98 079.99 790.8 |
| Other infections; including parasitic | 080. 081.0 081.1 081.2 081.9 082. 082.0 082.1 082.2 082.4 082.41 082.49 082.8 083.1 083.2 083.8 083.9 084. 084.0 084.1 084.2 084.3 084.4 084.5 084.8 084.9 085. 085.0 085.1 085.2 085.4 086. 086.0 086.2 086.4 086.5 087. 087.0 087.1 088.0 088.8 088.81 088.82 088.9 100. 100.0 100.89 101. 102. 102.0 102.1 102.2 102.3 102.4 102.5 102.6 102.7 102.8 103. 103.0 103.1 103.2 103.3 104.0 104.8 104.9 120.0 120.1 120.2 120.3 120.8 120.9 121.0 121.1 121.2 121.3 121.4 121.5 121.6 121.8 121.9 122. 122.0 122.1 122.2 122.3 122.4 122.5 122.6 122.7 122.8 122.9 123.0 123.1 123.3 123.4 123.5 123.6 123.8 123.9 124. 125.0 125.1 125.2 125.3 125.4 125.5 125.6 125.7 125.9 126. 126.0 126.1 126.2 126.3 126.8 127.0 127.1 127.2 127.3 127.4 127.5 127.6 127.7 127.8 127.9 128.0 128.1 128.8 129. 130.5 130.7 130.8 131. 131.0 131.01 131.02 131.09 131.8 132.0 132.1 132.2 132.3 132.9 133. 133.0 133.8 134.0 134.1 134.2 134.8 134.9 135. 136.0 136.1 136.21 136.4 136.5 136.8 136.9 V12.00 V12.03 V12.04 V12.09 |
| Immunizations and screening for infectious disease | 795.51 795.6 V01. V01.0 V01.1 V01.2 V01.3 V01.4 V01.5 V01.6 V01.7 V01.71 V01.81 V01.82 V01.84 V01.89 V02.0 V02.1 V02.2 V02.3 V02.4 V02.5 V02.51 V02.52 V02.53 V02.60 V02.61 V02.62 V02.69 V02.7 V02.8 V02.9 V03.0 V03.1 V03.2 V03.3 V03.4 V03.5 V03.6 V03.7 V03.81 V03.82 V03.89 V03.9 V04.0 V04.1 V04.2 V04.3 V04.4 V04.5 V04.6 V04.7 V04.81 V04.82 V04.89 V05.0 V05.1 V05.2 V05.3 V05.4 V05.8 V05.9 V06.0 V06.1 V06.2 V06.3 V06.4 V06.5 V06.6 V06.8 V06.9 V28.6 V71.2 V71.82 V71.83 |
| Immunizations and screening for infectious disease | V73.0 V73.1 V73.2 V73.3 V73.4 V73.5 V73.6 V73.81 V73.88 V73.89 V73.9 V73.98 V74.0 V74.1 V74.2 V74.3 V74.4 V74.5 V74.6 V74.8 V74.9 V75.0 V75.1 V75.2 V75.3 V75.4 V75.5 V75.6 V75.7 V75.8 V75.9 |
| Thyrotoxicosis with or without goiter | 242. |
| Thyrotoxicosis with or without goiter | 242.0 242.00 242.01 242.1 242.10 242.11 242.2 242.20 242.21 242.3 242.30 242.31 242.8 242.80 242.81 242.9 242.90 242.91 |
| Thyrotoxicosis with or without goiter | 242.40 242.41 |
| Other thyroid disorders | 240. 240.0 240.9 241.0 241.1 241.9 |
| Endemic cretinism | 243. |
| Other thyroid disorders | 244.0 244.1 244.3 |
| Iodine hypothyroidism | 244.2 |
| Other specified acquired hypothyroidism | 244.8 |
| Unspecified hypothyroidism | 244.9 |
| Other thyroid disorders | 245. 245.0 245.1 245.2 245.3 245.8 |
| Iatrogenic thyroiditis | 245.4 |
| Other thyroid disorders | 246.0 246.1 246.2 |
| Hemorrhage and infarction of thyroid | 246.3 |
| Other specified disorders of thyroid | 246.8 |
| Unspecified disorder of thyroid | 246.9 |
| Nonspecific abnormal results of function study of thyroid | 794.5 |
| Deficiency of humoral immunity | 279.0 |
| Hypogammaglobulinemia, unspecified | 279.00 |
| Selective IgA immunodeficiency | 279.01 |
| Selective IgM immunodeficiency | 279.02 |
| Selective deficiency of IgG | 279.03 |
| Congenital hypogammaglobulinemia | 279.04 |
| Immunodeficiency with increased IgM | 279.05 |
| Common variable immunodeficiency | 279.06 |
| Thymic hypoplasia | 279.11 |
| Wiskott-Aldrich syndrome | 279.12 |
| Nezelof's syndrome | 279.13 |
| SCID | 279.2 |
| Unspecified immunity deficiency | 279.3 |
| Autoimmune disease, not elsewhere classified | 279.4 |
| Autoimmun lymphprof synd | 279.41 |
| Autoimmune disease NOS | 279.49 |
| Reticuloendothelial blockade | 279.8 |
| Unspecified disorder of immune mechanism | 279.9 |
| Disorders of mineral metabolism | 275. |
| Disorders of iron metabolism | 275.0 |
| Secondary hemochromatosis | 275.03 |
| Iron overload | 275.09 |
| Hepatolenticular degeneration | 275.1 |
| Hypomagnesemia | 275.2 |
| Vitamin D-resistant rickets | 275.3 |
| Disorders of calcium metabolism | 275.4 |
| Metastatic calcification | 275.40 |
| Hypocalcemia | 275.41 |
| Milk alkali syndrome | 275.42 |
| Pseudopseudohypoparathyroidism | 275.49 |
| Hungry bone syndrome | 275.5 |
| Other specified disorders of mineral metabolism | 275.8 |
| Kidney crystallization | 275.9 |
| Obesity | 278.01 V85.30 V85.31 V85.32 V85.33 V85.34 V85.35 V85.36 V85.37 V85.38 V85.39 |
| Obesity hypovent synd | 278.03 |
| Image test incon d/t fat | 793.91 |
| Obesity | 278.00 278.02 V85.21 V85.22 V85.23 V85.24 V85.25 |
| BMI,pediatric >= 95% | V85.54 |
| Disorders of amino-acid transport and metabolism | 270. |
| Other and unspecified metabolic; nutritional; and endocrine disorders | 270.0 270.1 270.2 270.3 270.4 270.5 270.6 270.7 270.8 270.9 271. 271.0 271.1 271.2 |
| Intolerance or malabsorption of lactose | 271.3 |
| Other and unspecified metabolic; nutritional; and endocrine disorders | 271.4 271.8 272.5 272.6 272.7 272.8 272.9 273. 273.0 273.1 273.2 273.3 |
| Alpha-1-antitrypsin deficiency | 273.4 |
| Other disorders of plasma protein metabolism | 273.8 |
| Hereditary hemochromatosis | 275.01 |
| Other and unspecified metabolic; nutritional; and endocrine disorders | 277.1 277.2 |
| Amyloidosis | 277.3 |
| Familial Mediterranean fever | 277.31 |
| Other amyloidosis | 277.39 |
| Other and unspecified metabolic; nutritional; and endocrine disorders | 277.4 277.5 |
| Other deficiencies of circulating enzymes | 277.6 |
| Other and unspecified metabolic; nutritional; and endocrine disorders | 277.7 277.8 277.81 277.82 277.84 277.85 |
| Peroxisomal disorders | 277.86 |
| Myoclonus with Epilepsy and with Ragged Red Fibers (MERRF syndrome) | 277.87 |
| Macroamylasemia | 277.89 |
| Unspecified disorder of metabolism | 277.9 |
| Localized adiposity | 278.1 |
| Hypervitaminosis A | 278.2 |
| Hypercarotinemia | 278.3 |
| Hypervitaminosis D | 278.4 |
| Other hyperalimentation | 278.8 |
| Abnormal weight gain | 783.1 |
| Abnormal loss of weight | 783.21 |
| Underweight | 783.22 |
| Feeding difficulties and mismanagement | 783.3 |
| Lack norm physio dev NOS | 783.40 |
| Failure to thrive | 783.41 |
| Delayed milestones | 783.42 |
| Short stature | 783.43 |
| Polydipsia | 783.5 |
| Failure to thrive-adult | 783.7 |
| Other symptoms concerning nutrition, metabolism, and development | 783.9 |
| Nonspecific abnormal results of function study of basal metabolism | 794.7 |
| Other nonspecific immunological findings | 795.7 |
| Smooth muscle antibodies positive | 795.79 |
| Low BMI | V85.0 V85.51 V85.53 |
| Isulin Pump | V45.85 V53.91 V65.46 |
| DM, type1 | 250.01 250.03 250.13 250.21 250.23 250.31 250.33 250.41 250.43 250.51 250.53 250.61 250.63 250.71 250.73 250.81 250.83 250.91 250.93 |
| DM, Type2 | 249.00 250. 250.00 250.02 250.1 250.10 250.11 250.12 250.2 250.20 250.22 250.3 250.30 250.32 250.4 250.40 250.42 250.50 250.52 250.6 250.60 250.62 250.70 250.72 250.8 250.80 250.82 250.90 250.92 790.21 790.22 790.29 791.5 791.6 |
| Scondary DM | 249.01 249.10 249.11 249.20 249.21 249.40 249.41 249.50 249.51 249.60 249.61 249.71 249.80 249.81 249.90 249.91 |
| Hypoglycemia | 251.0 251.1 251.2 |
| Postsurgical hypoinsulinemia | 251.3 |
| Abnormality of secretion of glucagon | 251.4 |
| Zollinger-Ellison syndrome | 251.5 |
| Other endocrine disorders | 251.8 251.9 |
| Other endocrine disorders | 252.0 252.01 |
| Other hyperparathyroidism | 252.08 |
| Sec hyprprthyrd nonrenal | 252.02 |
| Hypoparathyroidism | 252.1 |
| Other endocrine disorders | 252.8 252.9 |
| Gigantism | 253.0 |
| Pregnancy-related A-G syndrome | 253.1 |
| Sheehan's syndrome | 253.2 |
| Pituitary dwarfism | 253.3 |
| Somatotropin deficiency | 253.4 |
| Vasopressin deficiency | 253.5 |
| Syndrome of inappropriate secretion of antidiuretic hormone | 253.6 |
| Nelson syndrome | 253.7 |
| Pituitary apoplexy | 253.8 |
| Unspecified disorder of the pituitary gland and its hypothalamic contr | 253.9 |
| Diseases of thymus gland | 254. |
| Persistent hyperplasia of thymus | 254.0 |
| Abscess of thymus | 254.1 |
| Other specified diseases of thymus gland | 254.8 |
| Overproduction of cortisol | 255.0 |
| Hyperaldosteronism | 255.1 |
| Glucrtcod-rem aldsternsm | 255.11 |
| Conn's syndrome | 255.12 |
| Bartter's syndrome | 255.13 |
| Secondry aldosternsm NEC | 255.14 |
| Virilization | 255.2 |
| Overproduction of ACTH | 255.3 |
| Corticoadrenal insufficiency | 255.4 |
| Secondary hypocortisolism | 255.41 |
| Mineralcorticoid defcnt | 255.42 |
| Other adrenal hypofunction | 255.5 |
| Medulloadrenal hyperfunction | 255.6 |
| Other specified disorders of adrenal glands | 255.8 |
| Unspecified disorder of adrenal glands | 255.9 |
| Ovarian dysfunction | 256. 256.0 256.1 |
| Ovarian failure | 256.2 256.3 256.8 |
| Polycystic ovaries | 256.4 |
| Testicular dysfunction | 257. 257.0 257.1 257.2 257.8 |
| Mult endo neoplas type I | 258.01 |
| Other combinations of endocrine dysfunction | 258.1 |
| Other specified polyglandular dysfunction | 258.8 |
| Polyglandular dysfunction, unspecified | 258.9 |
| Delay in sexual development and puberty, not elsewhere classified | 259.0 |
| Sexual precocity NOS | 259.1 |
| Carcinoid syndrome | 259.2 |
| Ectopic hormone secretion, not elsewhere classified | 259.3 |
| Dwarfism NEC | 259.4 |
| Androgen insensitvty NOS | 259.50 |
| Complete androgen insensitivity | 259.51 |
| Part androgen insnsitvty | 259.52 |
| Werner's syndrome | 259.8 |
| Unspecified endocrine disorder | 259.9 |
| Nonspecific abnormal results of other endocrine function study | 794.6 |
| Unspecified protein-calorie malnutrition | 263.9 |
| Other malnutrition | 260. 261. 262. 263.0 263.1 263.2 263.8 |
| Vitamin A deficiency | 264.0 264.1 264.2 264.3 264.4 264.5 264.7 264.8 264.9 |
| Beriberi | 265.0 |
| Wernicke's disease | 265.1 |
| Pellagra | 265.2 |
| Ariboflavinosis | 266.0 |
| Vitamin B deficiency | 266.1 266.2 266.9 |
| Scurvy | 267. |
| Rickets | 268.0 268.1 |
| Osteomalacia, unspecified | 268.2 |
| Unspecified vitamin D deficiency | 268.9 |
| Deficiency of vitamin K | 269.0 |
| Vitamin E deficiency | 269.1 |
| Zinc deficiency | 269.3 |
| Wasting disease | 799.4 |
| Other nutritional deficiencies | 269. 269.2 269.9 V12.1 |
| Disorders of lipid metabolism | 272. 272.0 272.1 272.2 272.3 272.4 |
| Gout and other crystal arthropathies | 274. 274.0 274.01 274.02 274.03 274.1 274.11 274.19 274.8 274.81 274.82 |
| Chondrocalcinosis, due to dicalcium phosphate crystals | 712.10 712.13 712.16 712.17 712.19 712.2 712.21 712.22 712.23 712.24 712.25 712.26 712.27 712.28 712.29 |
| Chondrocalcinosis, cause unspecified | 712.30 712.31 712.32 712.33 712.34 712.35 712.36 712.37 712.38 712.39 |
| Other specified crystal arthropathies | 712.8 712.81 712.83 712.86 712.89 |
| Unspecified crystal arthropathy | 712.91 712.92 712.93 712.95 712.96 |
| Crystal arthropathies | 712. |
| Hyposmolality and/or hyponatremia | 276.1 |
| Hypovolemia | 276.51 276.52 |
| Hyperpotassemia | 276.7 |
| K deficiency | 276.8 |
| Na excess | 276.0 |
| Respiratory acidosis/alkalosis | 276.2 276.3 |
| Mixed acid-base balance disorder | 276.4 |
| Other fluid and electrolyte disorders | 276.6 276.9 |
| Fibrocystic disease of the pancreas | 277.0 |
| Cystic fibrosis | 277.00 277.01 277.02 277.03 277.09 |
| Acute posthemorrhagic anemia | 285.1 |
| Sickle cell anemia | 282.41 282.42 282.5 282.60 282.61 282.62 282.63 282.64 282.68 282.69 |
| Iron deficiency anemia | 280.1 280.8 280.9 |
| Other deficiency anemia | 281.0 281.1 |
| Folate-deficiency anemia | 281.2 |
| Other specified megaloblastic anemias, not elsewhere classified | 281.3 |
| Other deficiency anemia | 281.4 281.9 |
| Anemia due to vitamin B>6< deficiency | 281.8 |
| Aplastic anemia | 284.01 284.81 |
| Fanconi's anemia | 284.09 |
| Pancytopenia | 284.1 |
| Transient erythroblastopenia of childhood | 284.89 |
| Medullary hypoplasia | 284.9 |
| Iron deficiency anemias | 280. 280.0 |
| Acquired hemolytic anemia | 283. 283.0 |
| Non-autoimmune hemolytic anemias | 283.1 283.19 |
| Hemolytic-uremic syndrome | 283.11 |
| Marchiafava-Micheli syndrome | 283.2 |
| Neonatal anemia | 283.9 |
| Hereditary hemolytic anemias | 282. |
| Spherocytosis/Elliptocytosis | 282.0 282.1 |
| G-6-PD deficiency anemia | 282.2 |
| Other hemolytic anemias due to enzyme deficiency | 282.3 |
| Thalassemias | 282.4 282.43 282.46 282.49 282.7 |
| Cooley's anemia | 282.44 |
| Other specified hereditary hemolytic anemias | 282.8 |
| Myelophthisis | 284.2 |
| Sideroblastic anemia | 285.0 |
| Deficiency and other anemia | 285.2 285.21 285.22 285.29 |
| Other specified anemia | 285.8 285.9 |
| Coagulation defects | 286. |
| Congenital factor VIII disorder | 286.0 |
| Congenital factor IX disorder | 286.1 |
| Congenital factor XI deficiency | 286.2 |
| Hypoproconvertinemia | 286.3 |
| von Willebrand's disease | 286.4 |
| Purpura, fulminans | 286.6 |
| Acquired coagulation factor deficiency | 286.7 |
| Thrombophilia | 286.9 |
| Protein S deficiency | 289.81 |
| Sec hypercoagulable st | 289.82 |
| Thrombocytopenic purpura | 287.30 |
| Immune thrombocytopenic purpura | 287.31 |
| Evans' syndrome | 287.32 |
| Cong/herid thromb purpra | 287.33 |
| Other primary thrombocytopenia | 287.39 |
| Thrombocytopenia, unspecified | 287.5 |
| Heparin-indu thrombocyto | 289.84 |
| Allergic purpura | 287.0 |
| Thrombocytopathy | 287.1 |
| Purpura, senile | 287.2 |
| Other specified hemorrhagic conditions | 287.8 |
| Unspecified hemorrhagic conditions | 287.9 |
| Spontaneous ecchymoses | 782.7 |
| Diseases of white blood cells | 288. |
| Neutropenia | 288.0 288.09 |
| Congenital neutropenia | 288.01 |
| Cyclic neutropenia | 288.02 |
| Drug induced neutropenia | 288.03 |
| Neutropenia d/t infectn | 288.04 |
| Job's syndrome | 288.1 |
| Hereditary leukomelanopathy | 288.2 |
| Eosinophilia | 288.3 |
| Hemophagocytic syndromes | 288.4 |
| Decreased white blood cell count | 288.5 288.59 |
| Leukocytopenia NOS | 288.50 |
| Lymphocytopenia | 288.51 |
| Leukocytosis NOS | 288.6 288.60 288.69 |
| Lymphocytosis-symptomatc | 288.61 |
| Leukemoid reaction | 288.62 |
| Monocytosis-symptomatic | 288.63 |
| Plasmacytosis | 288.64 |
| Basophilia | 288.65 |
| Bandemia | 288.66 |
| Other specified disease of white blood cells | 288.8 |
| White blood cell abnormality | 288.9 |
| Neutropenic splenomegaly | 289.53 |
| Polycythemia, secondary | 289.0 |
| Hypersplenism | 289.4 |
| Other diseases of spleen | 289.5 289.50 |
| Chronic congestive splenomegaly | 289.51 289.52 |
| Splenic infarction | 289.59 |
| Familial polycythemia | 289.6 |
| Polycythemia | polycy.1 |
| Sulfhemoglobinemia | 289.7 |
| Other hematologic conditions | 289.8 V12.3 |
| Myelofibrosis | 289.83 |
| Pseudocholinesterase deficiency | 289.89 |
| Unspecified diseases of blood and blood-forming organs | 289.9 |
| Abnormality of red blood cells | 790.0 |
| Drop, hematocrit, precip | 790.01 |
| Red blood cell agglutination | 790.09 |
| Blood transfusion, without reported diagnosis | V58.2 |
| Other specified drug-induced mental disorders | 292.8 |
| Adjustment disorders | 309. 309.0 309.1 309.22 309.23 309.29 309.3 309.4 309.8 309.82 309.83 309.89 309.9 |
| Adjustment dis w anxiety | 309.24 309.28 |
| Schizophrenia and other psychotic disorders | 293.81 293.82 295.0 295.01 295.02 295.03 295.04 295.05 295.1 295.10 295.11 295.12 295.13 295.14 295.15 295.2 295.20 295.21 295.22 295.23 295.24 295.25 295.3 295.31 295.32 295.33 295.34 295.35 295.4 295.40 295.41 295.42 295.43 295.44 295.45 295.5 295.51 295.52 295.53 295.54 295.55 295.60 295.61 295.62 295.63 295.64 295.65 295.7 295.70 295.71 295.72 295.73 295.74 295.75 295.8 295.81 295.82 295.83 295.84 295.85 295.9 295.91 295.92 295.93 295.94 295.95 297.0 297.1 297.2 297.3 297.8 297.9 298.0 298.1 298.2 298.3 298.4 298.8 298.9 |
| Alcohol-related disorders | 291.0 291.1 291.2 291.3 291.4 291.5 291.81 291.82 291.89 291.9 303. 303.00 303.01 303.02 303.03 303.9 303.91 303.92 303.93 305. 305.0 305.00 305.01 305.02 305.03 760.71 |
| Alcohol-related disorders | 980. 980.0 |
| Substance-related disorders | 292.0 292.11 292.12 292.2 292.81 292.82 292.83 292.84 292.85 292.89 292.9 304.0 304.01 304.02 304.03 304.10 304.11 304.12 304.13 304.2 304.21 304.22 304.23 304.30 304.31 304.32 304.33 304.4 304.41 304.42 304.43 304.5 304.51 304.53 304.6 304.61 304.62 304.63 304.70 304.71 304.72 304.73 304.80 304.81 304.82 304.83 304.9 304.90 304.91 304.92 304.93 305.20 305.21 305.22 305.23 305.30 305.31 305.32 305.33 305.40 305.41 305.42 305.43 305.50 305.51 305.52 305.53 305.6 305.61 305.62 305.63 305.70 305.71 305.72 305.73 305.80 305.81 305.9 305.91 305.92 305.93 648.3 648.31 648.32 648.33 648.34 655.50 655.51 655.53 760.72 760.73 760.75 779.5 965.00 965.01 965.02 965.09 V65.42 |
| Suicide and intentional self-inflicted injury | E950.0 E950.1 E950.2 E950.3 E950.4 E950.5 E950.6 E950.7 E950.8 E950.9 E951.0 E951.1 E951.8 E952.0 E952.1 E952.8 E953.0 E953.8 E953.9 E954. E955.0 E955.1 E955.2 E955.3 E955.4 E955.5 E955.6 E955.9 E956. E957.0 E957.1 E957.2 E957.9 E958.0 E958.1 E958.3 E958.4 E958.5 E958.6 E958.7 E958.8 E958.9 E959. V62.84 |
| Neuroleptic malignant syndrome | 333.92 |
| Codes related to mental health disorders | V11. V11.0 V11.1 V11.8 V15.41 V15.42 V15.49 V15.82 V66.3 V70.1 V70.2 V71.01 V71.02 V71.09 V79.0 V79.2 V79.3 V79.8 V79.9 |
| Alcoholic cardiomyopathy | 425.5 |
| Alcoholic fatty liver | 571.0 |
| Alcoholic fatty liver | fatliv.1 |
| Alcoholic polyneuropathy | 357.5 |
| Alcoholic gastritis | 535.30 535.31 |
| Laennec's cirrhosis | 571.2 |
| Alcoholic liver damage | 571.1 571.3 |
| Codes related to substance-related disorders | 305.1 790.3 V11.3 V79.1 |
| Miscellaneous mental disorders | 300.8 302.8 |
| Dissociative disorders | 300.12 300.13 300.14 300.15 300.6 |
| Eating disorders | 307.1 307.50 307.51 307.52 307.53 307.54 307.59 |
| Factitious disorders | 300.16 300.19 306.0 306.1 306.2 306.3 306.4 306.50 306.52 306.53 306.59 306.6 306.7 306.8 306.9 |
| Sexual and gender identity disorders | 302.50 302.51 302.52 302.53 |
| Sexual and gender identity disorders | 302.1 302.2 302.3 302.4 302.6 302.7 302.70 302.71 302.72 302.73 302.74 302.75 302.76 302.79 302.81 302.82 302.84 302.85 302.89 306.51 |
| Sleep disorders | 307.40 307.41 307.42 307.43 307.44 307.45 307.46 307.47 307.48 307.49 |
| Somatoform disorders | 300.11 300.7 300.81 300.82 307.80 307.81 307.89 |
| Mental disorders due to general medical conditions not elsewhere class | 293.89 293.9 310.1 |
| Other miscellaneous mental conditions | 316. 648.40 648.41 648.42 648.43 648.44 V40.2 V67.3 |
| Anxiety disorders | 293.84 300.0 300.09 |
| Generalized anxiety disorder | 300.02 |
| Apprehension | 300.00 |
| Anxiety disorders-Panic | 300.01 300.21 |
| Anxiety disorders | 300.10 300.2 300.22 300.23 300.29 300.3 300.5 300.89 300.9 308. 308.0 308.1 308.2 308.3 308.4 309.81 313.0 313.1 313.21 313.22 313.3 313.82 313.83 |
| Conduct disorder | 312.00 312.01 312.02 312.03 312.10 312.11 312.12 312.2 312.21 312.22 312.23 312.4 312.81 312.82 312.89 312.9 |
| Opposition defiant disor | 313.81 |
| Attention deficit disorder and Attention deficit hyperactivity disorde | 314. 314.0 314.00 314.1 314.2 314.8 |
| Delirium, dementia, and amnestic and other cognitive disorders | 290. 290.0 290.10 290.11 290.12 290.13 290.20 290.21 290.3 290.4 290.40 290.41 290.42 290.43 290.8 290.9 293.0 293.1 294.0 294.10 294.11 294.20 294.8 294.9 310.0 310.2 310.9 331. 331.0 331.11 331.19 331.2 331.82 797. |
| Communication disorders | 307.0 307.9 315.31 315.34 315.35 315.39 V40.1 |
| Developmental disabilities | 315.01 315.09 315.32 315.5 315.8 |
| Mild intellect disabilty | 317. 318.0 319. |
| Sev intellect disability | 318.1 318.2 |
| Learning disorders | 315.0 315.1 315.2 315.9 V40. V40.0 |
| Developmental coordination disorder | 315.4 |
| Elimination disorders | 307.6 307.7 |
| Other disorders of infancy childhood or adolescence | 307.3 309.21 313.23 313.89 313.9 |
| Pervasive developmental disorders | 299.0 299.00 299.01 299.10 299.11 299.80 299.81 299.90 299.91 |
| Transient tic disorder | 307.21 |
| Tic disorders | 307.20 307.22 |
| Tourette's disorder | 307.23 |
| Impulse control disorders not elsewhere classified | 312.30 312.31 312.32 312.33 312.34 312.35 312.39 |
| Bipolar disorders | 296.00 296.01 296.02 296.03 296.04 296.05 296.06 296.10 296.11 296.12 296.13 296.14 296.15 296.16 296.40 296.41 296.42 296.43 296.44 296.45 296.46 296.50 296.51 296.52 296.53 296.54 296.55 296.56 296.60 296.61 296.62 296.63 296.64 296.65 296.66 296.7 296.80 296.81 296.82 296.89 296.90 296.99 |
| Depressive disorders | 293.83 296.2 296.20 296.21 296.22 296.23 296.24 296.25 296.26 296.3 296.31 296.32 296.33 296.34 296.35 296.36 300.4 311. |
| Personality disorders | 301.0 301.1 301.11 301.12 301.2 301.21 301.22 301.3 301.4 301.5 301.51 301.59 301.6 301.7 301.8 301.81 301.82 301.83 301.84 301.9 |
| Meningitis (except that caused by TB or STD) | 003.21 |
| Meningitis (except that caused by TB or STD) | 036. 036.0 047.0 047.1 047.8 047.9 049.0 049.1 053. 053.0 054.72 072.1 100.81 112.83 114.2 115.01 115.11 115.91 320.0 320.1 320.2 320.3 320.7 320.8 320.81 320.82 320.9 321.0 321.1 321.2 321.3 321.4 321.8 322. 322.0 322.1 322.2 322.9 |
| Subacute sclerosing panencephalitis | 046.2 |
| von Economo's disease | 049.8 |
| Encephalitis (except that caused by TB or STD) | 036.1 049.9 052. 052.0 054.3 055. 055.0 056.01 058.21 058.29 062.0 062.1 062.2 062.3 062.5 062.8 063. 063.0 063.8 064. 066.2 072.2 130. 130.0 139. 139.0 323.01 323.02 323.1 323.2 323.41 323.42 323.51 323.61 323.62 323.63 323.71 323.72 323.81 323.82 323.9 341.20 341.21 341.22 |
| Poliomyelitis | 045. 045.0 045.00 045.10 045.13 045.2 045.90 138. V12.02 |
| Other CNS infection | 046.0 046.8 046.9 048. 324.0 324.1 324.9 326. |
| Jakob-Creutzfeldt disease | 046.1 046.11 046.19 |
| Fatal familial insomnia | 046.72 |
| Progressive multifocal leukoencephalopathy | 046.3 |
| Parkinsonism | 332.0 |
| Multiple sclerosis | 340. |
| Idio perph auto neur NOS | 337.00 |
| Carotid sinus syndrome | 337.01 |
| Sphenopalatine neuralgia | 337.09 |
| Peripheral autonomic neuropathy in disorders classified elsewhere | 337.1 |
| Autonomic disorders | 337.3 337.9 |
| Rett's disorder | 330.8 |
| Norml pressure hydroceph | 331.5 |
| Reye's syndrome | 331.81 |
| Hydrocephralus | 331.3 331.4 |
| Shy-Drager syndrome | 333.0 |
| Huntington's chorea | 333.4 |
| Tardive dyskinesia | 333.85 |
| Friedreich's ataxia | 334.0 |
| Hereditary spastic paraplegia | 334.1 |
| Primary cerebellar degeneration | 334.2 |
| Roussy-Leavy syndrome | 334.3 |
| Other choreas | 333.5 |
| Stiff-man syndrome | 333.91 |
| Other and unspecified hereditary and degenerative nervous conditions | 330.0 330.1 330.2 330.3 330.9 331.7 331.9 333.1 333.2 333.3 333.6 333.71 333.72 333.79 333.81 333.82 333.83 333.84 333.89 333.90 333.93 333.94 333.99 334. 334.4 334.8 335. 335.0 335.1 335.19 335.2 335.21 335.22 335.23 335.24 335.29 335.8 336.1 336.2 336.3 336.8 336.9 |
| Syringomyelia | 336.0 |
| Amyotrophic lateral sclerosis | 335.20 |
| Kugelberg-Welander disease | 335.11 |
| Hemiplegia | 342.0 342.01 342.02 342.1 342.11 342.12 342.80 342.81 342.82 342.9 342.90 342.91 342.92 |
| Other paralysis | 343. 343.0 343.1 343.2 343.3 343.4 343.8 343.9 344.00 344.01 344.02 344.03 344.04 344.09 344.1 344.2 344.30 344.31 344.32 344.4 344.41 344.42 344.5 344.6 344.60 344.8 344.81 344.89 344.9 780.72 781.4 |
| Epilepsy | 345.0 345.00 345.01 345.1 345.10 345.11 345.2 345.3 345.4 345.40 345.41 345.5 345.50 345.51 345.60 345.61 345.70 345.71 345.80 345.81 345.9 345.90 345.91 |
| Convulsions | 780.3 780.31 780.32 780.39 |
| Cluster headache | 339.01 339.02 |
| Other headache syndromes | 339. |
| Paroxysmal hemicrania | 339.03 339.04 |
| Migraine | 346. 346.00 346.01 346.02 346.03 346.1 346.11 346.12 346.13 346.2 346.20 346.21 346.22 346.30 346.40 346.41 346.50 346.51 346.70 346.71 346.73 346.80 346.81 346.82 346.83 346.90 346.91 346.92 346.93 |
| Other headache | 339.09 339.11 339.12 339.20 339.21 339.22 339.3 339.41 339.42 339.44 339.82 339.83 339.84 339.85 784.0 |
| Prim thnderclap headache | 339.43 |
| Coma; stupor; and brain damage | 348.1 780.01 780.03 780.09 |
| Cataract associated with other disorders | 366.4 |
| Nonsenile cataract | 366.00 366.01 366.02 366.03 366.04 366.09 |
| Cataract | 366. 366.1 366.11 366.12 366.13 366.14 366.15 366.16 366.17 366.18 366.19 366.2 366.21 366.22 366.23 366.3 366.31 366.32 366.33 366.34 366.41 366.42 366.43 366.45 366.46 366.5 366.51 366.52 366.53 366.8 V43.1 |
| Other retinal disorders | 362. |
| Diabetic retinopathy | 362.0 362.01 362.02 362.04 362.05 362.06 |
| Retinal detachment with defect | 361.0 361.01 361.02 361.03 361.04 361.05 361.06 361.07 361.8 |
| Other retinal detachment or defect | 361.10 361.11 361.12 361.13 361.14 361.19 361.2 361.30 361.31 361.32 361.33 361.81 361.9 |
| Diabetic macular edema | 362.07 |
| Other retinal disorders | 362.10 362.11 362.12 362.13 362.14 362.15 362.16 362.17 362.18 |
| Retrolental fibroplasia | 362.21 |
| Retinopathy | 362.22 362.23 362.24 362.25 362.26 362.27 |
| Other nondiabetic proliferative retinopathy | 362.29 |
| Retinal vascular/venous occlusion | 362.3 362.30 362.31 362.32 362.35 362.36 362.37 |
| Retinal arterial occlusion | 362.33 362.34 |
| Other retinal disorders | 362.4 362.41 362.42 362.43 362.50 362.51 362.52 362.53 362.54 362.55 362.56 362.57 362.6 362.60 362.61 362.62 362.63 362.64 362.65 362.66 362.71 362.72 362.73 362.75 362.76 362.77 362.81 362.82 362.83 362.84 362.85 362.89 362.9 |
| Retinitis pigmentosa | 362.74 |
| Hereditary retinal dystrophies | 362.7 |
| Glaucoma-Open angle | 365.01 365.11 365.15 |
| Glaucoma-closed angle | 365.2 365.20 365.21 365.22 365.23 |
| Glaucoma | 365. 365.00 365.02 365.03 365.04 365.12 365.13 365.14 365.24 365.41 365.42 365.43 365.44 365.51 365.52 365.59 365.60 365.61 365.62 365.63 365.64 365.65 365.81 365.82 365.83 365.89 |
| Corticosteroid-induced glaucoma | 365.31 365.32 |
| Blindness and vision defects | 367. 367.0 367.1 367.2 367.21 367.22 367.31 367.32 367.4 367.5 367.51 367.52 367.53 367.8 367.81 367.9 368.00 368.01 368.02 368.03 368.1 368.10 368.11 368.12 368.13 368.14 368.15 368.16 368.2 368.30 368.31 368.32 368.33 368.34 368.4 368.41 368.42 368.43 368.44 368.45 368.46 368.47 368.5 368.51 368.52 368.53 368.54 368.55 368.59 368.6 368.61 368.62 368.63 368.69 368.8 368.9 369.00 369.01 369.02 369.03 369.04 369.05 369.06 369.07 369.08 369.10 369.11 369.12 369.13 369.14 369.15 369.16 369.17 369.18 369.20 369.21 369.22 369.23 369.24 369.25 369.3 369.4 369.61 369.62 369.63 369.64 369.65 369.66 369.67 369.68 369.69 369.70 369.71 369.72 369.73 369.74 369.75 369.76 369.8 369.9 V41.0 |
| Inflammation; infection of eye (except that caused by TB or STD) | 021.3 032.81 053.20 053.21 053.22 053.29 054.4 054.41 054.42 054.43 054.44 054.49 055.71 076. 076.0 076.1 077.0 077.1 077.2 077.3 077.4 077.8 077.98 077.99 115.02 115.12 115.92 130.1 130.2 139.1 360. 360.0 360.00 360.01 360.02 360.03 360.04 360.1 360.11 360.12 360.14 360.19 360.8 363.0 363.01 363.03 363.04 363.05 363.06 363.07 363.08 363.1 363.11 363.12 363.13 363.14 363.15 363.20 363.21 364.0 364.01 364.02 364.03 364.04 364.05 364.1 364.11 364.21 364.22 364.23 364.24 364.3 370.20 370.21 370.22 370.23 370.24 370.31 370.32 370.33 370.34 370.35 370.40 370.44 370.49 370.50 370.52 370.54 370.55 370.59 370.8 372.0 372.00 372.01 372.02 372.03 372.04 372.05 372.06 372.1 372.11 372.12 372.13 372.14 372.15 372.2 372.21 372.22 372.30 372.31 372.33 372.39 373.0 373.00 373.01 373.02 373.11 373.12 373.13 373.31 373.32 373.33 373.34 373.4 373.5 373.6 373.8 375. 375.0 375.01 375.02 375.03 375.30 375.31 375.32 375.33 375.41 375.42 375.43 376.0 376.01 376.02 376.03 376.04 376.1 376.11 376.12 376.13 377.3 377.32 377.33 377.34 377.39 379.00 379.01 379.02 379.03 379.04 379.05 379.06 379.07 379.09 379.60 379.61 379.62 379.63 |
| Other eye disorders | 360.2 360.21 360.23 360.24 360.29 360.3 360.31 360.32 360.33 360.34 360.41 360.42 360.43 360.44 360.5 360.51 360.52 360.53 360.54 360.55 360.59 360.60 360.61 360.62 360.63 360.64 360.65 360.69 360.81 363.3 363.31 363.32 363.33 363.34 363.35 363.4 363.41 363.43 363.5 363.51 363.52 363.53 363.54 363.55 363.56 363.57 363.61 363.62 363.63 363.7 363.71 363.72 363.8 363.9 364.41 364.42 364.51 364.52 364.53 364.54 364.55 364.57 364.59 364.60 364.61 364.62 364.63 364.70 364.71 364.72 364.73 364.74 364.75 364.76 364.77 364.81 364.89 364.9 370. 370.0 370.00 370.01 370.02 370.03 370.04 370.05 370.06 370.07 370.6 370.61 370.62 370.63 370.64 371.00 371.01 371.02 371.03 371.04 371.05 371.10 371.11 371.12 371.13 371.15 371.16 371.2 371.21 371.22 371.23 371.24 371.3 371.31 371.32 371.33 371.4 371.41 371.42 371.43 371.44 371.45 371.46 371.48 371.49 371.5 371.50 371.51 371.52 371.53 371.54 371.55 371.56 371.57 371.58 371.6 371.61 371.62 371.70 371.71 371.72 371.73 371.8 371.81 371.82 371.89 371.9 372.34 372.4 372.41 372.42 372.43 372.44 372.45 372.50 372.51 372.52 372.53 372.54 372.55 372.56 372.6 372.61 372.62 372.63 372.71 372.72 372.73 372.74 372.75 372.8 372.81 372.9 373.2 374. 374.00 374.01 374.02 374.03 374.04 374.05 374.1 374.11 374.12 374.13 374.14 374.2 374.21 374.22 374.23 374.3 374.31 374.32 374.33 374.34 374.41 374.43 374.44 374.45 374.46 374.50 374.51 374.52 374.53 374.54 374.55 374.56 374.81 374.82 374.83 374.84 374.85 374.86 374.87 374.9 375.11 375.12 375.13 375.14 375.15 375.16 375.2 375.20 375.21 375.22 375.51 375.52 375.53 375.54 375.55 375.56 375.57 375.6 375.61 375.8 375.81 375.9 376.21 376.22 376.30 376.31 376.32 376.33 376.34 376.35 376.36 376.4 376.41 376.42 376.44 376.45 376.46 376.47 376.5 376.51 376.52 376.6 376.81 376.82 376.89 376.9 377. 377.0 377.01 377.02 377.03 377.04 377.1 377.11 377.12 377.13 377.14 377.15 377.16 377.21 377.22 377.23 377.24 377.4 377.41 377.42 377.43 377.51 377.52 377.53 377.54 377.61 377.62 377.63 377.71 377.72 377.73 377.75 378.0 378.01 378.02 378.03 378.04 378.05 378.06 378.07 378.08 378.1 378.11 378.12 378.13 378.14 378.15 378.16 378.17 378.18 378.2 378.21 378.22 378.23 378.24 378.31 378.32 378.33 378.34 378.35 378.4 378.41 378.42 378.43 378.44 378.45 378.5 378.51 378.52 378.53 378.54 378.55 378.56 378.6 378.61 378.62 378.63 378.71 378.72 378.73 378.81 378.82 378.83 378.84 378.85 378.86 378.87 378.9 379.11 379.12 379.13 379.14 379.15 379.16 379.19 379.2 379.21 379.22 379.23 379.24 379.25 379.26 379.29 379.31 379.32 379.33 379.34 379.39 379.4 379.40 379.41 379.42 379.43 379.45 379.46 379.49 379.50 379.51 379.52 379.53 379.54 379.55 379.56 379.57 379.58 379.59 379.8 379.91 379.92 379.93 379.99 781.93 V41.1 V42.5 V43.0 V45.61 V45.69 V52.2 V53.1 V72.0 |
| Kayser-Fleischer ring | 371.14 |
| Otitis media and related conditions | 381.8 383.8 |
| Suppurative and unspecified otitis media | 382. 382.0 382.00 382.01 382.02 382.1 382.2 382.3 382.9 |
| Other otitis media and related conditions | 055.2 381.0 381.01 381.02 381.03 381.04 381.05 381.06 381.10 381.19 381.2 381.29 381.3 381.4 381.5 381.51 381.52 381.6 381.61 381.63 381.7 381.81 383.00 383.01 383.02 383.1 383.2 383.21 383.22 383.3 383.31 383.32 383.33 383.81 383.9 384.2 384.21 384.22 384.23 384.24 384.25 384.81 384.82 384.9 385. 385.0 385.01 385.02 385.03 385.09 385.1 385.11 385.12 385.13 385.19 385.21 385.22 385.23 385.24 387. 387.0 387.1 387.2 387.8 |
| Vertiginous syndromes and other disorders of vestibular system | 386. |
| Meniere's disease | 386.0 386.01 386.02 386.03 386.04 |
| Benign paroxysmal positional vertigo | 386.11 |
| Vertigo of central origin | 386.2 |
| Vertigo | 386.10 386.19 780.4 |
| Conditions associated with dizziness or vertigo | 386.12 386.3 386.31 386.32 386.33 386.34 386.4 386.41 386.42 386.43 386.48 386.5 386.51 386.52 386.53 386.54 386.55 386.56 386.58 386.8 386.9 |
| Other ear and sense organ disorders | 380. 380.00 380.01 380.02 380.03 380.1 380.10 380.11 380.12 380.13 380.14 380.15 380.16 380.21 380.22 380.23 380.30 380.31 380.32 380.39 380.4 380.5 380.51 380.52 380.53 380.8 380.81 384.00 384.01 384.09 384.1 385.3 385.30 385.31 385.32 385.35 385.82 385.83 385.9 388. 388.0 388.01 388.02 388.1 388.11 388.12 388.2 388.3 388.30 388.31 388.32 388.40 388.41 388.42 388.43 388.44 388.45 388.5 388.6 388.61 388.69 388.7 388.71 388.72 388.8 388.9 V41.3 V49.85 V53.2 V72.11 V72.12 V72.19 |
| Other ear and sense organ disorders | 389. 389.0 389.01 389.02 389.03 389.04 389.05 389.06 389.08 389.1 389.11 389.12 389.13 389.14 389.15 389.16 389.17 389.18 389.2 389.21 389.22 389.7 389.8 389.9 V41.2 |
| Critical illness polyneuropathy | 357.82 |
| Myopathy | 359.79 359.81 |
| Chr inflam polyneuritis | 357.81 |
| Inclusion body myositis | 359.71 |
| Mitochondrial myopathy | 359.89 |
| Trigeminal neuralgia | 350. 350.1 |
| Carpal/Radial tunnel syndrome | 354.0 354.3 |
| Hereditary peripheral neuropathy | 356.0 |
| Hereditary sensory neuropathy | 356.2 |
| Disorders of the peripheral nervous system | 350.2 350.8 351. 351.0 351.1 351.8 352.0 352.1 352.2 352.3 352.4 352.5 352.6 352.9 353. 353.0 353.1 353.2 353.3 353.4 353.5 353.6 353.8 354.1 354.2 354.4 354.5 354.8 354.9 355. 355.0 355.1 355.2 355.3 355.4 355.5 355.6 355.7 355.71 355.79 355.9 356.1 356.3 357. 357.0 357.1 357.2 357.3 357.4 357.6 357.7 357.8 357.9 358. |
| Myoneural disorders | 358.1 358.2 358.8 359.1 359.21 359.22 359.23 359.29 359.4 359.5 359.6 |
| Myopathy NOS | 359.8 359.9 |
| Periodic paralysis | 359.3 |
| Congenital hereditary muscular dystrophy | 359.0 |
| Lambert-Eaton syndrome | 358.3 |
| Myasthenia gravis | 358.0 358.00 358.01 |
| Idiopathic progressive polyneuropathy | 356.4 356.9 |
| Other specified idiopathic peripheral neuropathy | 356.8 |
| Thrombosis of superior longitudinal sinus | 325. |
| Circadian rhym sleep | 327.30 327.31 327.32 327.33 327.34 327.35 327.36 327.39 |
| Narcolepsy | 347. 347.0 347.00 347.10 347.11 |
| Other central nervous system disorders | 327.02 327.15 327.53 331.83 332.1 337.2 337.22 337.29 338.0 341. 341.0 341.1 341.8 341.9 344.61 348.0 348.2 348.30 348.31 348.39 348.4 348.5 348.81 348.89 348.9 349.2 349.8 349.81 349.82 349.89 349.9 V12.41 V53.01 |
| Other nervous system symptoms and disorders | 338.11 338.12 338.18 338.19 338.21 338.22 338.28 338.29 338.3 338.4 781.0 781.1 781.2 781.3 781.7 781.8 782. 782.0 784.3 784.51 784.59 784.6 784.60 784.61 784.69 792. 792.0 793.0 794.0 794.01 794.02 794.09 794.10 794.11 794.12 794.13 794.14 794.15 794.16 794.17 794.19 796.1 799.51 V12.4 V12.42 V12.49 V41.5 V45.2 V48.4 V48.5 V49.3 V53. V53.02 V53.09 |
| Other and ill-defined heart disease | 429.8 |
| Other and ill-defined heart disease | 429.2 429.9 459.89 459.9 785.9 794.3 794.39 |
| Hypertensive heart and/or renal disease | 402.10 403.00 403.01 403.1 403.11 403.9 403.90 403.91 404.00 404.01 404.02 404.03 404.10 404.11 404.12 404.13 404.90 404.91 404.92 404.93 405.01 405.11 405.91 |
| Hypertension | 401. 401.0 401.1 401.9 402.00 402.01 402.11 402.90 402.91 405.09 405.19 405.99 437.2 |
| Chronic rheumatic disease of the heart valves | 391.1 394.0 394.1 394.2 394.9 395.0 395.1 395.2 395.9 396. 396.0 396.1 396.2 396.3 396.8 397.0 397.1 397.9 |
| Myxoid transformation of mitral valve | 424.0 |
| Aortic valve stenosis | 424.1 |
| Tricuspid valve disorders, specified as nonrheumatic | 424.2 |
| Pulmonic incompetence NOS | 424.3 |
| Endocarditis | 424.9 424.90 424.91 424.99 |
| Abnormal heart sounds | 785.2 785.3 |
| Heart valve replaced by transplant | V42.2 V43.3 |
| Cardiac arrest and ventricular fibrillation | 427.41 427.42 427.5 V12.53 |
| Systolic hrt failure | 428.20 428.21 428.22 428.23 428.40 428.41 428.42 428.43 794.30 |
| Diastolc hrt failure | 428.30 428.31 428.32 428.33 |
| Heart failure | 428. 428.1 |
| Rheumatic heart failure (congestive) | 398.91 |
| Right heart failure | 428.0 |
| High output heart failure | 428.9 |
| Cardiomyopathy | 425. |
| Endomyocardial fibrosis | 425.0 |
| Hypertrophic cardiomyopathy | 425.1 425.11 425.18 |
| Obscure cardiomyopathy of Africa | 425.2 |
| Endocardial fibroelastosis | 425.3 |
| Primary idiopathic dilated cardiomyopathy | 425.4 |
| Nutritional and metabolic cardiomyopathy | 425.7 |
| Cardiomyopathy | 425.8 425.9 |
| Meningococcal endocarditis | 036.42 |
| Other peri-; endo-; and myocarditis-Coxsackie | 074.2 074.21 074.23 112.81 |
| Other peri-; endo-; and myocarditis-Histoplasma | 115.03 115.04 115.14 115.93 |
| Myocarditis due to toxoplasmosis | 130.3 |
| Septic myocarditis | 422.92 |
| Rheumatic fever with heart involvement | 391. 391.0 391.2 391.8 391.9 393. 398.0 |
| Rheumatic chorea with heart involvement | 392. 392.0 |
| Other rheumatic heart disease | 398. |
| Pericarditis | 420. 420.0 420.90 420.99 |
| Acute idiopathic pericarditis | 420.91 |
| Mycotic aneurysm | 421.0 |
| Acute endocarditis, | 421.1 421.9 |
| Myocarditis | 422. 422.0 422.9 429.0 |
| Idiopathic myocarditis | 422.91 |
| Hemopericardium | 423.0 |
| Adhesive pericarditis | 423.1 |
| Constrictive pericarditis | 423.2 |
| Cardiac tamponade | 423.3 |
| Other diseases of pericardium | 423.8 423.9 |
| Acute myocardial infarction | 410. 410.00 410.01 410.02 410.10 410.11 410.12 410.20 410.21 410.22 410.30 410.31 410.32 410.40 410.41 410.42 410.50 410.51 410.52 410.60 410.61 410.62 410.70 410.71 410.72 410.80 410.81 410.82 410.9 410.90 410.91 410.92 412. 429.79 |
| Cor ath natv art tp hrt | 414.06 |
| Angina pectoris | 413. 413.0 413.9 786.50 786.51 786.59 |
| Prinzmetal angina | 413.1 |
| Intermediate coronary syndrome | 411.1 |
| Other acute and subacute forms of ischemic heart disease | 411. 411.0 411.81 411.89 |
| Coronary atherosclerosis | 414.0 414.00 414.01 414.2 414.3 V45.81 V45.82 |
| Other forms of chronic heart disease | 414.8 414.9 |
| Pulmonary heart disease | 415.1 415.19 416.2 |
| Primary pulmonary hypertension | 416.0 |
| Septic pulmonary embolsm | 415.12 |
| Kyphoscoliotic heart disease | 416.1 |
| Cor pulmonale | 415.0 416. 416.8 416.9 417.9 |
| Arteriovenous fistula of pulmonary vessels | 417.0 |
| Other specified diseases of pulmonary circulation | 417.8 |
| Aneurysm of heart/arteries | 414.11 414.19 417.1 |
| Ventricular arteriovenous aneurysm | 414.10 |
| Rupture of chordae/papillary muscle | 429.5 429.6 |
| Other and ill-defined heart disease | 414.12 429.1 429.3 429.71 429.81 429.82 |
| Takotsubo syndrome | 429.83 |
| Restrictive heart disease | 429.89 |
| Conduction disorders | 426. 426.8 |
| Atrioventricular block | 426.0 426.10 426.11 426.12 426.13 |
| LBBB | 426.2 426.3 |
| RBBB | 426.4 426.50 426.51 426.52 |
| Bundle branch block | 426.53 426.54 |
| Wolff-Parkinson-White syndrome | 426.7 |
| Sinus arrest | 426.6 |
| Lown-Ganong-Levine syndrome | 426.81 |
| Long QT syndrome | 426.82 |
| Dissociation, atrioventricular | 426.89 |
| Stokes-Adams syndrome | 426.9 |
| Cardiac pacemaker in situ | V45.0 V53.3 |
| Automatic implantable cardiac defibrillator in situ | V45.02 V53.32 |
| Unspecified cardiac device in situ | V45.00 V45.09 V53.39 |
| Other specified cardiac dysrhythmias | 427.8 |
| Cardiac dysrhythmias | 427. |
| PAT | 427.0 |
| AVNRT | avnrt.1 |
| Paroxysmal tachycardia, unspecified | 427.2 |
| Ventricular tachycardia | 427.1 |
| Paroxysmal atrial fibrillation | 427.31 |
| Atrial flutter | 427.32 |
| Premature beats | 427.6 427.60 427.61 427.69 |
| Syndrome, tachycardia-bradycardia | 427.81 |
| Withdrawal arrhythmia | 427.89 |
| Ventricular arrhythmia | 427.9 |
| Tachycardia, unspecified | 785.0 785.1 |
| Other late effects of cerebrovascular disease | 438.8 |
| Intracranial hemorrhage | 430. 431. 432.0 432.1 432.9 |
| Occlusion of cerebral arteries | 434.0 434.00 434.01 |
| Occlusion of cerebral arteries | 434.1 434.10 434.11 |
| Occlusion of cerebral arteries | 434.90 434.91 |
| Acute but ill-defined cerebrovascular accident | 346.60 346.61 436. |
| Occlusion of cerebral arteries | 433.00 433.01 433.10 433.11 433.20 433.21 433.30 433.31 433.80 433.81 433.90 433.91 434. |
| Other and ill-defined cerebrovascular disease | 437. 437.0 437.1 437.4 437.5 437.7 437.9 |
| Nonpyogenic thrombosis of intracranial venous sinus | 437.6 |
| Intracranial aneurysm | 437.3 |
| Transient cerebral ischemia | 435. 435.0 435.1 435.2 435.3 435.8 435.9 V12.54 |
| Late effects of cerebrovascular disease | 438. 438.0 438.10 438.11 438.12 438.14 438.19 438.20 438.21 438.22 438.30 438.31 438.32 438.40 438.41 438.42 438.50 438.51 438.52 438.53 438.6 438.81 438.82 438.83 438.84 438.85 |
| Atherosclerosis of arteries of extremities | 440.20 440.21 440.22 440.23 440.29 440.4 |
| Spasm of artery | 443.9 |
| Other peripheral and visceral atherosclerosis | 440. 440.0 440.8 440.9 557. |
| Other peripheral and visceral atherosclerosis | 557.1 557.9 |
| Thrombosis of mesenteric vein | 557.0 |
| Renal artery stenosis | 440.1 |
| Arterial dissection | 443.21 443.22 443.23 443.24 443.29 |
| Abdominal aortic aneurysm without mention of rupture | 441.4 |
| Aortic aneurysm | 441.0 441.00 441.01 441.02 441.03 441.1 441.2 441.3 441.5 441.6 441.7 441.9 447.71 |
| Other aneurysm | 442.0 442.1 442.2 442.3 442.81 442.82 442.83 442.84 442.89 |
| Pseudoaneurysm | 442.9 |
| Aortic and peripheral arterial embolism or thrombosis | 444.01 444.09 444.1 444.21 444.22 444.81 444.89 444.9 445.01 445.02 445.81 445.89 |
| Hypotension | 458. |
| Other specified hypotension | 458.8 |
| Pure autonomic failure | 458.0 |
| Chronic hypotension | 458.1 |
| Other peripheral vascular disease | 443. |
| Raynaud's syndrome | 443.0 |
| Thromboangiit obliterans | 443.1 |
| Peripheral angiopathy in diseases classified elsewhere | 443.81 |
| Erythromelalgia | 443.82 |
| Acrocyanosis | 443.89 |
| Polyarteritis nodosa | 446.0 |
| Mucocutan lymph node syn | 446.1 |
| Hypersensitivity angiitis | 446.2 |
| Mixed collagen vascular disease | 446.20 |
| Goodpasture's syndrome | 446.21 |
| Other specified hypersensitivity angiitis | 446.29 |
| Lethal midline granuloma | 446.3 |
| Wegener's granulomatosis | 446.4 |
| Temporal arteritis | 446.5 |
| Thrombotic microangiopathy | 446.6 |
| Takayasu's disease | 446.7 |
| Arteriovenous fistula, acquired | 447.0 |
| Stricture of artery | 447.1 |
| Rupture of artery | 447.2 |
| Hyperplasia of renal artery | 447.3 |
| Celiac artery compression syndrome | 447.4 |
| Other and unspecified circulatory disease | 447.5 447.6 |
| Other specified disorders of arteries and arterioles | 447.8 |
| Other and unspecified circulatory disease | 447.9 448. |
| Hereditary hemorrhagic telangiectasia | 448.0 |
| Nevus, non-neoplastic | 448.1 |
| Other and unspecified capillary diseases | 448.9 |
| Hemorrhage NOS | 459.0 |
| Wide QRS complex/Abnormal EKG | 794.31 |
| Other and unspecified circulatory disease | 796.2 V12.50 V12.59 V15.1 |
| Heart replaced by transplant | V42.1 V43.22 |
| Heart assist dev replace | V43.21 |
| Blood vessel replaced by other means | V43.4 |
| Observation for suspected cardiovascular disease | V71.7 |
| Phlebitis and thrombophlebitis | 451. 451.0 451.11 451.19 451.2 451.8 451.81 451.82 451.83 451.84 451.89 451.9 V12.52 |
| Portal vein thrombosis | 452. |
| Budd-Chiari syndrome | 453.0 |
| Thrombophlebitis migrans | 453.1 |
| Other venous embolism and thrombosis | 453.2 453.3 453.40 453.41 453.42 453.50 453.51 453.52 453.71 453.72 453.73 453.74 453.75 453.76 453.77 453.79 453.82 453.83 453.84 453.85 453.86 453.87 453.89 453.9 V12.51 |
| Embl suprfcl ves | 453.6 453.81 |
| Varicose veins of lower extremity | 454.0 454.1 454.2 454.8 454.9 |
| Hemorrhoids | 455. 455.0 455.1 455.2 455.3 455.4 455.5 455.6 455.7 455.8 455.9 |
| Varices | 456.3 456.4 456.5 456.6 456.8 |
| Noninfectious disorders of lymphatic channels | 457. |
| Other diseases of veins and lymphatics | 457.0 457.1 457.2 |
| Other noninfectious disorders of lymphatic channels | 457.8 |
| Postphlebitic syndrome | 459.1 459.10 459.11 459.12 459.13 459.19 |
| Superior vena cava syndrome | 459.2 |
| Other diseases of veins and lymphatics | 459.30 459.31 459.32 459.33 459.39 459.81 |
| Pneumococcal pneumonia | 481. |
| Other bacterial pneumonia | 003.22 020.4 020.5 021.2 022.1 031.0 039.1 482.0 482.1 482.2 482.31 482.32 482.39 482.4 482.41 482.42 482.49 482.8 482.81 482.82 482.83 482.84 482.9 486. |
| Other pneumonia | 052.1 073. 073.0 083.0 112.4 114. 114.0 114.4 114.5 115.05 115.15 115.95 130.4 136.3 480. 480.0 480.1 480.2 480.3 480.8 483. 483.0 483.1 484.1 484.3 484.6 484.7 484.8 485. 513.0 517.1 |
| Influenza | 487. 487.0 487.1 487.8 |
| Acute and chronic tonsillitis | 463. 474.0 474.00 474.01 474.1 474.11 474.12 474.2 474.8 474.9 475. |
| Acute bronchitis | 466.0 466.1 466.11 466.19 |
| Acute upper respiratory infections of multiple or unspecified sites | 465.8 465.9 |
| Chronic sinusitis | 473. 473.0 473.1 473.2 473.3 473.8 473.9 784.91 |
| Croup | 464.4 |
| Other and unspecified upper respiratory infections | 032. 032.0 032.2 032.3 034.0 460. 461. 461.0 461.1 461.2 461.3 461.8 462. 464.0 464.00 464.01 464.1 464.10 464.11 464.20 464.21 464.3 464.30 464.31 464.50 464.51 465.0 |
| Emphysema | 492. 492.0 492.8 |
| Chronic obstructive lung disease | 496. |
| Obstructive chronic bronchitis | 491.20 491.21 491.22 |
| Tracheobronchitis NOS | 490. |
| Chronic bronchitis | 491. 491.0 491.1 491.8 |
| Bronchiolectasis | 494. 494.0 494.1 |
| Chronic obstructive asthma without status asthmaticus or exacerbation | 493.20 493.21 493.22 |
| Other asthma without status asthmaticus or exacerbation | 493. 493.0 493.00 493.10 493.82 493.9 493.90 |
| Other asthma with status asthmaticus | 493.01 493.02 493.11 493.12 493.81 493.91 493.92 |
| Pneumonitis due to inhalation of food or vomitus | 507.0 |
| Pleurisy; pleural effusion | 511. 511.0 511.1 |
| Unspecified pleural effusion | 511.9 |
| Hemothorax | 511.89 |
| Pulmonary collapse; interstitial and compensatory emphysema | 518.0 518.1 518.2 |
| Empyema | 510. |
| Fistula, bronchopleural | 510.0 |
| Pyothorax | 510.9 |
| Spontaneous pneumothorax NOS | 512.0 512.89 |
| Depend-supplement oxygen | V46.2 |
| Respiratory failure | 518.81 518.84 |
| Other respiratory insufficiency | 517.3 518.52 518.82 518.83 799.1 V46.11 V46.12 V46.13 V46.14 |
| Lung disease due to external agents | 495. 495.0 495.1 495.2 495.3 495.4 495.5 495.6 495.7 495.8 500. 501. 502. 503. 504. 505. 506.0 506.1 506.2 506.3 506.4 506.9 507.1 507.8 508.0 508.1 508.8 508.9 |
| Postinflammatory pulmonary fibrosis | 515. |
| Pleurodynia | 786.52 |
| Abscess of mediastinum | 513.1 |
| Pulmonary edema NOS | 514. |
| Pulmonary alveolar proteinosis | 516.0 |
| Idiopathic pulmonary hemosiderosis | 516.1 |
| Pulmonary alveolar microlithiasis | 516.2 |
| Other and unspecified lower respiratory disease | 516.30 516.31 |
| Lung involvement in systemic sclerosis | 517.2 |
| Lung involvement in other diseases classified elsewhere | 517.8 |
| Other and unspecified lower respiratory disease | 786.3 786.30 |
| Other and unspecified lower respiratory disease | 516.37 516.4 516.8 516.9 518.3 518.4 518.89 519.4 519.8 519.9 782.5 786.00 786.01 786.02 786.03 786.04 786.05 786.06 786.07 786.09 786.2 786.4 786.6 786.7 786.8 786.9 793.11 793.19 V12.61 |
| Hx resp system dis NEC | V12.69 |
| Nonspecific abnormal results of function study of pulmonary system | 794.2 |
| Lung replaced by transplant | V42.6 |
| reactive airway disease | reacair.1 |
| Other polyp of sinus | 471.1 471.8 471.9 472.0 478.4 |
| Epistaxis | 784.7 |
| Stenosis of trachea | 519.19 |
| Laryngeal papillomatosis | larpap.1 |
| Other upper respiratory disease, infection | 478.21 478.22 478.24 |
| Other upper respiratory disease | 470. 472.1 472.2 476.0 476.1 477. 477.0 477.2 477.8 477.9 478. 478.0 478.1 478.11 478.19 478.20 478.25 478.26 478.29 478.30 478.31 478.32 478.33 478.34 478.5 478.6 478.7 478.70 478.71 478.74 478.75 478.79 478.8 519.11 519.2 519.3 784.1 784.40 784.41 784.42 784.43 784.44 784.49 784.8 784.9 784.99 786.1 V41.4 V44. V44.0 V55. V55.0 |
| Intestinal infection | 008.4 |
| Intestinal infection | 001. 001.1 002.0 002.1 002.2 002.3 002.9 003.0 003.2 003.29 003.8 003.9 004. 004.0 004.1 004.3 004.8 005. 005.0 005.1 005.2 005.81 005.9 007.0 007.1 007.3 007.4 007.8 007.9 008.00 008.01 008.02 008.04 008.09 008.2 008.3 008.41 008.42 008.43 008.44 008.45 008.46 008.47 008.5 008.6 008.61 008.62 008.63 008.64 008.67 008.69 008.8 009.0 009.1 009.2 009.3 021.1 022.2 07.2 |
| Intestinal infection-Amebic | 006. 006.0 006.1 006.2 006.3 006.4 006.5 006.6 006.8 006.9 |
| Gastric ulcer | 531. 531.00 531.01 531.20 531.21 531.40 531.41 531.60 |
| Duodenal ulcer | 532. 532.00 532.01 532.20 532.40 532.41 532.60 |
| Peptic ulcer, site unspecified | 533. 533.0 533.01 533.20 533.21 533.40 533.41 533.60 533.61 |
| Gastrojejunal ulcer | 534. 534.00 534.01 534.20 534.40 534.41 534.60 |
| Stool flecked with blood | 578.1 |
| Gastroesophageal laceration-hemorrhage syndrome | 530.7 |
| Esophageal varices | 456. 456.0 456.1 456.20 456.21 |
| Esophageal hemorrhage | 530.82 |
| Rectal hemorrhage | 569.3 |
| Gastrointestinal hemorrhage | 578. |
| Hematemesis | 578.0 |
| Jejunal hemorrhage | 578.9 |
| Other and unspecified noninfectious gastroenteritis and colitis | 558. |
| Eosinophilic colitis | 558.41 558.42 |
| Inflammatory bowel disease | 558.9 |
| Dieulafoy les, intestine | 569.86 |
| Constipation | 564.0 564.01 564.02 564.09 |
| Dysphagia | 787.2 787.20 787.21 787.22 787.23 787.24 787.29 |
| Irritable bowel syndrome | 564.1 |
| Retroperitoneal hemorrhage | 568.81 |
| GI Fistula/ulceration | 569.81 569.82 |
| Angiodysplasia of intestine | 569.84 569.85 |
| Tropical sprue | 579.1 |
| Celiac infantilism | 579.0 |
| Toxic megacolon | 564.7 |
| Steatorrhea | 579.8 |
| Diseases of the digestive system | 538. 558.1 558.2 564.5 564.81 564.89 564.9 568.0 568.8 568.82 568.89 568.9 569.8 569.83 569.87 569.89 569.9 579.2 579.9 787.1 787.3 787.4 787.5 787.6 787.7 787.9 787.91 787.99 V41.6 |
| Abdominal bruit | 789.9 |
| Splenomegaly | 789.2 |
| Abdominal or pelvic swelling, mass, or lump | 789.30 789.31 789.32 789.33 789.34 789.35 789.36 789.37 789.39 |
| Abdominal rigidity | 789.4 789.41 789.42 789.43 789.44 789.45 789.46 789.47 789.49 |
| Nonspecific abnormal findings in stool contents | 792.1 |
| Nonspecific abnormal findings on radiological and other examination of | 793.4 793.6 |
| Personal history of unspecified digestive disease | V12.70 V12.79 |
| GI surgery | V44.1 V44.2 V44.3 V44.4 V45.3 V47.3 V53.50 V53.51 V53.59 V55.1 V55.2 V55.3 V55.4 |
| Supernumerary teeth | 520.1 |
| Hereditary disturbances in tooth structure, not elsewhere classified | 520.5 |
| Other specified disorders of tooth development and eruption | 520.8 |
| Diseases of the digestive system | 520. 520.0 520.2 520.3 520.4 520.6 520.7 520.9 521. 521.0 521.01 521.02 521.03 521.04 521.05 521.06 521.08 521.09 521.10 521.12 521.20 521.21 521.22 521.24 521.25 521.30 521.31 521.32 521.33 521.34 521.35 521.40 521.41 521.5 521.6 521.7 521.8 521.81 522.0 522.1 522.2 522.3 522.4 522.5 522.6 522.7 522.8 522.9 523.00 523.01 523.10 523.11 523.2 523.20 523.22 523.23 523.24 523.25 523.30 523.31 523.32 523.33 523.4 523.40 523.41 523.42 523.5 523.6 523.8 523.9 524.0 524.01 524.02 524.03 524.04 524.05 524.06 524.07 524.09 524.1 524.11 524.12 524.19 524.2 524.21 524.22 524.23 524.24 524.25 524.26 524.27 524.28 524.29 524.30 524.31 524.32 524.33 524.34 524.35 524.36 524.37 524.39 524.4 524.5 524.51 524.52 524.53 524.55 524.57 524.59 524.61 524.62 524.63 524.64 524.69 524.7 524.71 524.73 524.74 524.75 524.76 524.79 524.8 524.81 524.82 524.9 525.0 525.10 525.11 525.12 525.13 525.19 525.20 525.21 525.22 525.23 525.24 525.25 525.26 525.3 525.40 525.41 525.50 525.51 525.52 525.60 525.62 525.64 525.67 525.71 525.73 525.79 525.8 525.9 526. 526.0 526.1 526.2 526.4 526.5 526.61 526.69 526.8 526.81 784.92 V52.3 V53.4 V58.5 V72.2 |
| Central giant cell (reparative) granuloma | 526.3 |
| Temporomandibular joint disorders | 524.6 524.60 |
| Sialolithiasis | 527.5 |
| Xerostomia | 527.7 |
| Mikulicz's disease | 527.1 |
| Diseases of mouth; excluding dental | 527. 527.0 527.2 527.3 527.4 527.6 527.8 527.9 528.00 528.09 528.1 528.2 528.3 528.4 528.5 528.6 528.7 528.71 528.72 528.79 528.8 528.9 529.0 529.1 529.2 529.3 529.5 529.6 529.8 529.9 792.4 |
| Atrophy of tongue papillae | 529.4 |
| Other specified disorders of stomach and duodenum | 537.8 |
| Esophagitis | 530.1 530.11 530.12 530.13 530.19 |
| Ulcer of esophagus | 530.2 530.20 530.21 |
| Other esophageal disorders | 530. 530.3 530.4 530.6 530.8 530.81 530.84 530.89 |
| Esophagospasm | 530.5 |
| Esophageal leukoplakia | 530.83 |
| Achalasia and cardiospasm | 530.0 |
| Nutcracker esophagus | 530.9 |
| Barrett's esophagus | 530.85 |
| Gastric ulcer | 531.10 531.11 531.30 531.31 531.50 531.51 531.70 531.71 531.9 531.90 531.91 |
| Duodenal ulcer | 532.10 532.11 532.30 532.31 532.50 532.51 532.70 532.71 532.90 532.91 |
| Peptic ulcer; site unspecified | 533.10 533.11 533.30 533.50 533.51 533.70 533.71 533.90 533.91 V12.71 |
| Gastrojejunal ulcer | 534.10 534.11 534.30 534.31 534.50 534.70 534.71 534.90 534.91 |
| Acute gastritis | 535.0 535.00 535.01 |
| Atrophic gastritis | 535.10 535.11 |
| Chronic gastritis | 535.1 535.40 |
| Gastric mucosal hypertrophy | 535.2 535.20 535.21 |
| Bile induced gastritis | 535.4 535.41 |
| Eosinophil gastrt | 535.70 535.71 |
| Unspecified gastritis and gastroduodenitis | 535.50 535.51 |
| Duodenitis | 535.60 535.61 |
| Pyloric stenosis | 537.0 |
| Other disorders of stomach and duodenum | 537.3 537.4 |
| Achlorhydria | 536.0 |
| Angiodysplasia of stomach and duodenum | 537.82 537.83 |
| Other disorders of stomach and duodenum | 536. 536.1 536.2 536.3 536.8 536.9 537.1 537.2 537.5 537.6 537.81 537.89 537.9 |
| Dieulafoy les,stom&duod | 537.84 |
| Inguinal hernia with obstruction or gangrene | 550. 550.00 550.01 550.02 550.03 550.10 550.11 550.12 550.13 |
| Inguinal hernia without obstruction or gangrene | 550.90 550.91 550.92 550.93 |
| Diaphragmatic hernia | 551.3 552.3 553.3 |
| Femoral hernia with obstruction/gangrene | 551.00 551.01 551.03 552.00 552.01 552.02 553.00 553.01 553.02 553.03 |
| Umbilical hernia with obstruction/gangrene | 551.1 551.2 551.21 551.29 551.8 551.9 552.1 552.2 552.21 552.29 552.8 552.9 553.1 553.20 553.21 553.29 553.8 hernia.1 |
| Other specified disorders of rectum and anus | 569.4 |
| Acute appendicitis with abscess or peritonitis | 540. 540.0 540.1 540.9 541. 542. |
| Other diseases of appendix | 543. |
| Hyperplasia of appendix (lymphoid) | 543.0 |
| Eneritis/ulcerative enteritis | 555. 555.1 555.2 556.9 |
| Regional Ileitis | 555.0 |
| Crohn's disease/Ulcerative colitis | 555.9 556. 556.0 556.1 556.2 556.3 556.5 556.6 556.8 |
| Pseudopolyposis of colon | 556.4 |
| Other specified intestinal obstruction | 560.8 |
| Paralytic ileus | 560.1 |
| Impaction of intestine | 560.3 560.32 560.39 |
| Intussusception | 560.0 |
| Volvulus of the small bowel | 560.2 |
| Gallstone ileus | 560.31 |
| Other intestinal obstruction | 560.81 560.9 |
| Diverticulosis | 562. 562.00 562.02 562.10 562.12 |
| Diverticulitis | 562.01 562.03 562.11 562.13 |
| Anal and rectal conditions | 565.0 565.1 569.2 |
| Anal and rectal conditions | 564.6 566. 569.0 569.1 569.41 569.42 569.43 569.44 |
| Proctitis NOS | 569.49 |
| Peritonitis and intestinal abscess | 032.83 567.0 567.1 567.2 567.21 567.22 567.23 567.38 567.39 567.8 567.81 569.5 |
| Peritonitis and intestinal abscess | 567.82 567.89 |
| Unspecified peritonitis | 567.9 |
| Cholelithiasis with acute cholecystitis | 574. 574.00 574.01 |
| Cholelithiasis with other cholecystitis | 574.10 574.11 |
| Cholelithiasis without mention of cholecystitis | 574.20 574.21 |
| Calculus of bile duct | 574.30 574.31 574.40 574.41 574.50 574.51 574.60 574.61 574.70 574.71 574.80 574.81 574.90 574.91 |
| Cholecystitis without cholelithiasis | 575.0 575.10 575.11 575.12 |
| Suppurative cholangitis | 576.1 |
| Other biliary tract disease | 575.2 575.3 575.4 575.5 575.6 575.8 575.9 576.0 576.2 576.3 576.4 576.5 576.9 793.3 |
| Other specified disorders of biliary tract | 576.8 |
| Macronodular cirrhosis | 571.5 |
| Pyogenic hepatic abscess/pyemia | 572.0 572.1 |
| Sequelae of chronic liver disease | 572.2 572.3 572.4 572.8 |
| Ascites | 789.5 789.59 |
| Unspecified chronic liver disease without mention of alcohol | 571.9 |
| Secondary biliary cirrhosis | 571.6 |
| Steatohepatitis | 571.8 |
| Other and unspecified liver disorders | 570. 573.0 782.4 |
| Unspecified disorder of liver | 573.9 |
| Hepatic infarction | 573.4 |
| Other nonspecific abnormal serum enzyme levels | 790.4 790.5 794.8 |
| Hepatomegaly | 789.1 |
| Other specified disorders of liver | 573.8 |
| Liver replaced by transplant | V42.7 |
| Phlegmon of pancreas | 577.0 |
| Chronic pancreatitis | 577.1 577.2 |
| Pancreatic insufficiency | 577.8 |
| Unspecified disease of pancreas | 577.9 |
| Other pancreatic disorders | 579.4 |
