# Supplementary material for: Integrating EMR-Linked and In Vivo Functional Genetic Data to Identify New Genotype-Phenotype Associations
Source: PLoS One. 2014 Jun 20;9(6):e100322. doi: 10.1371/journal.pone.0100322 (PMC4065041; doi:10.1371/journal.pone.0100322)
Supplement: Table S3 — Phenotype definitions. (DOCX) [file pone.0100322.s003.docx]

**Supplemental table 3. Phenotype definitions.**  Shown for each SNP are clinical phenotype definitions based on the phenotypes in the OMIM and KO mouse databases. An asterisk in the “Defined from cases” column indicates that the phenotype definition was defined, in part, based on the observed problems in the homozygous minor allele subjects. An asterisk (*) in the “Opposite phenotype” column indicates that the phenotype was defined to be the opposite of what has been previously reported, based on the clinical problems observed on preliminary review. The ICD-9 codes selected for each phenotype definition are listed. Any subject with any of the listed ICD-9 codes was considered to have the phenotype.

| **Gene /**  **SNP** | **Previously reported phenotypes** | **Phenotype source** | **Defined from minor allele homozygotes** | **Opposite phenotype** | **Specific phentoypes** | **ICD-9 codes** |
| --- | --- | --- | --- | --- | --- | --- |
| ADAM22  rs17255978 | Peripheral neuropathy | KO mouse |  |  | Peripheral neuropathy (excludes diabetes) | 337.1, 357.82, 356.0, 356.2, 355.0, 355.9, 357., 357.1, 357.3, 357.4, 356.4, 356.9, 356.8 |
|  |  |  |  |  | Demyelination disease | 341., 341.8, 341.9 |
|  |  |  |  |  | Seizures | 345.00, 345.0, 345.01, 345.1, 345.10, 345.11, 345.2, 345.3, 345.4, 345.40, 345.41, 345.5, 345.50, 345.51, 345.60, 345.61, 345.70, 345.71, 345.80, 345.81, 345.9, 345.90, 345.91 |
|  |  |  |  |  |  |  |
| ADAMTS13  rs28647808 | Familial TTP | OMIM, KO mouse |  |  | Thrombocytopenia | 283.71, 287.30, 287.32, 287.33, 287.39, 287.5, 287.1 |
|  |  |  |  |  |  |  |
| AOC3  rs33986943 | Abnormal leukocyte adhesion; decreased lymphocytes in Peyer patches; decreased reduced serum IgA | KO mouse |  |  | All sepsis | 038., 038.0, 038.1, 038.11, 038.12, 038.19, 038.4, 038.40, 038.41, 038.43, 038.44, 003.1, 020.2, 022.3, 036.2, 038.2, 038.3, 054.5, 038.42, 038.8, 038.9, 771.81, 790.7, 670.2, 670.22, 995.9, 995.91, 995.92, 771.81, 449. |
|  |  |  |  |  | Gram positive sepsis | 038., 038.0, 038.1, 038.11, 038.12, 038.19, 036.2, 038.2 |
|  |  |  |  |  | Gram negative sepsis | 038.4, 038.40, 038.41, 038.43, 038.44, 003.1, 020.2, 022.3, 054.5, 038.42, 038.3 |
|  |  |  |  |  | Decrease serum IgA | 279.0, 279.00, 279.01, 279.02, 279.03 |
|  |  |  |  |  |  |  |
| CACNA1A  rs16027 | Migraine, familial hemiplegic | OMIM, KO mouse |  |  | Migraine | 346., 346.00, 346.01, 346.02, 346.03, 346.1, 346.11, 346.12, 346.13, 346.2, 346.20, 346.21, 346.22, 346.30, 346.50, 346.51, 346.70, 346.71, 346.73, 346.80, 346.81, 346.82, 346.83, 346.90, 346.91, 346.92, 346.93 |
|  |  |  |  |  | Seizures | 345.00, 345.0, 345.01, 345.1, 345.10, 345.11, 345.2, 345.3, 345.4, 345.40, 345.41, 345.5, 345.50, 345.51, 345.60, 345.61, 345.70, 345.71, 345.80, 345.81, 345.9, 345.90, 345.91 |
|  |  |  |  |  | Convulsions | 780.3, 780.31, 780.32, 780.39 |
|  |  |  |  |  |  |  |
| CACNA2D4  rs2286372 | Retinal cone dystrophy | OMIM |  |  | Retinal disease | all 362, 368.34, 363.3, 363.32, 363.33, 363.34, 363.35, 377.03, 377.13, 794.11 |
|  |  |  |  |  | Retinal disease (exlude HTN and DM) | 362.0, 362.01, 362.02, 362.04, 362.05, 362.06, 362.07, 362.10, 362.11 |
|  |  |  |  |  |  |  |
| CLEC1B  rs2273987 | Abnormal blood vessel morphology, intracranial hemorrhage | KO Mouse |  |  | Intracranial hemorrhage | 430., 431., 432.0, 432.1, 432.9 |
|  |  |  |  |  |  |  |
| CNGB3  rs3735972 | Macular degeneration, juvenile / Achromatopsia | OMIM, KO mouse |  |  | Cataract | 366.4, 366.00, 366.01, 366.02, 366.03, 366.04, 366.09, 998.82, 366., 366.1, 366.11, 366.12, 366.13, 366.14, 366.15, 366.16, 366.17, 366.18, 366.19, 366.2, 366.21, 366.22, 366.23, 366.3, 366.31, 366.32, 366.33, 366.34, 366.41, 366.42, 366.43, 366.45, 366.46, 366.5, 366.51, 366.52, 366.53, 366.8, V43.1, V45.61 |
|  |  |  |  |  | Macular degeneration | 362.50, 362.51, 362.52, 362.53, 362.05 |
|  |  |  |  |  | Colorblindness | 368.5, 368.55, 368.59 |
|  |  |  |  |  | Retinal disease (exlude HTN and DM) | 362.0, 362.01, 362.02, 362.04, 362.05, 362.06, 362.07, 362.10, 362.11 |
|  |  |  |  |  | Cataract (age>50) |  |
|  |  |  |  |  |  |  |
| DNAH5  rs2277046 | Ciliary dyskinesia | OMIM |  |  | Situs inversus |  |
|  |  |  |  |  | URI | 465.9,465.8 |
|  |  |  |  |  | Sinusitis | 473., 473.0, 473.1, 473.2, 473.3, 473.8, 473.9, 461., 461.0, 461.1, 461.2, 461.3, 461.8 |
|  |  |  |  |  | Chronic sinusitis | 473., 473.0, 473.1, 473.2, 473.3, 473.8, 473.9 |
|  |  |  |  |  | Pneumonia (bacterial) | 481.1,003.22, 020.4, 020.5, 021.2, 022.1, 031.0, 039.1, 482.0, 482.1, 482.2, 482.31, 482.32, 482.39, 482.4, 482.41, 482.42, 482.49, 482.8, 482.81, 482.82, 482.83, 482.84, 482.9, 486. |
|  |  |  |  |  | Bronchiectasis | 494.1 |
|  |  |  | * |  | Other pulmonary: Pleurodynia | 786.52 |
|  |  |  |  |  |  |  |
| ERCC4  rs1800067 | Xeroderma pigmentosusm | OMIM |  |  | Seborrheic keratosis | 702.1, 702.11, 702.19 |
|  |  |  |  |  |  |  |
| F5  rs6031 | Factor V deficiency | OMIM, KO Mouse |  |  | Venous thrombosis | 452., 453.0, 453.1, 453.2, 453.3, 453.40, 453.41, 453.42, 453.50, 453.51, 453.52, 453.71, 453.72, 453.73, 453.74, 453.75, 453.76, 453.77, 453.79, 453.82, 453.83, 453.84, 453.85, 453.86, 453.87, 453.89, 453.9, V12.51 |
|  |  |  |  |  | On anti-coagulant | V58.61 |
|  |  |  |  |  | Pregnancy loss | 634., 634.0, 634.01, 634.02, 634.10, 634.11, 634.12, 634.21, 634.22, 634.30, 634.31, 634.32, 634.4, 634.41, 634.42, 634.50, 634.51, 634.52, 634.60, 634.62, 634.7, 634.71, 634.72, 634.8, 634.81, 634.82, 634.9, 634.90, 634.91, 634.92, 632. |
|  |  |  |  |  | Budd-Chiari syndrome | 453.0 |
|  |  |  |  |  | Stroke | 434.0, 434.00, 434.01, 434.1, 434.10, 434.11, 434.90, 434.91, 346.60, 346.61, 436., 433.01, 433.11, 433.21, 433.31, 433.81, 433.91, 434., 433.00, 433.10, 433.20, 433.30, 433.80, 433.90 |
|  |  |  |  |  |  |  |
| FBN2  rs2291628 | Syn/polydactyly, osteoporosis, abnormal bone remodelling | OMIM, KO mouse |  |  | Osteoporosis | V17.81, 733.0, 733.00, 733.01, 733.02, 733.03, 733.09 |
|  |  |  |  |  | Polydactyly | 755.0, 755.01, 755.02, 755.10, 755.11, 755.12, 755.13, 755.14, 755.3, 755.31, 755.32, 755.33, 755.34, 755.35, 755.36, 755.37, 755.38, 755.39, 755.61, 755.62, 755.63 |
|  |  |  |  |  | Joint disease | 718.1, 718.11, 718.12, 718.13, 718.14, 718.15, 718.17, 718.18, 718.19, 718.21, 718.22, 718.24, 718.25, 718.26, 718.27, 718.28, 718.29, 718.5, 718.51, 718.52, 718.53, 718.54, 718.55, 718.56, 718.57, 718.58, 718.59, 718.65, 718.70, 718.71, 718.72, 718.73, 718.74, 718.75, 718.76, 718.77, 718.80, 718.81, 718.82, 718.83, 718.84, 718.85, 718.86, 718.87, 718.88, 718.89, 718.9, 718.91, 718.92, 718.93, 718.94, 718.95, 718.97, 718.98, 718.99, 719.0, 719.01, 719.02, 719.03, 719.04, 719.05, 719.06, 719.07, 719.08, 719.09, 719.4, 719.41, 719.42, 719.43, 719.44, 719.45, 719.46, 719.47, 719.48, 719.49, 719.50, 719.51, 719.52, 719.53, 719.54, 719.55, 719.56, 719.57, 719.58, 719.59, 719.60, 719.61, 719.62, 719.63, 719.64, 719.65, 719.66, 719.67, 719.68, 719.69, 719.7, 719.80, 719.81, 719.82, 719.83, 719.84, 719.85, 719.86, 719.87, 719.88, 719.89, 719.91, 719.92, 719.93, 719.94, 719.95, 719.96, 719.97, 719.98, 719.99, 718.4, 718.41, 718.42, 718.43, 718.44, 718.45, 718.46, 718.47, 718.48, 718.49, V43.60, V43.61, V43.62, V43.63, V43.64, V43.65, V43.66, V43.69, 755.63, 755.64, 718.3, 718.31, 718.32, 718.33, 718.34, 718.35, 718.36, 718.37, 718.38, 718.39 |
|  |  |  |  |  | Bone fracture | 808.4, 808.5, hipfx.1, 820., 820.00, 820.01, 820.02, 820.03, 820.09, 820.10, 820.11, 820.12, 820.13, 820.19, 820.20, 820.21, 820.22, 820.30, 820.31, 820.32, 820.8, 820.9, 905.3, V54.13, V54.23, 800.00, 800.01, 800.02, 800.03, 800.05, 800.06, 800.09, 800.50, 800.51, 800.52, 800.53, 800.55, 800.56, 800.59, 801.00, 801.01, 801.02, 801.03, 801.04, 801.05, 801.06, 801.09, 801.50, 801.51, 801.52, 801.53, 801.55, 801.56, 801.59, 802.0, 802.1, 802.20, 802.21, 802.22, 802.23, 802.24, 802.25, 802.26, 802.27, 802.28, 802.29, 802.3, 802.31, 802.32, 802.33, 802.34, 802.35, 802.36, 802.37, 802.38, 802.39, 802.4, 802.5, 802.6, 802.7, 802.8, 802.9, 803.00, 803.01, 803.02, 803.03, 803.04, 803.05, 803.06, 803.09, 803.50, 803.51, 803.52, 803.56, 803.59, 804.00, 804.01, 804.02, 804.03, 804.04, 804.05, 804.06, 804.09, 804.50, 804.51, 804.52, 804.53, 804.59, 905.0, 813.45, V54.10, V54.11, V54.12, V54.20, V54.21, V54.22, 812.00, 812.01, 812.02, 812.03, 812.09, 812.10, 812.11, 812.12, 812.13, 812.19, 812.20, 812.21, 812.30, 812.31, 812.4, 812.41, 812.42, 812.43, 812.44, 812.49, 812.5, 812.51, 812.52, 812.53, 812.54, 812.59, 813.0, 813.00, 813.01, 813.02, 813.03, 813.04, 813.05, 813.06, 813.07, 813.1, 813.10, 813.11, 813.12, 813.13, 813.14, 813.15, 813.16, 813.17, 813.2, 813.20, 813.21, 813.22, 813.3, 813.30, 813.31, 813.32, 813.4, 813.40, 813.41, 813.42, 813.43, 813.46, 813.47, 813.5, 813.50, 813.51, 813.52, 813.53, 813.8, 813.80, 813.81, 813.82, 813.9, 813.90, 813.91, 813.92, 810.0, 810.01, 810.02, 810.03, 810.1, 810.11, 810.12, 810.13, 811.0, 811.01, 811.02, 811.03, 811.09, 811.1, 811.11, 811.12, 811.13, 811.19, 814., 814.0, 814.01, 814.02, 814.03, 814.04, 814.05, 814.06, 814.07, 814.08, 814.09, 814.1, 814.11, 814.12, 814.13, 814.14, 814.15, 814.16, 814.17, 814.18, 814.19, 815.0, 815.01, 815.02, 815.03, 815.04, 815.09, 815.1, 815.11, 815.12, 815.13, 815.14, 815.19, 816.0, 816.01, 816.02, 816.03, 816.1, 816.11, 816.12, 816.13, 817.0, 817.1, 818.0, 818.1, 819.0, 819.1, 905.2, 823.40, 823.41, 823.42, V54.14, V54.15, V54.16, V54.24, V54.25, V54.26, 823.0, 823.00, 823.01, 823.1, 823.10, 823.11, 823.2, 823.20, 823.21, 823.3, 823.30, 823.31, 823.8, 823.80, 823.81, 823.9, 823.90, 823.91, 824., 824.0, 824.1, 824.2, 824.3, 824.4, 824.5, 824.6, 824.7, 824.8, 824.9, 821.00, 821.01, 821.10, 821.11, 821.20, 821.21, 821.22, 821.23, 821.29, 821.30, 821.31, 821.32, 821.33, 821.39, 822.0, 822.1, 825.0, 825.1, 825.2, 825.20, 825.21, 825.22, 825.23, 825.24, 825.25, 825.3, 825.30, 825.31, 825.32, 825.33, 825.34, 825.35, 826.0, 826.1, 827.0, 827.1, 905.4, V54.19, V54.29, V54.17, V54.27, 805.00, 805.01, 805.02, 805.03, 805.04, 805.05, 805.06, 805.07, 805.08, 805.10, 805.11, 805.12, 805.13, 805.14, 805.15, 805.16, 805.17, 805.18, 805.2, 805.3, 805.4, 805.5, 805.6, 805.7, 805.8, 805.9, 807.0, 807.00, 807.01, 807.02, 807.03, 807.04, 807.05, 807.06, 807.07, 807.08, 807.09, 808., 808.0, 808.1, 808.2, 808.3, 808.41, 808.42, 808.43, 808.51, 808.52, 808.53, 808.8, 808.9, 807.1, 807.11, 807.12, 807.13, 807.14, 807.16, 807.17, 807.18, 807.19, 807.2, 807.3, 807.4, 807.5, 807.6, 809.0, 809.1, 828.0, 828.1, 829., 829.0, 829.1, 905.1, 905.5, V54., V54.01, V54.02, V54.09, V66.4, V67.4 |
|  |  |  |  |  | Pathologic fracture | 733.1, 733.11, 733.12, 733.13, 733.14, 733.15, 733.16, 733.19 |
|  |  |  | * |  | Avascular necrosis | ostnec.1, 733.4, 733.41, 733.42, 733.43, 733.44, 733.45, 733.49 |
|  |  |  | * |  | Osteomyelitis | 730.0, 730.01, 730.02, 730.03, 730.04, 730.05, 730.06, 730.07, 730.08, 730.09, 730.1, 730.11, 730.12, 730.13, 730.14, 730.15, 730.16, 730.17, 730.18, 730.19, 730.2, 730.21, 730.22, 730.23, 730.24, 730.25, 730.26, 730.27, 730.28, 730.29 |
|  |  |  |  |  |  |  |
| GPR98  rs13157270 | Febrile seizures, familial | OMIM, KO mouse |  |  | Epilepsy | 345.00, 345.0, 345.01, 345.1, 345.10, 345.11, 345.2, 345.3, 345.4, 345.40, 345.41, 345.5, 345.50, 345.51, 345.60, 345.61, 345.70, 345.71, 345.80, 345.81, 345.9, 345.90, 345.91 |
|  |  |  |  |  | Febrile seizure | 780.31,780.32 |
|  |  |  |  |  | Convulsions | 780.3, 780.31, 780.32, 780.39 |
|  |  |  |  |  |  |  |
| HGFAC  rs16844401 | Impaired intestinal mucuosa healing | KO mouse | * |  | GI bleed/hematemesis | 578.0,578.9 |
|  |  |  |  |  | GI infections (bacterial) | 008.4, 001., 001.1, 002.0, 002.1, 002.2, 002.3, 002.9, 003.0, 003.2, 003.29, 003.8, 003.9, 004., 004.0, 004.1, 004.3, 004.8, 005., 005.0, 005.1, 005.2, 005.81, 005.9, 007.0, 007.1, 07.2, 007.3, 007.4, 007.8, 007.9, 008.00, 008.01, 008.02, 008.04, 008.09, 008.2, 008.3, 008.41, 008.42, 008.43, 008.44, 008.45, 008.46, 008.47, 008.5, 008.6, 008.61, 008.62, 008.63, 008.64, 008.67, 008.69, 008.8, 009.0, 009.1, 009.2, 009.3, 021.1, 022.2, 540., 540.0, 540.1, 540.9, 541., 542., 569.5 |
|  |  |  |  |  |  |  |
| IL2RA  rs2228149 | Grave's disease | OMIM, KO mouse, Association DB | * |  | Hyperthyroidism | 242.40, 242.41, 242., 242.0, 242.00, 242.01, 242.1, 242.10, 242.11, 242.2, 242.20, 242.21, 242.3, 242.30, 242.31, 242.8, 242.80, 242.81, 242.9, 242.90, 242.91 |
|  |  |  | * | * | All hypothyroidism (exclude 2ndary) | 244.0, 244.1, 244.3, 244.9 |
|  |  |  |  |  |  |  |
| INPP4B  rs34561493 | Osteoblast/clast dysfunction, osteoporosis | KO mouse |  |  | Osteoporosis | V17.81, 733.0, 733.00, 733.01, 733.02, 733.03, 733.09 |
|  |  |  |  |  | Bone fracture | 808.4, 808.5, hipfx.1, 820., 820.00, 820.01, 820.02, 820.03, 820.09, 820.10, 820.11, 820.12, 820.13, 820.19, 820.20, 820.21, 820.22, 820.30, 820.31, 820.32, 820.8, 820.9, 905.3, V54.13, V54.23, 800.00, 800.01, 800.02, 800.03, 800.05, 800.06, 800.09, 800.50, 800.51, 800.52, 800.53, 800.55, 800.56, 800.59, 801.00, 801.01, 801.02, 801.03, 801.04, 801.05, 801.06, 801.09, 801.50, 801.51, 801.52, 801.53, 801.55, 801.56, 801.59, 802.0, 802.1, 802.20, 802.21, 802.22, 802.23, 802.24, 802.25, 802.26, 802.27, 802.28, 802.29, 802.3, 802.31, 802.32, 802.33, 802.34, 802.35, 802.36, 802.37, 802.38, 802.39, 802.4, 802.5, 802.6, 802.7, 802.8, 802.9, 803.00, 803.01, 803.02, 803.03, 803.04, 803.05, 803.06, 803.09, 803.50, 803.51, 803.52, 803.56, 803.59, 804.00, 804.01, 804.02, 804.03, 804.04, 804.05, 804.06, 804.09, 804.50, 804.51, 804.52, 804.53, 804.59, 905.0, 813.45, V54.10, V54.11, V54.12, V54.20, V54.21, V54.22, 812.00, 812.01, 812.02, 812.03, 812.09, 812.10, 812.11, 812.12, 812.13, 812.19, 812.20, 812.21, 812.30, 812.31, 812.4, 812.41, 812.42, 812.43, 812.44, 812.49, 812.5, 812.51, 812.52, 812.53, 812.54, 812.59, 813.0, 813.00, 813.01, 813.02, 813.03, 813.04, 813.05, 813.06, 813.07, 813.1, 813.10, 813.11, 813.12, 813.13, 813.14, 813.15, 813.16, 813.17, 813.2, 813.20, 813.21, 813.22, 813.3, 813.30, 813.31, 813.32, 813.4, 813.40, 813.41, 813.42, 813.43, 813.46, 813.47, 813.5, 813.50, 813.51, 813.52, 813.53, 813.8, 813.80, 813.81, 813.82, 813.9, 813.90, 813.91, 813.92, 810.0, 810.01, 810.02, 810.03, 810.1, 810.11, 810.12, 810.13, 811.0, 811.01, 811.02, 811.03, 811.09, 811.1, 811.11, 811.12, 811.13, 811.19, 814., 814.0, 814.01, 814.02, 814.03, 814.04, 814.05, 814.06, 814.07, 814.08, 814.09, 814.1, 814.11, 814.12, 814.13, 814.14, 814.15, 814.16, 814.17, 814.18, 814.19, 815.0, 815.01, 815.02, 815.03, 815.04, 815.09, 815.1, 815.11, 815.12, 815.13, 815.14, 815.19, 816.0, 816.01, 816.02, 816.03, 816.1, 816.11, 816.12, 816.13, 817.0, 817.1, 818.0, 818.1, 819.0, 819.1, 905.2, 823.40, 823.41, 823.42, V54.14, V54.15, V54.16, V54.24, V54.25, V54.26, 823.0, 823.00, 823.01, 823.1, 823.10, 823.11, 823.2, 823.20, 823.21, 823.3, 823.30, 823.31, 823.8, 823.80, 823.81, 823.9, 823.90, 823.91, 824., 824.0, 824.1, 824.2, 824.3, 824.4, 824.5, 824.6, 824.7, 824.8, 824.9, 821.00, 821.01, 821.10, 821.11, 821.20, 821.21, 821.22, 821.23, 821.29, 821.30, 821.31, 821.32, 821.33, 821.39, 822.0, 822.1, 825.0, 825.1, 825.2, 825.20, 825.21, 825.22, 825.23, 825.24, 825.25, 825.3, 825.30, 825.31, 825.32, 825.33, 825.34, 825.35, 826.0, 826.1, 827.0, 827.1, 905.4, V54.19, V54.29, V54.17, V54.27, 805.00, 805.01, 805.02, 805.03, 805.04, 805.05, 805.06, 805.07, 805.08, 805.10, 805.11, 805.12, 805.13, 805.14, 805.15, 805.16, 805.17, 805.18, 805.2, 805.3, 805.4, 805.5, 805.6, 805.7, 805.8, 805.9, 807.0, 807.00, 807.01, 807.02, 807.03, 807.04, 807.05, 807.06, 807.07, 807.08, 807.09, 808., 808.0, 808.1, 808.2, 808.3, 808.41, 808.42, 808.43, 808.51, 808.52, 808.53, 808.8, 808.9, 807.1, 807.11, 807.12, 807.13, 807.14, 807.16, 807.17, 807.18, 807.19, 807.2, 807.3, 807.4, 807.5, 807.6, 809.0, 809.1, 828.0, 828.1, 829., 829.0, 829.1, 905.1, 905.5, V54., V54.01, V54.02, V54.09, V66.4, V67.4 |
|  |  |  |  |  | Pathologic fracture | 733.1, 733.11, 733.12, 733.13, 733.14, 733.15, 733.16, 733.19 |
|  |  |  | * |  | Ankle fracture | 824., 824.0, 824.1, 824.2, 824.3, 824.4, 824.5, 824.6, 824.7, 824.8, 824.9 |
|  |  |  |  |  |  |  |
| PLCG2  rs17537869 | Familial cold autoinflammatory syndrome | OMIM, KO mouse |  |  | Cold induced urticaria | 708.2 |
|  |  |  |  |  | Humoral immunity /Decreased IgA,IgM | 279.0, 279.00, 279.01, 279.02, 279.03 |
|  |  |  |  |  | Allergic reactions | 691.8, 693.0, 708.1, 477.1, 518.6, 558.3, 691.0, 692.8, 692.0, 692.1, 692.2, 692.3, 692.4, 692.5, 692.6, 692.70, 692.72, 692.73, 692.74, 692.79, 692.81, 692.82, 692.83, 692.84, 692.89, 692.9, 693., 693.1, 693.8, 708., 708.0, 708.2, 708.3, 708.4, 708.5, 708.9, 995.0, 995.3, 995.7, V07.1, V14.3, V14.4, V14.5, V14.7, V14.8, V14.9, V15.02, V15.05, V15.06, V15.07, V15.09, V72.7, V14.6, V14.2, V15.08 |
|  |  |  |  |  | Extrinsic asthma | 493.00,493.01, 493.02 |
|  |  |  |  |  | Allergic rhinits | 477., 477.0, 477.2, 477.8, 477.9 |
|  |  |  |  |  |  |  |
| PPP1R15B  rs2089891 | Anemia, Abnormal liver morphology | KO Mouse |  |  | Anemia | 280.1, 280.8, 280.9, 281.0, 281.4, 281.9, 280., 280.0, 283., 283.0, 283.1, 283.19, 283.11, 283.2, 283.9, 282., 282.2, 282.3, 282.44, 282.8, 284.2, 285.0, 285.2, 285.21, 285.22, 285.29, 285.8, 285.9 |
|  |  |  |  |  | Liver disease excluding cancer or infection | 571.4, 571.41, 571.42, 571.49, 573.1, 573.2, 573.3, 571.5, 572.1, 572.2, 572.3, 572.4, 572.8, 571.9, 571.6, 571.8, 570., 573.0, 782.4, 573.9, 573.4, 790.4, 790.5, 794.8, 789.1, 573.8 |
|  |  |  |  |  | Liver transplant | V42.7 |
|  |  |  |  |  |  |  |
| PPP1R3A  rs2974938 | Insulin resistance, excessive weight gain | OMIM, KO Mouse |  |  | DM2 | 250., 250.00, 250.02, 250.1, 250.10, 250.11, 250.12, 250.4, 250.40, 250.42, 250.50, 250.52, 250.6, 250.60, 250.62, 250.70, 250.72, 250.90, 250.2, 250.20, 250.22, 250.3, 250.30, 250.32, 250.8, 250.80, 250.82, 250.92 |
|  |  |  |  |  | Obesity | 278.01, 278.03, 278.00, 278.02 |
|  |  |  |  |  |  |  |
| PPP1R3A  rs2974942 | Insulin resistance, excessive weight gain | OMIM, KO Mouse |  |  | DM2 | 250., 250.00, 250.02, 250.1, 250.10, 250.11, 250.12, 250.4, 250.40, 250.42, 250.50, 250.52, 250.6, 250.60, 250.62, 250.70, 250.72, 250.90, 250.2, 250.20, 250.22, 250.3, 250.30, 250.32, 250.8, 250.80, 250.82, 250.92 |
|  |  |  |  |  | Obesity | 278.01, 278.03, 278.00, 278.02 |
|  |  |  |  |  |  |  |
| PTAFR  rs5939 | Streptococcus infection; infection susceptibility | KO mouse |  |  | Strep infections | 038.0, 041.01, 041.09, 041.0, 320.2, 482.31, 482.39, 034.0 |
|  |  |  |  |  | All sepsis | 038., 038.0, 038.1, 038.11, 038.12, 038.19, 038.4, 038.40, 038.41, 038.43, 038.44, 003.1, 020.2, 022.3, 036.2, 038.2, 038.3, 054.5, 038.42, 038.8, 038.9, 771.81, 790.7, 670.2, 670.22, 995.9, 995.91, 995.92, 771.81, 449. |
|  |  |  |  |  | Gram positive sepsis | 038., 038.0, 038.1, 038.11, 038.12, 038.19, 036.2, 038.2 |
|  |  |  |  |  | Gram negative sepsis | 038.4, 038.40, 038.41, 038.43, 038.44, 003.1, 020.2, 022.3, 054.5, 038.42, 038.3 |
|  |  |  |  |  | Acute upper respiratory infection | 465.9,465.8 |
|  |  |  |  |  | Sinusitis | 473., 473.0, 473.1, 473.2, 473.3, 473.8, 473.9, 461., 461.0, 461.1, 461.2, 461.3, 461.8 |
|  |  |  |  |  | Acute sinusitis | 461., 461.0, 461.1, 461.2, 461.3, 461.8 |
|  |  |  |  |  | Chronic sinusitis | 473., 473.0, 473.1, 473.2, 473.3, 473.8, 473.9 |
|  |  |  | * |  | Bacterial meningitis | 003.21, 036., 036.0, 047.0, 047.1, 072.1, 100.81, 114.2, 320.0, 320.1, 320.2, 320.3, 320.7, 320.8, 320.81, 320.82, 320.9, 321.0, 322., 322.0, 322.1, 322.2, 322.9 |
|  |  |  |  |  |  |  |
| PTGS1 rs1236913 |  | KO mouse |  |  | Other coagulation defects | 286., 286.0, 286.1, 286.2, 286.3, 286.4, 286.6, 286.7, 286.9, 289.81, 289.82 |
|  |  |  | * | * | Arterial thrombosis | 434.00, 445.01, 445.02, 445.81, 445.89, 444.22, 444.01, 444.09, 444.1, 444.21, 444.81, 444.89, 444.9 |
|  |  |  | * | * | Venous thrombosis | 452., 453.0, 453.1, 453.2, 453.3, 453.40, 453.41, 453.42, 453.50, 453.51, 453.52, 453.71, 453.72, 453.73, 453.74, 453.75, 453.76, 453.77, 453.79, 453.82, 453.83, 453.84, 453.85, 453.86, 453.87, 453.89, 453.9, V12.51 |
|  |  |  | * | * | Anti-coagulant use | V58.61 |
|  |  |  |  |  |  |  |
| SELP  rs3917724 | Dysfunctional platelet adhesion | OMIM |  |  | Spontaneous ecchymoses | 782.7 |
|  |  |  |  |  | Menstrual bleeding (women only) | 626., 626.2, 626.3, 626.5, 626.6, 626.8, 626.9,621.4,623.8,626.7,629.0 |
|  |  |  |  |  | Hematuria | 599.7,599.71,599.72 |
|  |  |  |  |  | GI bleed/Hematemesis | 578.1,578.,578.0,578.9 |
|  |  |  |  |  |  |  |
| SELP  rs6125 | Dysfunctional platelet adhesion | OMIM |  |  | Spontaneous ecchymoses | 782.7 |
|  |  |  |  |  | Menstrual bleeding (women only) | 626., 626.2, 626.3, 626.5, 626.6, 626.8, 626.9,621.4,623.8,626.7,629.0 |
|  |  |  |  |  | Hematuria | 599.7,599.71,599.72 |
|  |  |  |  |  | GI bleed/Hematemesis | 578.1,578.,578.0,578.9 |
|  |  |  |  |  |  |  |
| TAAR1  rs8192619 | Increased NE/dopamine, abnormal prepulse inhibition | KO mouse |  |  | Depression | 296.2, 296.20, 296.21, 296.22, 296.23, 296.24, 296.25, 296.26, 296.3, 296.31, 296.32, 296.33, 296.34, 296.35, 296.36, 300.4, 311. |
|  |  |  |  |  | Schizophrenia | 295.0, 295.01, 295.02, 295.03, 295.04, 295.05, 295.1, 295.10, 295.11, 295.12, 295.13, 295.14, 295.15, 295.2, 295.20, 295.21, 295.22, 295.23, 295.24, 295.25, 295.3, 295.31, 295.32, 295.33, 295.34, 295.35, 295.4, 295.40, 295.41, 295.42, 295.43, 295.44, 295.45, 295.5, 295.51, 295.52, 295.53, 295.54, 295.55, 295.60, 295.61, 295.62, 295.63, 295.64, 295.65, 295.7, 295.70, 295.71, 295.72, 295.73, 295.74, 295.75, 295.8, 295.81, 295.82, 295.83, 295.84, 295.85, 295.9, 295.91, 295.92, 295.93, 295.94, 295.95, V11.0 |
|  |  |  |  |  | Anxiety | 309.24, 309.28, 293.84, 300.0, 300.09, 300.02, 300.00, 300.01, 300.21, 300.10, 308., 308.3, 308.4, 313.0 |
|  |  |  |  |  |  |  |
| VWF  rs7962217 | Von Willebrands disease | OMIM | * | * | Venous thrombosis | 452., 453.0, 453.1, 453.2, 453.3, 453.40, 453.41, 453.42, 453.50, 453.51, 453.52, 453.71, 453.72, 453.73, 453.74, 453.75, 453.76, 453.77, 453.79, 453.82, 453.83, 453.84, 453.85, 453.86, 453.87, 453.89, 453.9, V12.51 |
