# Supplementary material for: Integrating EMR-Linked and In Vivo Functional Genetic Data to Identify New Genotype-Phenotype Associations
Source: PLoS One. 2014 Jun 20;9(6):e100322. doi: 10.1371/journal.pone.0100322 (PMC4065041; doi:10.1371/journal.pone.0100322)
Supplement: Table S4 — Diagnoses associated with SNP rs33947968 in the Myo3A gene. (DOCX) [file pone.0100322.s004.docx]

**Supplemental table 4. Diagnoses associated with SNP rs33947968 in the *Myo3A* gene.** Shown are all diagnoses associated with the SNP with a Fisher’s p-value<0.05.

| **Problem** | **Total homozygotes for minor allele** | **Affected minor allele homozygotes** | **Total homozygotes for common allele** | **Affected common allele homozygotes** | **Fisher's P-value** |
| --- | --- | --- | --- | --- | --- |
| Pulmonic incompetence NOS | 30 | 4 | 3263 | 21 | 0.00006 |
| Laennec's cirrhosis | 30 | 4 | 3263 | 35 | 0.00037 |
| Cardiac complications | 30 | 9 | 3263 | 256 | 0.00039 |
| Unspecified congenital anomaly of heart | 30 | 5 | 3263 | 69 | 0.00046 |
| Aortic valve stenosis | 30 | 8 | 3263 | 213 | 0.00058 |
| Myxoid transformation of mitral valve | 30 | 10 | 3263 | 376 | 0.0015 |
| Cardiac dysrhythmias | 30 | 11 | 3263 | 482 | 0.0028 |
| Tricuspid valve disorders, specified as nonrheumatic | 30 | 5 | 3263 | 125 | 0.0057 |
| Liver replaced by transplant | 30 | 5 | 3263 | 144 | 0.010 |
| Complications of the puerperium | 30 | 2 | 3263 | 16 | 0.011 |
| Heart replaced by transplant | 30 | 6 | 3263 | 217 | 0.014 |
| Right heart failure | 30 | 13 | 3263 | 735 | 0.014 |
| Codes related to substance-related disorders | 30 | 13 | 3263 | 736 | 0.014 |
| Other disorders of soft tissues | 30 | 3 | 3263 | 53 | 0.014 |
| Atrial flutter | 30 | 5 | 3263 | 158 | 0.015 |
| Other and ill-defined heart disease | 30 | 3 | 3263 | 57 | 0.016 |
| Other hypertension in pregnancy | 30 | 2 | 3263 | 20 | 0.017 |
| Primary idiopathic dilated cardiomyopathy | 30 | 7 | 3263 | 305 | 0.019 |
| Acute posthemorrhagic anemia | 30 | 14 | 3263 | 852 | 0.020 |
| Cancer of stomach | 30 | 2 | 3263 | 23 | 0.021 |
| Other screening for suspected conditions | 30 | 17 | 3263 | 1163 | 0.021 |
| Effusion of joint | 30 | 5 | 3263 | 175 | 0.022 |
| Heart valve replaced by transplant | 30 | 5 | 3263 | 176 | 0.022 |
| Primary pulmonary hypertension | 30 | 4 | 3263 | 120 | 0.025 |
| Cor pulmonale | 30 | 7 | 3263 | 324 | 0.026 |
| Chronic rheumatic disease of the heart valves | 30 | 5 | 3263 | 186 | 0.027 |
| Occlusion of cerebral arteries | 30 | 7 | 3263 | 330 | 0.028 |
| Syndrome, tachycardia-bradycardia | 30 | 4 | 3263 | 126 | 0.029 |
| Systolic hrt failure | 30 | 6 | 3263 | 261 | 0.030 |
| Cholelithiasis with other cholecystitis | 30 | 3 | 3263 | 75 | 0.033 |
| LBBB | 30 | 3 | 3263 | 77 | 0.035 |
| Mycotic aneurysm | 30 | 3 | 3263 | 77 | 0.035 |
| Neoplasm of uncertain behavior of skin | 30 | 6 | 3263 | 272 | 0.036 |
| Cardiac and circulatory congenital anomalies | 30 | 2 | 3263 | 34 | 0.042 |
| Hemothorax | 30 | 2 | 3263 | 36 | 0.046 |
| Coronary atherosclerosis | 30 | 14 | 3263 | 965 | 0.046 |
| Other biliary tract disease | 30 | 4 | 3263 | 150 | 0.049 |
