# Supplementary material for: Integrating EMR-Linked and In Vivo Functional Genetic Data to Identify New Genotype-Phenotype Associations
Source: PLoS One. 2014 Jun 20;9(6):e100322. doi: 10.1371/journal.pone.0100322 (PMC4065041; doi:10.1371/journal.pone.0100322)
Supplement: Table S5 — Association testing results for SNPs excluded during the review process. (DOCX) [file pone.0100322.s005.docx]

**Supplemental table 5. Association testing results for SNPs excluded during the review process.** Shown are the subject counts and results of exact logistic regression analyses comparing minor allele homozygotes to matched common allele homozygotes for the 13 of the 25 SNPs that were excluded during the SNP review process. The common allele homozygotes were matched for age, race, gender and data set.

| **Gene / SNP** | **Phenotypes** | **Total minor allele homozygotes** | **Affected minor allele homozygotes** | **Proportion affected** | **Total common allele homozygotes** | **Affected common allele homozygotes** | **Proportion affected** | **OR** | **95% CI** | **p-value** |
| --- | --- | --- | --- | --- | --- | --- | --- | --- | --- | --- |
| ADAMTS13  rs28647808 | Thrombocytopenia | 29 | 4 | 0.14 | 1084 | 197 | 0.18 | 0.7 | (0.2 - 2.1) | 0.5472 |
|  |  |  |  |  |  |  |  |  |  |  |
| CLEC1B  rs2273987 | Intracranial hemorrhage | 30 | 5 | 0.17 | 1480 | 56 | 0.04 | 5.1 | (1.9 - 13.8) | 0.0014 |
|  |  |  |  |  |  |  |  |  |  |  |
| CACNA2D4  rs2286372 | Retinal disease | 17 | 5 | 0.29 | 1261 | 169 | 0.13 | 2.5 | (0.9 - 7.2) | 0.0844 |
|  | Retinal disease (exlude HTN and DM) | 17 | 1 | 0.06 | 1261 | 83 | 0.07 | 0.9 | (0.1 - 6.9) | 0.9186 |
|  |  |  |  |  |  |  |  |  |  |  |
| DNAH5  rs2277046 | Situs inversus | 20 | 0 | 0.00 | 1470 | 0 | 0.00 | n/a |  |  |
|  | URI | 20 | 6 | 0.30 | 1470 | 171 | 0.12 | 3.0 | (1.2 - 8.0) | 0.0248 |
|  | Sinusitis | 20 | 8 | 0.40 | 1470 | 240 | 0.16 | 3.4 | (1.4 - 8.4) | 0.0078 |
|  | Chronic sinusitis | 20 | 4 | 0.20 | 1470 | 130 | 0.09 | 2.6 | (0.8 - 7.8) | 0.0945 |
|  | Pneumonia (bacterial) | 20 | 0 | 0.00 | 1470 | 136 | 0.09 | n/a |  |  |
|  | Bronchiectasis | 20 | 0 | 0.00 | 1470 | 5 | 0.00 | n/a |  |  |
|  | Other pulmonary: Pleurodynia | 20 | 7 | 0.35 | 1470 | 59 | 0.04 | 12.9 | (5.0 - 33.5) | <.0001 |
|  |  |  |  |  |  |  |  |  |  |  |
| IL2RA  rs2228149 | Hypothyroidism (excluding secondary) | 15 | 7 | 0.47 | 1432 | 190 | 0.13 | 5.7 | (2.1 - 16.0) | 0.0009 |
|  | Hyperthyroidism | 15 | 1 | 0.07 | 1432 | 39 | 0.03 | 2.6 | (0.3 - 19.9) | 0.3714 |
|  |  |  |  |  |  |  |  |  |  |  |
| INPP4B  rs34561493 | Ankle fracture | 27 | 7 | 0.26 | 756 | 42 | 0.06 | 6.0 | (2.4 - 14.9) | 0.0001 |
|  | Pathologic fracture | 27 | 3 | 0.11 | 756 | 39 | 0.05 | 2.3 | (0.7 - 8.0) | 0.189 |
|  | Osteoporosis | 27 | 10 | 0.37 | 756 | 208 | 0.28 | 1.6 | (0.7 - 3.4) | 0.2813 |
|  | Bone fracture | 27 | 8 | 0.30 | 756 | 195 | 0.26 | 1.2 | (0.5 - 2.8) | 0.6554 |
|  |  |  |  |  |  |  |  |  |  |  |
| PPP1R3A  rs2974938 | DM2 | 12 | 5 | 0.42 | 896 | 374 | 0.42 | 1.0 | (0.3 - 3.2) | 1 |
|  | Obesity | 12 | 6 | 0.50 | 896 | 283 | 0.32 | 2.2 | (0.7 - 6.8) | 0.1841 |
|  |  |  |  |  |  |  |  |  |  |  |
| PPP1R3A  rs2974942 | DM2 | 16 | 6 | 0.38 | 960 | 383 | 0.40 | 0.9 | (0.3 - 2.5) | 0.8477 |
|  | Obesity | 16 | 7 | 0.44 | 960 | 291 | 0.30 | 1.8 | (0.7 - 4.8) | 0.25 |
|  |  |  |  |  |  |  |  |  |  |  |
| PPP1R15B  rs2089891 | Anemia | 40 | 30 | 0.75 | 1503 | 817 | 0.54 | 2.5 | (1.2 - 5.2) | 0.0122 |
|  | Liver disease excluding cancer or infection | 40 | 17 | 0.43 | 1503 | 401 | 0.27 | 2.0 | (1.1 - 3.8) | 0.0292 |
|  | Liver transplant | 40 | 5 | 0.13 | 1503 | 67 | 0.04 | 3.1 | (1.2 - 8.1) | 0.0235 |
|  |  |  |  |  |  |  |  |  |  |  |
| PTGS1  rs1236913 | Venous thrombosis | 40 | 11 | 0.28 | 1520 | 166 | 0.11 | 3.1 | (1.5 - 6.3) | 0.0019 |
|  | Coagulation defects | 40 | 7 | 0.18 | 1520 | 103 | 0.07 | 2.9 | (1.3 - 6.8) | 0.0124 |
|  | Arterial thrombosis | 40 | 4 | 0.10 | 1520 | 60 | 0.04 | 2.7 | (0.9 - 7.8) | 0.0671 |
|  | Anti-coagulant use | 40 | 8 | 0.20 | 1520 | 226 | 0.15 | 1.4 | (0.7 - 3.1) | 0.3712 |
|  |  |  |  |  |  |  |  |  |  |  |
| SELP  rs3917724 | Spontaneous ecchymoses | 12 | 0 | 0.00 | 820 | 2 | 0.00 | n/a |  |  |
|  | Menstrual bleeding (women only) | 10 | 4 | 0.40 | 660 | 131 | 0.20 | 2.7 | (0.7 - 9.7) | 0.1293 |
|  | Hematuria | 12 | 0 | 0.00 | 820 | 79 | 0.10 | n/a |  |  |
|  | GI bleed/Hematemesis | 12 | 2 | 0.17 | 820 | 76 | 0.09 | 2.0 | (0.4 - 9.1) | 0.3914 |
|  |  |  |  |  |  |  |  |  |  |  |
| SELP  rs6125 | Spontaneous ecchymoses | 26 | 2 | 0.08 | 1508 | 9 | 0.01 | 13.9 | (2.8 - 67.7) | 0.0011 |
|  | Hematuria | 26 | 7 | 0.27 | 1508 | 193 | 0.13 | 2.5 | (1.0 - 6.1) | 0.0403 |
|  | GI bleed/Hematemesis | 26 | 5 | 0.19 | 1508 | 150 | 0.10 | 2.2 | (0.8 - 5.8) | 0.1282 |
|  | Menstrual bleeding (women only) | 12 | 3 | 0.25 | 696 | 93 | 0.13 | 2.2 | (0.6 - 8.1) | 0.2542 |
|  |  |  |  |  |  |  |  |  |  |  |
| VWF  rs7962217 | Venous thrombosis | 17 | 5 | 0.29 | 1213 | 187 | 0.15 | 2.3 | (0.8 - 6.6) | 0.1244 |
