# Supplementary material for: Integrating EMR-Linked and In Vivo Functional Genetic Data to Identify New Genotype-Phenotype Associations
Source: PLoS One. 2014 Jun 20;9(6):e100322. doi: 10.1371/journal.pone.0100322 (PMC4065041; doi:10.1371/journal.pone.0100322)
Supplement: Table S6 — SNPs excluded based on record review. (DOCX) [file pone.0100322.s006.docx]

**Supplemental table 6. SNPs excluded based on record review.** Shown are the 7 SNPs excluded after record review and the reasons their exclusion.

| **Gene** | **SNP** | **Phenotype description** | **Reason for exclusion** |
| --- | --- | --- | --- |
| CLEC1B | rs2273987 | Abnormal blood vessel morphology, intracranial hemorrhage | Intracranial bleeds were post-hemorrhagic. No evidence of blood vessel abnormalities (hemorrhagic, etc.) |
| DNAH5 | rs2277046 | Ciliary dyskinesia | No evidence of intrinsic pulmonary disease. Pleurodynia mostly attributed to secondary causes (trauma, surgery, etc.) |
| IL2RA | rs2228149 | Grave's disease | No evidence of immune involvement in thryoid disease. |
| INPP4B | rs34561493 | Osteoblast/clast dysfunction, osteoporosis | Most fracture related to trauma. No pathological fractures. |
| PPP1R15B | rs2089891 | Anemia, Abnormal liver morphology | Most liver cases related to secondary causes. Only 2 cases are idiopathic. |
| PTGS1 (COX1) | rs1236913 | Hemorrhage | Only 1 case of thrombus confirmed. |
| SELP | rs6125 | Dysfunctional platelet adhesion | No evidence of spontaneous or abnormal bleeding. |
