# Supplementary material for: Integrating EMR-Linked and In Vivo Functional Genetic Data to Identify New Genotype-Phenotype Associations
Source: PLoS One. 2014 Jun 20;9(6):e100322. doi: 10.1371/journal.pone.0100322 (PMC4065041; doi:10.1371/journal.pone.0100322)
Supplement: Table S7 — Shared diagnosis lists for the 12 selected SNPs. (DOCX) [file pone.0100322.s007.docx]

**Supplemental table 7. Shared diagnosis lists for the 12 selected SNPs.** For each SNP, only those diagnoses that were shared by 2 or more of the minor allele homozygotes, that had a Fisher’s exact p-value of less than 0.05 and that appeared on less than 5% of the 711 problem lists examined are shown. The numbers of affected and total subjects for the two groups of homozygotes are shown. All genotyped homozygous subjects are included in these analyses. The Fisher’s p-value represents the probability that the two groups have similar proportions of affected subjects. Highlighted in yellow are the problems that were felt to be consistent with the known function of the gene that contained the SNP. The numbers in the “Subject IDs” column identify the affected minor allele homozygotes. These IDs are re-assigned for each SNP, and cannot be compared across SNPs.

| **Gene** | **SNP** | **Diagnosis** | **Total homozygotes for minor allele** | **Affected minor allele homozygotes** | **Total homozygotes for common allele** | **Affected common allele homozygotes** | **Fisher's P-value** | **Subject IDs of the affected minor allele homozygotes** |
| --- | --- | --- | --- | --- | --- | --- | --- | --- |
| ADAM22 | rs17255978 | Postphlebitic syndrome | 28 | 3 | 4775 | 14 | 0.000114276 | 8,14,17 |
| ADAM22 | rs17255978 | Complications of pregnancy-Coagulation def | 28 | 2 | 4775 | 3 | 0.000324247 | 6,9 |
| ADAM22 | rs17255978 | Complications of the puerperium-Deep thrombophlebitis | 28 | 2 | 4775 | 9 | 0.001745137 | 6,9 |
| ADAM22 | rs17255978 | Hypoproconvertinemia | 28 | 2 | 4775 | 9 | 0.001745137 | 2,6 |
| ADAM22 | rs17255978 | Gastric ulcer | 28 | 4 | 4775 | 104 | 0.003265667 | 2,12,18,24 |
| ADAM22 | rs17255978 | Glaucoma-Open angle | 28 | 5 | 4775 | 188 | 0.004596483 | 2,8,11,14,24 |
| ADAM22 | rs17255978 | Other specified conditions originating in the perinatal period | 28 | 2 | 4775 | 18 | 0.005836314 | 19,25 |
| ADAM22 | rs17255978 | Phlebitis and thrombophlebitis | 28 | 6 | 4775 | 308 | 0.008218476 | 1,2,5,12,14,17 |
| ADAM22 | rs17255978 | Idiopathic progressive polyneuropathy | 28 | 7 | 4775 | 410 | 0.008387214 | 2,5,12,14,18,19,23 |
| ADAM22 | rs17255978 | Prolonged pregnancy | 28 | 2 | 4775 | 24 | 0.009770173 | 6,21 |
| ADAM22 | rs17255978 | Pap smear abnormalities | 28 | 4 | 4775 | 144 | 0.009963397 | 6,9,12,16 |
| ADAM22 | rs17255978 | Adjustment disorders | 28 | 5 | 4775 | 235 | 0.011419327 | 2,5,9,12,17 |
| ADAM22 | rs17255978 | Toxic megacolon | 28 | 2 | 4775 | 28 | 0.012890621 | 19,28 |
| ADAM22 | rs17255978 | Other eye disorders | 28 | 11 | 4775 | 919 | 0.013844251 | 1,2,3,8,9,11,12,14,15,17,18 |
| ADAM22 | rs17255978 | Backache; unspecified | 28 | 11 | 4775 | 949 | 0.016440182 | 2,4,5,6,8,9,12,13,14,18,19 |
| ADAM22 | rs17255978 | Spontaneous abortion | 28 | 2 | 4775 | 33 | 0.017318879 | 6,9 |
| ADAM22 | rs17255978 | Disorders of the peripheral nervous system | 28 | 10 | 4775 | 804 | 0.018433845 | 1,2,8,12,14,17,18,19,23,28 |
| ADAM22 | rs17255978 | Medical examination/evaluation | 28 | 16 | 4775 | 3728 | 0.019116654 | 1,2,3,5,6,7,8,9,11,12,14,16,18,19,21,23 |
| ADAM22 | rs17255978 | Disorders of iron metabolism | 28 | 2 | 4775 | 35 | 0.019247211 | 5,28 |
| ADAM22 | rs17255978 | Lumbago | 28 | 12 | 4775 | 1059 | 0.019545345 | 2,3,5,7,8,9,11,12,13,14,16,18 |
| ADAM22 | rs17255978 | Normal delivery | 28 | 2 | 4775 | 41 | 0.025542634 | 6,9 |
| ADAM22 | rs17255978 | Nonmalignant breast conditions | 28 | 7 | 4775 | 520 | 0.027994729 | 2,3,5,8,11,14,16 |
| ADAM22 | rs17255978 | Pulmonary heart disease | 28 | 4 | 4775 | 199 | 0.028620868 | 2,5,6,14 |
| ADAM22 | rs17255978 | Occlusion of cerebral arteries | 28 | 5 | 4775 | 302 | 0.030093934 | 2,8,15,17,23 |
| ADAM22 | rs17255978 | Anemia during pregnancy | 28 | 2 | 4775 | 45 | 0.030144168 | 9,21 |
| ADAM22 | rs17255978 | Other retinal disorders | 28 | 4 | 4775 | 205 | 0.031423328 | 5,8,11,17 |
| ADAM22 | rs17255978 | Perineal laceration | 28 | 2 | 4775 | 48 | 0.033796273 | 6,9 |
| ADAM22 | rs17255978 | Superficial injury; contusion | 28 | 9 | 4775 | 764 | 0.033955419 | 3,5,7,12,13,14,17,18,24 |
| ADAM22 | rs17255978 | Other male genital disorders-prostate | 28 | 2 | 4775 | 50 | 0.036323042 | 2,19 |
| ADAM22 | rs17255978 | Arteriovenous fistula, acquired | 28 | 2 | 4775 | 51 | 0.037613319 | 2,12 |
| ADAM22 | rs17255978 | Diabetic retinopathy | 28 | 5 | 4775 | 322 | 0.038125766 | 1,8,11,12,17 |
| ADAM22 | rs17255978 | Complications of other transplanted organ | 28 | 2 | 4775 | 55 | 0.042948255 | 7,19 |
| ADAM22 | rs17255978 | Unspecified erythematous condition | 28 | 3 | 4775 | 133 | 0.043417634 | 2,11,19 |
| ADAM22 | rs17255978 | Other and unspecified viral infection | 28 | 9 | 4775 | 818 | 0.044601116 | 2,3,4,6,7,9,17,19,25 |
| ADAM22 | rs17255978 | Other and unspecified complications of pregnancy | 28 | 2 | 4775 | 57 | 0.045716803 | 6,21 |
| ADAM22 | rs17255978 | Other inflammatory condition of skin-Seborrhea | 28 | 3 | 4775 | 136 | 0.045844514 | 3,4,19 |
| ADAM22 | rs17255978 | Personal history of allergy to other antibiotic agent | 28 | 2 | 4775 | 59 | 0.048550335 | 2,17 |
| AOC3 | rs33986943 | Other gram negative septicemia | 35 | 7 | 4683 | 134 | 6.11832E-05 | 2,10,16,21,26,28,31 |
| AOC3 | rs33986943 | Other bacterial infections | 35 | 15 | 4683 | 894 | 0.001669461 | 2,3,4,5,6,9,10,13,16,18,20,21,22,29,31 |
| AOC3 | rs33986943 | Other specified types of cystitis | 35 | 2 | 4683 | 8 | 0.002318005 | 2,31 |
| AOC3 | rs33986943 | Sepsis/SIRS | 35 | 12 | 4683 | 663 | 0.002362286 | 2,3,5,6,10,15,16,21,26,28,29,31 |
| AOC3 | rs33986943 | Hypotension | 35 | 15 | 4683 | 949 | 0.002432962 | 1,2,3,4,5,6,10,11,15,16,22,24,26,28,29 |
| AOC3 | rs33986943 | Allergic reactions-other | 35 | 4 | 4683 | 74 | 0.002453526 | 1,3,7,29 |
| AOC3 | rs33986943 | Fibrosclerosis of breast | 35 | 2 | 4683 | 9 | 0.002819947 | 5,35 |
| AOC3 | rs33986943 | Retention of urine | 35 | 9 | 4683 | 412 | 0.002836178 | 2,4,5,6,7,21,28,30,31 |
| AOC3 | rs33986943 | Other resistant bacterial infections | 35 | 9 | 4683 | 412 | 0.002836178 | 2,3,4,5,13,15,22,24,31 |
| AOC3 | rs33986943 | Mycotic aneurysm | 35 | 4 | 4683 | 80 | 0.003215911 | 3,11,14,16 |
| AOC3 | rs33986943 | Other transfusion reaction | 35 | 2 | 4683 | 10 | 0.003368211 | 16,29 |
| AOC3 | rs33986943 | Other bone disease and musculoskeletal deformities | 35 | 7 | 4683 | 273 | 0.003839471 | 2,3,5,10,12,20,34 |
| AOC3 | rs33986943 | SIRS-noninf | 35 | 3 | 4683 | 60 | 0.010957743 | 5,22,28 |
| AOC3 | rs33986943 | Other diseases of veins and lymphatics | 35 | 3 | 4683 | 61 | 0.011438797 | 3,11,12 |
| AOC3 | rs33986943 | Strep/staph septicemia | 35 | 11 | 4683 | 672 | 0.012318336 | 2,3,4,5,6,10,16,21,28,29,31 |
| AOC3 | rs33986943 | Communication disorders | 35 | 4 | 4683 | 121 | 0.013029474 | 2,4,16,28 |
| AOC3 | rs33986943 | Personality disorders | 35 | 3 | 4683 | 66 | 0.014026635 | 2,3,10 |
| AOC3 | rs33986943 | Depressive disorders | 35 | 17 | 4683 | 1365 | 0.015265096 | 1,2,3,4,5,6,7,9,14,15,16,20,22,24,29,30,35 |
| AOC3 | rs33986943 | Unspecified septicemia | 35 | 11 | 4683 | 712 | 0.015413031 | 2,3,4,5,10,16,18,20,21,22,29 |
| AOC3 | rs33986943 | Other specified disorders of biliary tract | 35 | 4 | 4683 | 129 | 0.016062115 | 16,20,21,22 |
| AOC3 | rs33986943 | Other and unspecified urinary calculus | 35 | 4 | 4683 | 129 | 0.016062115 | 2,6,12,31 |
| AOC3 | rs33986943 | Chronic obstructive asthma without status asthmaticus or exacerbation | 35 | 4 | 4683 | 130 | 0.016470024 | 1,7,11,15 |
| AOC3 | rs33986943 | Other infections; including parasitic | 35 | 8 | 4683 | 453 | 0.017208717 | 1,2,3,5,16,26,28,35 |
| AOC3 | rs33986943 | Cardiac arrest and ventricular fibrillation | 35 | 4 | 4683 | 132 | 0.017305432 | 1,3,21,22 |
| AOC3 | rs33986943 | Staph infections | 35 | 11 | 4683 | 739 | 0.018590138 | 3,4,5,13,15,16,18,20,22,30,31 |
| AOC3 | rs33986943 | Unspecified disorder of thyroid | 35 | 3 | 4683 | 74 | 0.018810272 | 5,6,9 |
| AOC3 | rs33986943 | Respiratory acidosis/alkalosis | 35 | 11 | 4683 | 741 | 0.018862704 | 1,4,5,6,15,16,19,21,22,26,28 |
| AOC3 | rs33986943 | Spondylosis and allied disorders | 35 | 8 | 4683 | 466 | 0.020082293 | 6,11,14,18,20,22,24,31 |
| AOC3 | rs33986943 | Diseases of the digestive system | 35 | 21 | 4683 | 1862 | 0.02283686 | ,35 |
| AOC3 | rs33986943 | Nephritis; nephrosis; renal sclerosis | 35 | 2 | 4683 | 30 | 0.023071162 | 9,16 |
| AOC3 | rs33986943 | Autoimmune disease, not elsewhere classified | 35 | 2 | 4683 | 30 | 0.023071162 | 9,11 |
| AOC3 | rs33986943 | Other complications of internal prosthetic device; implant; and graft | 35 | 12 | 4683 | 839 | 0.023854057 | 1,2,4,5,6,9,11,13,16,18,33,34 |
| AOC3 | rs33986943 | Splenomegaly | 35 | 4 | 4683 | 146 | 0.02390351 | 11,16,21,28 |
| AOC3 | rs33986943 | Adjustment disorders | 35 | 5 | 4683 | 221 | 0.024193899 | 2,3,4,6,35 |
| AOC3 | rs33986943 | Sequelae of chronic liver disease | 35 | 4 | 4683 | 150 | 0.02603608 | 6,16,20,21 |
| AOC3 | rs33986943 | Deficiency and other anemia | 35 | 12 | 4683 | 864 | 0.026284669 | 1,5,6,7,10,11,16,18,20,22,29,30 |
| AOC3 | rs33986943 | Osteoporosis | 35 | 12 | 4683 | 865 | 0.026401324 | 1,2,5,6,7,8,9,10,11,27,29,30 |
| AOC3 | rs33986943 | Idiopathic progressive polyneuropathy | 35 | 7 | 4683 | 399 | 0.026952554 | 1,2,4,9,16,19,31 |
| AOC3 | rs33986943 | Esophageal varices | 35 | 3 | 4683 | 89 | 0.029949503 | 16,20,21 |
| AOC3 | rs33986943 | Other specified hypotension | 35 | 3 | 4683 | 90 | 0.030793092 | 1,2,29 |
| AOC3 | rs33986943 | Volvulus of the small bowel | 35 | 2 | 4683 | 37 | 0.033373284 | 4,28 |
| AOC3 | rs33986943 | Bladder neck obstruction | 35 | 2 | 4683 | 38 | 0.034968586 | 2,16 |
| AOC3 | rs33986943 | Complications of transplanted pancreas | 35 | 2 | 4683 | 40 | 0.038246782 | 6,27 |
| AOC3 | rs33986943 | Fever of unknown origin | 35 | 11 | 4683 | 800 | 0.039120599 | 3,5,8,13,16,28,29,30,31,33,34 |
| AOC3 | rs33986943 | Staghorn calculus | 35 | 6 | 4683 | 340 | 0.039489189 | 2,6,12,20,24,31 |
| AOC3 | rs33986943 | Postoperative infection | 35 | 9 | 4683 | 609 | 0.039584472 | 3,4,5,20,22,28,30,33,34 |
| AOC3 | rs33986943 | Purpura, fulminans | 35 | 2 | 4683 | 41 | 0.039928689 | 11,26 |
| AOC3 | rs33986943 | Kidney replaced by transplant | 35 | 11 | 4683 | 807 | 0.040222143 | 5,6,7,8,9,11,16,19,24,26,30 |
| AOC3 | rs33986943 | Emphysema | 35 | 5 | 4683 | 264 | 0.046327284 | 10,11,15,18,20 |
| AOC3 | rs33986943 | Other skin disorders | 35 | 3 | 4683 | 108 | 0.048104994 | 3,4,5 |
| AOC3 | rs33986943 | Other malnutrition | 35 | 8 | 4683 | 505 | 0.048315026 | 4,10,13,16,22,28,29,35 |
| CACNA1A | rs16027 | Female genital disorders-fistula | 54 | 3 | 7339 | 29 | 0.001573023 | 16,26,37 |
| CACNA1A | rs16027 | Delayed milestones | 54 | 3 | 7339 | 35 | 0.00259402 | 22,25,51 |
| CACNA1A | rs16027 | Migraine | 54 | 10 | 7339 | 530 | 0.005095285 | 1,3,5,9,21,29,33,40,43,44 |
| CACNA1A | rs16027 | Other non-traumatic joint disorders | 54 | 5 | 7339 | 173 | 0.009325629 | 8,9,22,23,32 |
| CACNA1A | rs16027 | Toxic erythema/TEN/SJS | 54 | 2 | 7339 | 19 | 0.010062001 | 5,27 |
| CACNA1A | rs16027 | Endometriosis | 54 | 4 | 7339 | 122 | 0.013193621 | 1,6,10,33 |
| CACNA1A | rs16027 | Pyoderma gangrenosum | 54 | 2 | 7339 | 23 | 0.014109197 | 30,37 |
| CACNA1A | rs16027 | Unspecified diseases of blood and blood-forming organs | 54 | 4 | 7339 | 126 | 0.014656657 | 10,22,30,51 |
| CACNA1A | rs16027 | Primary dysmenorrhea | 54 | 4 | 7339 | 141 | 0.021044672 | 1,6,10,29 |
| CACNA1A | rs16027 | Cystitis | 54 | 6 | 7339 | 306 | 0.025334815 | 1,9,24,26,33,42 |
| CACNA1A | rs16027 | Pelvic peritoneal adhesions, female (postoperative) (postinfection) | 54 | 3 | 7339 | 89 | 0.02930808 | 1,6,49 |
| CACNA1A | rs16027 | Sec hypercoagulable st | 54 | 2 | 7339 | 35 | 0.029628046 | 37,49 |
| CACNA1A | rs16027 | Convulsions | 54 | 10 | 7339 | 681 | 0.030987944 | 2,7,10,13,22,24,29,32,40,41 |
| CACNA1A | rs16027 | Other esophageal disorders | 54 | 25 | 7339 | 2391 | 0.040623489 | ,41,42,43,48,51 |
| CACNA1A | rs16027 | Lupus erythematosus | 54 | 2 | 7339 | 46 | 0.047701455 | 8,33 |
| CACNA1A | rs16027 | Polymyalgia rheumatica | 54 | 2 | 7339 | 46 | 0.047701455 | 5,11 |
| CACNA1A | rs16027 | Anal and rectal conditions | 54 | 3 | 7339 | 109 | 0.048079919 | 6,37,45 |
| CNGB3 | rs3735972 | Inflammation; infection of eye (except that caused by TB or STD) | 74 | 18 | 7188 | 790 | 0.001132707 | 2,3,6,7,9,13,22,23,27,28,30,33,36,41,45,48,54,60 |
| CNGB3 | rs3735972 | Other eye disorders | 74 | 24 | 7188 | 1252 | 0.001793065 | ,51,52,54,67,68 |
| CNGB3 | rs3735972 | Cataract | 74 | 18 | 7188 | 845 | 0.00299334 | 1,3,6,7,9,13,18,22,23,27,30,34,36,38,44,45,48,54 |
| CNGB3 | rs3735972 | Burns | 74 | 5 | 7188 | 98 | 0.003866211 | 2,13,36,37,44 |
| CNGB3 | rs3735972 | Gastrointestinal complications | 74 | 6 | 7188 | 158 | 0.006319917 | 5,25,34,40,51,56 |
| CNGB3 | rs3735972 | Retroperitoneal hemorrhage | 74 | 3 | 7188 | 35 | 0.006635463 | 1,5,43 |
| CNGB3 | rs3735972 | Gangrene | 74 | 5 | 7188 | 121 | 0.009007388 | 13,22,31,38,72 |
| CNGB3 | rs3735972 | Other thyroid disorders | 74 | 9 | 7188 | 350 | 0.010271226 | 1,4,7,13,14,36,42,60,66 |
| CNGB3 | rs3735972 | Cellulitis and abscess of fingers and toes | 74 | 6 | 7188 | 186 | 0.013202395 | 2,13,14,17,29,31 |
| CNGB3 | rs3735972 | Feeding difficulties and mismanagement | 74 | 12 | 7188 | 568 | 0.01558308 | 5,13,17,21,28,36,55,57,64,72,73,74 |
| CNGB3 | rs3735972 | Other inflammatory condition of skin | 74 | 11 | 7188 | 477 | 0.015593175 | 1,7,9,13,14,15,27,32,39,61,71 |
| CNGB3 | rs3735972 | Atherosclerosis of arteries of extremities | 74 | 9 | 7188 | 377 | 0.01594928 | 3,9,13,22,29,31,36,38,48 |
| CNGB3 | rs3735972 | Myasthenia gravis | 74 | 2 | 7188 | 20 | 0.020744408 | 22,67 |
| CNGB3 | rs3735972 | Other and unspecified diseases of ureters | 74 | 6 | 7188 | 1325 | 0.022328656 | 12,13,22,26,31,48 |
| CNGB3 | rs3735972 | Umbilical hernia with obstruction/gangrene | 74 | 11 | 7188 | 533 | 0.024056549 | 21,25,27,29,32,33,40,48,59,63,73 |
| CNGB3 | rs3735972 | Fibroadenosis of breast | 74 | 2 | 7188 | 22 | 0.024463829 | 6,27 |
| CNGB3 | rs3735972 | Other nervous system symptoms and disorders | 74 | 34 | 7188 | 2361 | 0.024485786 | ,42,43,44,50,52,54,55,57,58,62,64,65,73,74 |
| CNGB3 | rs3735972 | Thrombocytopenic purpura | 74 | 2 | 7188 | 23 | 0.026418188 | 28,64 |
| CNGB3 | rs3735972 | Neutropenia | 74 | 10 | 7188 | 466 | 0.028219188 | 6,14,22,23,29,39,40,58,61,64 |
| CNGB3 | rs3735972 | Sprains and strains | 74 | 18 | 7188 | 1050 | 0.029935685 | 1,2,3,4,5,6,7,13,17,24,25,27,30,36,49,52,54,55 |
| CNGB3 | rs3735972 | Nephrogenic diabetes insipidus | 74 | 2 | 7188 | 25 | 0.030509028 | 12,27 |
| CNGB3 | rs3735972 | Eosinophilia | 74 | 3 | 7188 | 67 | 0.034185541 | 1,33,49 |
| CNGB3 | rs3735972 | Tietze's disease | 74 | 3 | 7188 | 68 | 0.035439221 | 2,13,49 |
| CNGB3 | rs3735972 | Nevus, non-neoplastic | 74 | 3 | 7188 | 69 | 0.036716503 | 7,9,27 |
| CNGB3 | rs3735972 | Tuberculosis-Other organs | 74 | 3 | 7188 | 70 | 0.038017305 | 4,19,51 |
| CNGB3 | rs3735972 | Hemorrhage NOS | 74 | 4 | 7188 | 125 | 0.04215338 | 1,23,28,72 |
| CNGB3 | rs3735972 | Open wounds of extremities | 74 | 12 | 7188 | 658 | 0.043555732 | 1,2,5,7,8,13,22,30,31,32,38,55 |
| CNGB3 | rs3735972 | Spinal stenosis; lumbar region | 74 | 9 | 7188 | 443 | 0.047552723 | 3,4,6,7,42,48,54,55,58 |
| ERCC4 | rs1800067 | Other retinal disorders | 42 | 9 | 7640 | 391 | 0.000247083 | 2,4,7,13,14,16,25,29,31 |
| ERCC4 | rs1800067 | Chronic ulcer of leg or foot | 42 | 7 | 7640 | 311 | 0.001507145 | 1,7,9,13,14,17,29 |
| ERCC4 | rs1800067 | Hyperplasia of prostate | 42 | 8 | 7640 | 431 | 0.002255334 | 2,4,6,16,19,20,24,29 |
| ERCC4 | rs1800067 | Hematuria | 42 | 12 | 7640 | 933 | 0.003654464 | 1,2,3,4,5,13,15,16,20,29,32,40 |
| ERCC4 | rs1800067 | Other peripheral and visceral atherosclerosis | 42 | 8 | 7640 | 485 | 0.004617176 | 1,8,13,16,19,21,29,33 |
| ERCC4 | rs1800067 | Toxic erythema/TEN/SJS | 42 | 2 | 7640 | 19 | 0.005737825 | 2,31 |
| ERCC4 | rs1800067 | Other skin disorders-Keratosis | 42 | 9 | 7640 | 638 | 0.007182229 | 2,4,6,8,9,11,13,20,37 |
| ERCC4 | rs1800067 | Other specified cystic kidney disease | 42 | 3 | 7640 | 70 | 0.00724799 | 3,8,23 |
| ERCC4 | rs1800067 | GI Fistula/ulceration | 42 | 4 | 7640 | 141 | 0.007815468 | 5,25,33,39 |
| ERCC4 | rs1800067 | Hypotension | 42 | 13 | 7640 | 1141 | 0.007832413 | 1,3,5,6,13,16,21,25,29,33,34,35,39 |
| ERCC4 | rs1800067 | Strep infections | 42 | 7 | 7640 | 434 | 0.009196931 | 2,3,7,13,15,16,31 |
| ERCC4 | rs1800067 | Thrombocytopenia, unspecified | 42 | 10 | 7640 | 787 | 0.009216226 | 1,3,9,12,15,19,29,31,33,34 |
| ERCC4 | rs1800067 | Thrombocytopenic purpura | 42 | 2 | 7640 | 25 | 0.009393741 | 9,19 |
| ERCC4 | rs1800067 | Scondary DM | 42 | 2 | 7640 | 25 | 0.009393741 | 11,13 |
| ERCC4 | rs1800067 | Other connective tissue disease-amputation | 42 | 4 | 7640 | 149 | 0.009409479 | 7,9,13,24 |
| ERCC4 | rs1800067 | Cystic fibrosis | 42 | 3 | 7640 | 81 | 0.010652878 | 11,15,21 |
| ERCC4 | rs1800067 | Inguinal hernia with obstruction or gangrene | 42 | 2 | 7640 | 27 | 0.010791029 | 6,8 |
| ERCC4 | rs1800067 | Other bacterial pneumonia | 42 | 16 | 7640 | 1610 | 0.012308834 | 1,2,3,9,11,13,14,15,16,20,21,25,29,31,33,34 |
| ERCC4 | rs1800067 | Pancreatic insufficiency | 42 | 4 | 7640 | 162 | 0.012429282 | 5,11,12,32 |
| ERCC4 | rs1800067 | Other and unspecified diseases of kidney | 42 | 9 | 7640 | 717 | 0.014792359 | 1,3,4,13,14,15,18,21,33 |
| ERCC4 | rs1800067 | Other peripheral vascular disease | 42 | 3 | 7640 | 98 | 0.017478014 | 1,29,35 |
| ERCC4 | rs1800067 | Glomerulonephritis/Insterstitial nephritis | 42 | 3 | 7640 | 98 | 0.017478014 | 13,16,25 |
| ERCC4 | rs1800067 | Leukocytosis NOS | 42 | 10 | 7640 | 821 | 0.02027763 | 1,5,6,9,12,15,21,25,29,33 |
| ERCC4 | rs1800067 | Foot deformities | 42 | 2 | 7640 | 40 | 0.021884006 | 9,14 |
| ERCC4 | rs1800067 | Other bacterial infections | 42 | 11 | 7640 | 1026 | 0.02282514 | 1,3,5,7,13,15,16,23,25,31,34 |
| ERCC4 | rs1800067 | Cholecystitis without cholelithiasis | 42 | 4 | 7640 | 202 | 0.025387725 | 6,12,15,33 |
| ERCC4 | rs1800067 | Peritonitis and intestinal abscess | 42 | 5 | 7640 | 304 | 0.025633573 | 5,13,25,33,39 |
| ERCC4 | rs1800067 | Screening for other disorders of blood and blood-forming organs | 42 | 2 | 7640 | 44 | 0.025948308 | 3,15 |
| ERCC4 | rs1800067 | Disorders of the peripheral nervous system | 42 | 12 | 7640 | 1132 | 0.026020193 | 1,5,7,8,9,14,16,25,29,32,37,42 |
| ERCC4 | rs1800067 | Spasm of artery | 42 | 7 | 7640 | 535 | 0.026072362 | 1,13,14,23,29,35,38 |
| ERCC4 | rs1800067 | Urethritis | 42 | 3 | 7640 | 115 | 0.026265718 | 1,3,16 |
| ERCC4 | rs1800067 | Lng use antiplte/thrmbtc | 42 | 7 | 7640 | 537 | 0.026540445 | 1,5,8,15,16,23,29 |
| ERCC4 | rs1800067 | Other complications of surgical and medical procedures | 42 | 12 | 7640 | 1149 | 0.02709379 | 1,3,5,6,7,9,15,23,25,29,33,39 |
| ERCC4 | rs1800067 | Staghorn calculus | 42 | 7 | 7640 | 544 | 0.028224752 | 1,3,4,12,15,25,34 |
| ERCC4 | rs1800067 | Occlusion of cerebral arteries | 42 | 7 | 7640 | 545 | 0.028471263 | 1,9,15,29,35,38,40 |
| ERCC4 | rs1800067 | Hemorrhage or hematoma complicating a procedure | 42 | 7 | 7640 | 549 | 0.0294722 | 9,15,16,21,24,29,33 |
| ERCC4 | rs1800067 | Stricture of artery | 42 | 3 | 7640 | 122 | 0.030460156 | 1,29,38 |
| ERCC4 | rs1800067 | Other noninfectious disorders of lymphatic channels | 42 | 2 | 7640 | 50 | 0.032568371 | 1,14 |
| ERCC4 | rs1800067 | Atherosclerosis of bypass graft | 42 | 2 | 7640 | 51 | 0.033730099 | 1,29 |
| ERCC4 | rs1800067 | Other mycoses | 42 | 12 | 7640 | 1219 | 0.034079513 | 1,2,6,7,9,15,20,21,25,31,33,39 |
| ERCC4 | rs1800067 | Postoperative infection | 42 | 9 | 7640 | 782 | 0.034954082 | 3,5,15,23,25,29,33,37,39 |
| ERCC4 | rs1800067 | Transient cerebral ischemia | 42 | 7 | 7640 | 577 | 0.037163959 | 1,2,6,9,29,35,38 |
| ERCC4 | rs1800067 | Adjustment disorders | 42 | 5 | 7640 | 340 | 0.038659381 | 8,19,26,28,30 |
| ERCC4 | rs1800067 | Postinflammatory pulmonary fibrosis | 42 | 4 | 7640 | 238 | 0.042209681 | 7,16,21,31 |
| ERCC4 | rs1800067 | Other pneumonia | 42 | 4 | 7640 | 240 | 0.043293493 | 1,21,25,31 |
| ERCC4 | rs1800067 | Unspecified disorder of immune mechanism | 42 | 2 | 7640 | 59 | 0.043587938 | 23,31 |
| ERCC4 | rs1800067 | Acute appendicitis with abscess or peritonitis | 42 | 2 | 7640 | 59 | 0.043587938 | 26,31 |
| ERCC4 | rs1800067 | Gastrointestinal hemorrhage | 42 | 6 | 7640 | 472 | 0.043893495 | 2,4,5,9,25,33 |
| ERCC4 | rs1800067 | Hemiplegia | 42 | 4 | 7640 | 242 | 0.044393133 | 1,2,29,38 |
| ERCC4 | rs1800067 | Residual codes; unclassified; all E codes | 42 | 37 | 7640 | 5670 | 0.04949598 | ,23,24,25,26,29,30,31,32,33,34,35,37,38,39,40,42 |
| F5 | rs6031 | Spontaneous abortion | 15 | 3 | 5076 | 35 | 0.000164141 | 5,8,11 |
| F5 | rs6031 | Hypersomnia | 15 | 2 | 5076 | 10 | 0.000525826 | 2,4 |
| F5 | rs6031 | Glaucoma-Open angle | 15 | 4 | 5076 | 179 | 0.001615238 | 1,2,3,6 |
| F5 | rs6031 | Missed abortion | 15 | 2 | 5076 | 26 | 0.002930624 | 8,11 |
| F5 | rs6031 | Acute gastritis | 15 | 3 | 5076 | 102 | 0.003238534 | 4,6,8 |
| F5 | rs6031 | Menstrual disorders | 15 | 5 | 5076 | 420 | 0.00587993 | 3,4,8,9,11 |
| F5 | rs6031 | Other bone disease and musculoskeletal deformities | 15 | 4 | 5076 | 308 | 0.011014151 | 1,4,6,7 |
| F5 | rs6031 | Hypo/Hyper/Dyshidrosis | 15 | 2 | 5076 | 57 | 0.012584515 | 2,7 |
| F5 | rs6031 | Insomnia | 15 | 2 | 5076 | 58 | 0.012996383 | 2,4 |
| F5 | rs6031 | Other inflammatory diseases of female pelvic organs | 15 | 4 | 5076 | 359 | 0.018480278 | 2,3,5,8 |
| F5 | rs6031 | Sleep apnea | 15 | 4 | 5076 | 373 | 0.020982885 | 1,2,4,6 |
| F5 | rs6031 | Fracture of ankle | 15 | 3 | 5076 | 211 | 0.022919095 | 1,3,6 |
| F5 | rs6031 | Calculus of ureter | 15 | 2 | 5076 | 82 | 0.024575365 | 3,12 |
| F5 | rs6031 | Calculus of bile duct | 15 | 2 | 5076 | 83 | 0.025124939 | 1,8 |
| F5 | rs6031 | Immunizations and screening for infectious disease | 15 | 4 | 5076 | 400 | 0.026395711 | 1,3,8,11 |
| F5 | rs6031 | Cataract | 15 | 5 | 5076 | 616 | 0.028046416 | 1,2,3,6,7 |
| F5 | rs6031 | Arthropathy associated with neurological disorders | 15 | 2 | 5076 | 89 | 0.028528387 | 6,7 |
| F5 | rs6031 | Iron deficiency anemias | 15 | 6 | 5076 | 862 | 0.030161061 | 1,2,3,11,12,14 |
| F5 | rs6031 | Other hemorrhage during pregnancy; childbirth and the puerperium | 15 | 2 | 5076 | 93 | 0.0308961 | 5,8 |
| F5 | rs6031 | Blindness and vision defects | 15 | 6 | 5076 | 875 | 0.03225715 | 1,2,3,4,6,11 |
| F5 | rs6031 | Unspecified hypothyroidism | 15 | 5 | 5076 | 640 | 0.032516968 | 1,2,6,8,10 |
| F5 | rs6031 | Occlusion of cerebral arteries | 15 | 2 | 5076 | 98 | 0.033963496 | 1,13 |
| F5 | rs6031 | Aortic aneurysm | 15 | 2 | 5076 | 99 | 0.034591051 | 1,14 |
| F5 | rs6031 | Abdominal aortic aneurysm without mention of rupture | 15 | 2 | 5076 | 99 | 0.034591051 | 1,14 |
| F5 | rs6031 | Other skin disorders | 15 | 2 | 5076 | 108 | 0.040443713 | 2,7 |
| F5 | rs6031 | Spasm of artery | 15 | 4 | 5076 | 471 | 0.04450993 | 1,3,6,7 |
| F5 | rs6031 | Unspecified osteochondropathy | 15 | 5 | 5076 | 706 | 0.047155793 | 1,2,3,4,15 |
| F5 | rs6031 | Acquired foot deformities | 15 | 3 | 5076 | 283 | 0.048291276 | 2,6,7 |
| FBN2 | rs2291628 | Avascular necrosis/osteonecrosis | 62 | 7 | 7375 | 157 | 0.000395768 | 2,3,26,27,29,30,32 |
| FBN2 | rs2291628 | Postoperative infection | 62 | 16 | 7375 | 778 | 0.000593231 | 2,6,12,20,26,27,28,30,34,35,38,43,46,53,55,56 |
| FBN2 | rs2291628 | Localized adiposity | 62 | 4 | 7375 | 65 | 0.00252573 | 19,35,45,55 |
| FBN2 | rs2291628 | Feeding difficulties and mismanagement | 62 | 12 | 7375 | 595 | 0.003822704 | 2,13,26,28,29,31,34,35,45,54,57,62 |
| FBN2 | rs2291628 | Job's syndrome | 62 | 5 | 7375 | 123 | 0.004125714 | 11,30,45,56,62 |
| FBN2 | rs2291628 | Renal artery stenosis | 62 | 5 | 7375 | 126 | 0.004553451 | 30,34,35,54,61 |
| FBN2 | rs2291628 | Nonspecific abnormal findings in stool contents | 62 | 5 | 7375 | 139 | 0.006777884 | 3,10,13,26,31 |
| FBN2 | rs2291628 | Pervasive developmental disorders | 62 | 2 | 7375 | 14 | 0.007611853 | 7,13 |
| FBN2 | rs2291628 | Ovarian dysfunction | 62 | 2 | 7375 | 15 | 0.008580733 | 9,23 |
| FBN2 | rs2291628 | Sexual and gender identity disorders | 62 | 5 | 7375 | 154 | 0.010185337 | 7,10,20,26,34 |
| FBN2 | rs2291628 | Other miscellaneous mental conditions | 62 | 3 | 7375 | 59 | 0.014721778 | 20,28,44 |
| FBN2 | rs2291628 | Occlusion of cerebral arteries | 62 | 8 | 7375 | 384 | 0.015445648 | 4,21,23,26,30,31,34,54 |
| FBN2 | rs2291628 | Fracture of lower limb | 62 | 6 | 7375 | 250 | 0.019264494 | 2,3,6,12,23,38 |
| FBN2 | rs2291628 | Infective arthritis and osteomyelitis (except that caused by TB or STD | 62 | 4 | 7375 | 120 | 0.019529779 | 12,26,38,53 |
| FBN2 | rs2291628 | Peptic ulcer, site unspecified | 62 | 3 | 7375 | 68 | 0.021106752 | 8,12,54 |
| FBN2 | rs2291628 | Infection and inflammation--internal prosthetic device; implant; and g | 62 | 13 | 7375 | 811 | 0.022478655 | 2,5,12,20,26,28,29,30,31,35,37,46,53 |
| FBN2 | rs2291628 | Osteomyelitis | 62 | 8 | 7375 | 415 | 0.023328138 | 12,20,26,28,39,46,53,54 |
| FBN2 | rs2291628 | Other diseases of veins and lymphatics | 62 | 5 | 7375 | 196 | 0.02557752 | 2,7,11,14,26 |
| FBN2 | rs2291628 | Unspecified gastritis and gastroduodenitis | 62 | 5 | 7375 | 203 | 0.029096824 | 2,3,32,37,44 |
| FBN2 | rs2291628 | Parkinsonism | 62 | 3 | 7375 | 78 | 0.02972279 | 2,9,21 |
| FBN2 | rs2291628 | Somatotropin deficiency | 62 | 3 | 7375 | 80 | 0.03163804 | 26,28,35 |
| FBN2 | rs2291628 | Other complications of surgical and medical procedures | 62 | 16 | 7375 | 1133 | 0.032526679 | 2,9,12,13,21,26,28,30,31,35,38,46,53,54,55,56 |
| FBN2 | rs2291628 | Other current conditions classifiable elsewhere of mother, antepartum | 62 | 3 | 7375 | 81 | 0.032619535 | 20,42,50 |
| FBN2 | rs2291628 | Other and unspecified hereditary and degenerative nervous conditions | 62 | 10 | 7375 | 606 | 0.034495605 | 2,5,6,7,9,21,28,30,58,60 |
| FBN2 | rs2291628 | Acquired coagulation factor deficiency | 62 | 4 | 7375 | 144 | 0.034500959 | 12,26,31,35 |
| FBN2 | rs2291628 | Fracture of vertebral column without mention of spinal cord injury | 62 | 6 | 7375 | 289 | 0.035437328 | 2,23,26,32,53,57 |
| FBN2 | rs2291628 | Nonspecific abnormal results of function study of pulmonary system | 62 | 3 | 7375 | 84 | 0.035659105 | 9,31,35 |
| FBN2 | rs2291628 | Stool flecked with blood | 62 | 10 | 7375 | 613 | 0.036313449 | 2,3,8,13,14,16,19,23,31,32 |
| FBN2 | rs2291628 | Generalized anxiety disorder | 62 | 4 | 7375 | 147 | 0.036737542 | 12,14,28,31 |
| FBN2 | rs2291628 | Other malnutrition | 62 | 10 | 7375 | 617 | 0.037406562 | 2,9,30,31,35,45,46,54,56,62 |
| FBN2 | rs2291628 | Anxiety disorders-Panic | 62 | 4 | 7375 | 150 | 0.039056633 | 6,20,21,33 |
| FBN2 | rs2291628 | Traumatic arthropathy | 62 | 3 | 7375 | 89 | 0.041039564 | 2,12,38 |
| FBN2 | rs2291628 | Acquired foot deformities | 62 | 7 | 7375 | 382 | 0.041955752 | 2,5,8,22,23,53,57 |
| FBN2 | rs2291628 | Other endocrine disorders | 62 | 4 | 7375 | 154 | 0.042277388 | 4,29,31,32 |
| FBN2 | rs2291628 | Other back pain and disorders | 62 | 3 | 7375 | 93 | 0.045623454 | 2,20,23 |
| FBN2 | rs2291628 | Other and unspecified metabolic; nutritional; and endocrine disorders | 62 | 7 | 7375 | 391 | 0.046524394 | 4,8,22,23,24,26,27 |
| GPR98 | rs13157270 | Eosinophilic colitis | 48 | 2 | 7407 | 1 | 0.000121292 | 11,45 |
| GPR98 | rs13157270 | Suppurative cholangitis | 48 | 4 | 7407 | 48 | 0.000326507 | 8,11,15,32 |
| GPR98 | rs13157270 | Pericarditis | 48 | 4 | 7407 | 96 | 0.003773615 | 16,31,32,41 |
| GPR98 | rs13157270 | Epilepsy | 48 | 7 | 7407 | 358 | 0.008264814 | 2,7,21,30,32,36,45 |
| GPR98 | rs13157270 | Postinflammatory pulmonary fibrosis | 48 | 5 | 7407 | 240 | 0.019941687 | 3,11,31,32,45 |
| GPR98 | rs13157270 | Hypogammaglobulinemia, unspecified | 48 | 2 | 7407 | 32 | 0.019977431 | 41,45 |
| GPR98 | rs13157270 | Other and unspecified lower respiratory disease | 48 | 5 | 7407 | 241 | 0.020258653 | 3,5,14,21,41 |
| GPR98 | rs13157270 | Other specified idiopathic peripheral neuropathy | 48 | 3 | 7407 | 89 | 0.021087019 | 8,24,26 |
| GPR98 | rs13157270 | Idiopathic progressive polyneuropathy | 48 | 8 | 7407 | 554 | 0.025608198 | 8,10,24,26,27,28,32,38 |
| GPR98 | rs13157270 | LBBB | 48 | 3 | 7407 | 105 | 0.031928575 | 20,21,47 |
| GPR98 | rs13157270 | Unspecified chronic liver disease without mention of alcohol | 48 | 2 | 7407 | 42 | 0.032347542 | 15,35 |
| GPR98 | rs13157270 | Withdrawal arrhythmia | 48 | 2 | 7407 | 45 | 0.03651769 | 4,21 |
| GPR98 | rs13157270 | Sprains and strains | 48 | 2 | 7407 | 1087 | 0.038701479 | 28,46 |
| GPR98 | rs13157270 | Atrophic gastritis | 48 | 3 | 7407 | 116 | 0.04078723 | 11,14,45 |
| GPR98 | rs13157270 | Other asthma with status asthmaticus | 48 | 4 | 7407 | 200 | 0.041531976 | 5,6,17,28 |
| HGFAC | rs16844401 | Gastroesophageal laceration-hemorrhage syndrome | 32 | 2 | 7684 | 11 | 0.001263253 | 7,8 |
| HGFAC | rs16844401 | Hypertension | 32 | 21 | 7684 | 3064 | 0.003626206 | ,32 |
| HGFAC | rs16844401 | Hematemesis | 32 | 4 | 7684 | 173 | 0.00582776 | 5,7,8,20 |
| HGFAC | rs16844401 | Gastrointestinal hemorrhage | 32 | 6 | 7684 | 476 | 0.012990636 | 5,7,8,9,11,14 |
| HGFAC | rs16844401 | Rheumatoid arthritis and related disease | 32 | 4 | 7684 | 328 | 0.046857392 | 7,15,21,23 |
| PLCG2 | rs17537869 | Other specified arthropathy, site unspecified | 11 | 2 | 4919 | 18 | 0.000841428 | 7,8 |
| PLCG2 | rs17537869 | Pseudoaneurysm | 11 | 2 | 4919 | 51 | 0.005862109 | 10,11 |
| PLCG2 | rs17537869 | Allergic reactions-Skin | 11 | 5 | 4919 | 730 | 0.015379266 | 1,2,3,4,8 |
| PLCG2 | rs17537869 | Intracranial aneurysm | 11 | 2 | 4919 | 86 | 0.015604976 | 10,11 |
| PLCG2 | rs17537869 | Bipolar disorders | 11 | 3 | 4919 | 291 | 0.024214668 | 2,6,7 |
| PTAFR | rs5939 | Acute upper respiratory infections of multiple or unspecified sites | 17 | 11 | 8337 | 1288 | 6.16E-06 | 1,2,3,4,5,8,9,10,13,16,17 |
| PTAFR | rs5939 | Cervicitis and endocervicitis | 17 | 4 | 8337 | 124 | 0.000107225 | 2,3,4,17 |
| PTAFR | rs5939 | Other and unspecified diseases of ureters | 17 | 10 | 8337 | 1502 | 0.000206263 | 1,2,6,7,8,9,12,13,15,16 |
| PTAFR | rs5939 | Menopausal disorders | 17 | 7 | 8337 | 777 | 0.00052316 | 1,2,3,5,8,10,13 |
| PTAFR | rs5939 | Other and ill-defined heart disease | 17 | 8 | 8337 | 1044 | 0.000523478 | 1,2,3,5,6,8,9,13 |
| PTAFR | rs5939 | Other injuries and conditions due to external causes | 17 | 13 | 8337 | 2921 | 0.000604288 | 1,2,3,4,5,6,8,10,11,12,13,14,16 |
| PTAFR | rs5939 | Sclerosing glomerulonephritis | 17 | 3 | 8337 | 86 | 0.000713448 | 7,8,9 |
| PTAFR | rs5939 | Epistaxis | 17 | 4 | 8337 | 244 | 0.001329703 | 1,2,3,16 |
| PTAFR | rs5939 | Atherosclerosis of arteries of extremities | 17 | 5 | 8337 | 434 | 0.001431537 | 5,7,10,13,15 |
| PTAFR | rs5939 | Cataract | 17 | 7 | 8337 | 966 | 0.001924598 | 1,2,10,12,13,14,15 |
| PTAFR | rs5939 | Acute bronchitis | 17 | 6 | 8337 | 745 | 0.002693688 | 1,2,8,9,10,14 |
| PTAFR | rs5939 | Other and unspecified genitourinary symptoms | 17 | 12 | 8337 | 2718 | 0.002715184 | 1,2,3,4,6,7,8,9,10,13,14,17 |
| PTAFR | rs5939 | Angina pectoris | 17 | 14 | 8337 | 3822 | 0.002790642 | 1,2,3,4,5,6,7,8,9,10,13,14,15,16 |
| PTAFR | rs5939 | Gastrointestinal hemorrhage | 17 | 5 | 8337 | 517 | 0.003082102 | 1,6,8,10,16 |
| PTAFR | rs5939 | Other specified anemia | 17 | 12 | 8337 | 2832 | 0.003121249 | 1,2,3,4,6,7,8,9,10,13,15,16 |
| PTAFR | rs5939 | Other connective tissue disease | 17 | 7 | 8337 | 1054 | 0.003194671 | 1,2,3,6,8,10,14 |
| PTAFR | rs5939 | Other disorders of plasma protein metabolism | 17 | 3 | 8337 | 147 | 0.003207332 | 5,7,8 |
| PTAFR | rs5939 | Hypoglycemia | 17 | 4 | 8337 | 318 | 0.003461395 | 1,2,3,9 |
| PTAFR | rs5939 | Glaucoma-closed angle | 17 | 2 | 8337 | 43 | 0.003665414 | 10,13 |
| PTAFR | rs5939 | Other upper respiratory disease | 17 | 10 | 8337 | 2179 | 0.00446104 | 1,2,3,4,8,9,10,12,14,16 |
| PTAFR | rs5939 | Membranous glomerulonephritis | 17 | 2 | 8337 | 50 | 0.00486855 | 7,8 |
| PTAFR | rs5939 | Immunizations and screening for infectious disease | 17 | 12 | 8337 | 3063 | 0.005249103 | 1,2,3,5,7,8,9,10,11,12,13,14 |
| PTAFR | rs5939 | Proliferative glomerulonephritis | 17 | 2 | 8337 | 53 | 0.005432836 | 8,9 |
| PTAFR | rs5939 | Other and unspecified upper respiratory infections | 17 | 8 | 8337 | 1504 | 0.00582658 | 1,2,3,4,8,9,10,14 |
| PTAFR | rs5939 | Other and ill-defined cerebrovascular disease | 17 | 4 | 8337 | 377 | 0.006314932 | 1,2,3,15 |
| PTAFR | rs5939 | Acute renal failure | 17 | 8 | 8337 | 1532 | 0.006542676 | 2,6,7,8,9,13,15,16 |
| PTAFR | rs5939 | Meningitis (except that caused by TB or STD) | 17 | 3 | 8337 | 192 | 0.006689251 | 3,6,14 |
| PTAFR | rs5939 | Chronic sinusitis | 17 | 6 | 8337 | 906 | 0.007139105 | 1,3,9,10,11,14 |
| PTAFR | rs5939 | Other ear and sense organ disorders | 17 | 6 | 8337 | 929 | 0.008064602 | 2,4,5,9,10,11 |
| PTAFR | rs5939 | Diabetic retinopathy | 17 | 4 | 8337 | 410 | 0.008453822 | 1,2,13,15 |
| PTAFR | rs5939 | Vasovagal attack | 17 | 6 | 8337 | 968 | 0.009833705 | 1,2,3,13,14,16 |
| PTAFR | rs5939 | Lack norm physio dev NOS | 17 | 2 | 8337 | 74 | 0.010168618 | 6,7 |
| PTAFR | rs5939 | Conduction disorders | 17 | 3 | 8337 | 240 | 0.012222418 | 1,2,5 |
| PTAFR | rs5939 | Eosinophilia | 17 | 2 | 8337 | 83 | 0.012601601 | 1,9 |
| PTAFR | rs5939 | Coronary atherosclerosis | 17 | 8 | 8337 | 1721 | 0.013375099 | 1,2,3,5,9,12,14,15 |
| PTAFR | rs5939 | Urinary tract infection, site not specified | 17 | 10 | 8337 | 2450 | 0.013612227 | 2,3,4,6,8,9,10,13,15,17 |
| PTAFR | rs5939 | Other connective tissue disease | 17 | 13 | 8337 | 3824 | 0.013798577 | 1,2,3,4,6,7,8,9,10,12,13,14,15 |
| PTAFR | rs5939 | Nephropathy | 17 | 4 | 8337 | 484 | 0.014889364 | 2,8,9,13 |
| PTAFR | rs5939 | Other inflammatory diseases of female pelvic organs | 17 | 4 | 8337 | 494 | 0.015947711 | 1,2,3,4 |
| PTAFR | rs5939 | Other nonspecific abnormal serum enzyme levels | 17 | 6 | 8337 | 1077 | 0.016286632 | 2,3,6,8,13,14 |
| PTAFR | rs5939 | Intracranial hemorrhage | 17 | 3 | 8337 | 268 | 0.016386339 | 1,3,14 |
| PTAFR | rs5939 | Iron deficiency anemias | 17 | 6 | 8337 | 1103 | 0.018192491 | 3,4,6,9,10,13 |
| PTAFR | rs5939 | Deficiency and other anemia | 17 | 6 | 8337 | 1105 | 0.018345417 | 2,3,6,7,9,16 |
| PTAFR | rs5939 | Intermediate coronary syndrome | 17 | 4 | 8337 | 518 | 0.018682727 | 1,2,14,15 |
| PTAFR | rs5939 | Nephrotic syndrome | 17 | 2 | 8337 | 105 | 0.019500201 | 7,8 |
| PTAFR | rs5939 | Other eye disorders | 17 | 7 | 8337 | 1465 | 0.019608948 | 1,2,3,4,10,13,15 |
| PTAFR | rs5939 | Other fluid and electrolyte disorders | 17 | 6 | 8337 | 1126 | 0.020007077 | 2,3,6,7,8,13 |
| PTAFR | rs5939 | Diseases of the digestive system | 17 | 5 | 8337 | 820 | 0.020951949 | 2,6,9,10,14 |
| PTAFR | rs5939 | Observation for suspected cardiovascular disease | 17 | 9 | 8337 | 2149 | 0.021513645 | 1,2,3,4,6,8,13,14,17 |
| PTAFR | rs5939 | Other late effects of cerebrovascular disease | 17 | 2 | 8337 | 118 | 0.024174771 | 2,3 |
| PTAFR | rs5939 | Acute and chronic tonsillitis | 17 | 2 | 8337 | 118 | 0.024174771 | 4,9 |
| PTAFR | rs5939 | Other non-traumatic joint disorders-Unspecified | 17 | 3 | 8337 | 318 | 0.025614368 | 3,4,13 |
| PTAFR | rs5939 | Hypertensive heart and/or renal disease | 17 | 7 | 8337 | 1545 | 0.025819387 | 2,6,7,8,9,13,15 |
| PTAFR | rs5939 | Acute but ill-defined cerebrovascular accident | 17 | 3 | 8337 | 320 | 0.026031975 | 1,2,14 |
| PTAFR | rs5939 | Abnormal weight gain | 17 | 3 | 8337 | 320 | 0.026031975 | 2,8,16 |
| PTAFR | rs5939 | Superficial injury; contusion | 17 | 6 | 8337 | 1199 | 0.026616138 | 2,3,4,8,9,12 |
| PTAFR | rs5939 | Other skin disorders-Nail and hair | 17 | 4 | 8337 | 582 | 0.027382499 | 1,2,8,13 |
| PTAFR | rs5939 | Other deficiency anemia | 17 | 2 | 8337 | 127 | 0.02765594 | 4,10 |
| PTAFR | rs5939 | Chronic renal failure-Stage I-III | 17 | 7 | 8337 | 1570 | 0.028023549 | 2,6,7,8,9,13,15 |
| PTAFR | rs5939 | Inflammation; infection of eye (except that caused by TB or STD) | 17 | 5 | 8337 | 898 | 0.029854507 | 2,3,10,13,15 |
| PTAFR | rs5939 | Uremic coma | 17 | 4 | 8337 | 599 | 0.030050821 | 2,6,15,16 |
| PTAFR | rs5939 | Disorders of the peripheral nervous system | 17 | 6 | 8337 | 1237 | 0.030599266 | 2,4,5,8,14,15 |
| PTAFR | rs5939 | Gangrene | 17 | 2 | 8337 | 138 | 0.032170505 | 3,13 |
| PTAFR | rs5939 | Thrombophilia | 17 | 4 | 8337 | 616 | 0.032873541 | 2,3,6,16 |
| PTAFR | rs5939 | Other mycoses | 17 | 6 | 8337 | 1286 | 0.036319219 | 1,2,3,4,13,16 |
| PTAFR | rs5939 | Late effects of cerebrovascular disease | 17 | 3 | 8337 | 365 | 0.036418385 | 2,3,14 |
| PTAFR | rs5939 | Nonspecific abnormal findings in stool contents | 17 | 2 | 8337 | 148 | 0.036512249 | 6,8 |
| PTAFR | rs5939 | Transient cerebral ischemia | 17 | 4 | 8337 | 642 | 0.037493294 | 1,2,3,14 |
| PTAFR | rs5939 | Hydrocephralus | 17 | 2 | 8337 | 156 | 0.040141845 | 3,14 |
| PTAFR | rs5939 | Other polyp of sinus | 17 | 3 | 8337 | 384 | 0.041370663 | 3,9,14 |
| PTAFR | rs5939 | Unspecified immunity deficiency | 17 | 3 | 8337 | 401 | 0.046083867 | 2,8,16 |
| PTAFR | rs5939 | Other and unspecified noninfectious gastroenteritis and colitis | 17 | 4 | 8337 | 687 | 0.04636665 | 4,6,8,9 |
| PTAFR | rs5939 | Phlebitis and thrombophlebitis | 17 | 3 | 8337 | 412 | 0.04927424 | 6,13,14 |
| TAAR1 | rs8192619 | Nonmalignant breast conditions | 17 | 8 | 4306 | 610 | 0.001228559 | 1,2,3,6,9,10,11,12 |
| TAAR1 | rs8192619 | Other atopic dermatitis and related conditions | 17 | 3 | 4306 | 80 | 0.003821395 | 2,3,5 |
| TAAR1 | rs8192619 | Anxiety disorders | 17 | 6 | 4306 | 471 | 0.007462053 | 2,5,6,8,11,17 |
| TAAR1 | rs8192619 | Anemia during pregnancy | 17 | 2 | 4306 | 32 | 0.00758441 | 3,5 |
| TAAR1 | rs8192619 | Other hypertension in pregnancy | 17 | 2 | 4306 | 35 | 0.008941833 | 3,5 |
| TAAR1 | rs8192619 | Depressive disorders | 17 | 9 | 4306 | 1032 | 0.009468961 | 1,2,3,5,6,8,10,11,16 |
| TAAR1 | rs8192619 | Localized adiposity | 17 | 2 | 4306 | 37 | 0.009903 | 3,6 |
| TAAR1 | rs8192619 | Normal delivery | 17 | 2 | 4306 | 41 | 0.011957223 | 3,5 |
| TAAR1 | rs8192619 | Nonspecific abnormal findings on radiological and other examination of | 17 | 3 | 4306 | 127 | 0.013269241 | 1,13,17 |
| TAAR1 | rs8192619 | Backache; unspecified | 17 | 7 | 4306 | 724 | 0.015677128 | 1,2,3,5,8,10,17 |
| TAAR1 | rs8192619 | Other skin disorders | 17 | 8 | 4306 | 918 | 0.016351038 | 1,2,3,5,6,8,14,17 |
| TAAR1 | rs8192619 | Nausea and vomiting | 17 | 8 | 4306 | 935 | 0.018222775 | 1,3,5,8,9,10,13,17 |
| TAAR1 | rs8192619 | Other and unspecified complications of pregnancy | 17 | 2 | 4306 | 57 | 0.021836835 | 3,5 |
| TAAR1 | rs8192619 | Chronic pancreatitis | 17 | 2 | 4306 | 57 | 0.021836835 | 13,17 |
| TAAR1 | rs8192619 | Abdominal aortic aneurysm without mention of rupture | 17 | 2 | 4306 | 59 | 0.023248387 | 7,14 |
| TAAR1 | rs8192619 | Other and unspecified complications of birth; puerperium affecting man | 17 | 2 | 4306 | 68 | 0.030052102 | 3,5 |
| TAAR1 | rs8192619 | Diseases of the digestive system | 17 | 8 | 4306 | 965 | 0.034902384 | 1,2,3,5,8,9,10,17 |
| TAAR1 | rs8192619 | Abnormal weight gain | 17 | 3 | 4306 | 185 | 0.035094203 | 1,3,5 |
| TAAR1 | rs8192619 | Occlusion of cerebral arteries | 17 | 3 | 4306 | 191 | 0.038022033 | 1,8,14 |
| TAAR1 | rs8192619 | Abnormal loss of weight | 17 | 4 | 4306 | 343 | 0.042038441 | 1,3,12,17 |
| TAAR1 | rs8192619 | Osteopenia | 17 | 2 | 4306 | 89 | 0.04856161 | 8,11 |
